# Arene Selectivity by a Flexible Coordination Polymer Host

**DOI:** 10.1002/chem.201601870

**Published:** 2016-08-02

**Authors:** James S. Wright, Iñigo J. Vitórica‐Yrezábal, Stephen P. Thompson, Lee Brammer

**Affiliations:** ^1^ Department of ChemistryUniversity of Sheffield, Brook HillSheffieldS3 7HFUK), Fax: (+44) 114-2229346; ^2^ School of ChemistryUniversity of Manchester, Oxford RoadManchesterM13 9PLUK; ^3^ Diamond Light Source, Harwell Science and Innovation Campus, DidcotOxfordshireOX11 0DEUK

**Keywords:** arene separation, coordination polymer, crystal engineering, powder diffraction, xylenes

## Abstract

The coordination polymers [Ag_4_(O_2_CCF_3_)_4_(phen)_3_]**⋅** phen**⋅**arene (**1⋅**phen**⋅**arene) (phen=phenazine; arene=toluene, *p*‐xylene or benzene) have been synthesised from the solution phase in a series of arene solvents and crystallographically characterised. By contrast, analogous syntheses from *o*‐xylene and *m*‐xylene as the solvent yield the solvent‐free coordination polymer [Ag_4_(O_2_CCF_3_)_4_(phen)_2_] (**2**). Toluene, *p*‐xylene and benzene have been successfully used in mixed‐arene syntheses to template the formation of coordination polymers **1⋅**phen**⋅**arene, which incorporate *o*‐ or *m*‐xylene. The selectivity of **1⋅**phen**⋅**arene for the arene guests was determined, through pairwise competition experiments, to be *p*‐xylene>toluene≈benzene>*o*‐xylene>*m*‐xylene. The largest selectivity coefficient was determined as 14.2 for *p*‐xylene:*m*‐xylene and the smallest was 1.0 for toluene:benzene.

## Introduction

Materials that are porous on the molecular scale have been in use for many years in applications involving molecular separation. Fixed‐pore materials, exemplified by zeolites and related inorganic porous materials,^1^ have been joined over the past 15–20 years by a number of new classes materials of porous materials, most prominently metal–organic frameworks (MOFs),^2^, ^3^ but also covalent organic frameworks (COFs)^4^ and other polymeric or framework materials.^5^ These materials have the advantage of being modular in design, enabling tunability of properties, including pore size, shape and chemical composition. Although a growing number of dynamic framework materials with flexible pores are being reported,^6^, ^7^ most MOFs and similar materials have a rigid pore structure. Greater flexibility, although typically with smaller guest adsorption capacity, is seen in molecular materials, in which either the molecules themselves contain interior voids or the packing of molecules enables voids to be generated between them in crystalline solids.^8^, ^9^, ^10^, ^11^, ^12^ Many of this last class of materials, although lacking conventional porosity, may be described as exhibiting latent porosity, whereby guest uptake is combined with molecular mobility in the solid state, which enables guest encapsulation.^13^, ^14^, ^15^, ^16^, ^17^

**Scheme 1:**
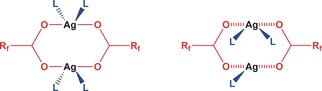
Examples of (flexible) silver(I) perfluorocarboxylate dimer secondary building units, connected by neutral ditopic ligands, L (in the present study, phenazine), to propagate coordination polymers.

We have developed a class of 1D coordination polymers based on silver(I) perfluoroalkylcarboxylate dimer units linked through diimine ligands, such as substituted pyrazine or phenazine (Scheme 1), that are able to trap small molecules between the polymers and exchange these guests in a reversible manner.^16^ These materials are crystalline and the guest exchange proceeds with retention of crystallinity, allowing the process to be followed by in situ diffraction studies in addition to a variety of other physical methods. Recently, we reported the encapsulation of small arene guests (toluene, xylenes) in one such coordination polymer [Ag_4_{O_2_C(CF_2_)_2_CF_3_}_4_(phen)_2_(arene)_*n*_]**⋅** *m* (arene) (phen=phenazine) and examined the role of these guests in templating solid‐state transformations.^17^ In the present study we explore the encapsulation of benzene, toluene, *o*‐xylene, *m*‐xylene and *p*‐xylene by the coordination polymer [Ag_4_(O_2_CCF_3_)_4_(phen)_3_] (**1**) during its self‐assembly from the solution phase. This results in the crystalline materials [Ag_4_(O_2_CCF_3_)_4_(phen)_3_]**⋅**phen**⋅**arene (**1⋅**phen**⋅**arene), in which the arenes act as co‐guests alongside non‐coordinated phenazine. Specifically, we are able to establish the selectivity of the coordination polymer for each of the five arenes by determination of the pairwise selectivity coefficients, and are able to determine the potential of this material in separation of structurally similar arenes by recycling of the encapsulation process.

The separation of small arenes (e.g., xylenes) is important commercially due to the large scale on which such compounds are synthesised for use as precursors in the chemical industry, combined with the non‐regiospecific manner in which alkylarenes, such as xylenes, are synthesised. Their similarity in physical properties (e.g., b.p.) makes conventional methods of separation, such as distillation, a difficult and not very cost‐effective approach.10a Separation by zeolites and MOFs has been investigated. More recently, however, there has been increased interest in exploring molecular materials^10^, ^18^ or flexible MOFs with more adaptable pore spaces for such separations.^19^

## Results and Discussion

### Arene uptake or exclusion in self‐assembly of 1⋅phen⋅arene

**Figure 1:**
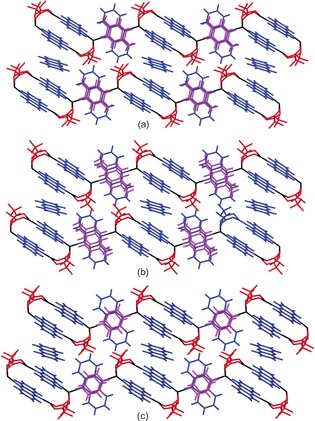
Crystal structures of a) **1⋅**phen**⋅**tol, b) **1⋅**phen**⋅**pxyl and c) **1⋅**phen**⋅** benz, showing two adjacent polymer tapes with alternating singly‐ and doubly‐bridging phenazine units and arene guests. Silver atoms shown in black, trifluoroacetate in red, phenazine in blue and toluene, *p*‐xylene or benzene in magenta. Only one component of the rotationally disordered CF_3_ groups is shown.

Layering of a solution of silver(I) trifluoroacetate in methanol onto a solution of phenazine dissolved in either toluene, *p*‐xylene or benzene resulted in exclusive formation of the corresponding arene‐guest‐containing 1D coordination polymer [Ag_4_(O_2_CCF_3_)_4_(phen)_3_]**⋅**phen**⋅**arene (**1⋅**phen**⋅**arene). Phase purity was confirmed by elemental analysis and Pawley fitting of the corresponding X‐ray powder patterns. Crystal structures of [Ag_4_(O_2_CCF_3_)_4_(phen)_3_]**⋅**phen**⋅**2 (toluene) (**1⋅**phen**⋅**tol), [Ag_4_(O_2_CCF_3_)_4_(phen)_3_]**⋅**phen**⋅**2 (*p*‐xylene) (**1⋅**phen**⋅**pxyl) and [Ag_4_(O_2_CCF_3_)_4_(phen)_3_]**⋅**phen**⋅**2 C_6_H_6_ (**1⋅**phen**⋅**benz) were determined by single‐crystal X‐ray diffraction (Figure 1). Each structure comprises coordination polymers constructed from silver(I) trifluoroacetate dimers, which are linked by bridging phenazine ligands, leading to propagation of a 1D zigzag tape. The phenazine linkers alternate between singly‐ and doubly‐bridging motifs (Scheme 1 b), with the planes of alternate phenazine units oriented orthogonal to each other (Figure 1). This polymeric arrangement is analogous to the structure of silver(I) perfluoroalkylcarboxylate coordination polymers of the formula [Ag_4_(O_2_CR_f_)_4_(L)_3_] (*R*_f_=perfluoroalkyl group; L=diimine ligand) described in our previous work.^16^, ^17^ In each **1⋅**phen**⋅**arene material additional non‐coordinated phenazine molecules are included as guests, situated between each of the doubly‐bridging phenazine linkers in a π‐stacked manner. Two equivalents of the arene used as solvent are also present as guests per repeat unit of the polymer. These molecules (toluene, *p*‐xylene or benzene) are π‐stacked on both sides of the electron‐deficient central ring of the singly‐bridging phenazine ligands. The arenes are crystallographically ordered and each arene molecule is related to another by a centre of symmetry located in the centre of those phenazine ligands (Figure 1).

**Figure 2:**
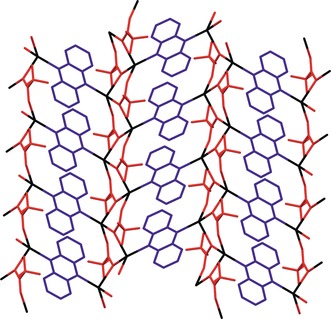
Crystal structure of the two‐dimensional coordination polymer, **2**. Colours as in Figure 1. Hydrogen atoms omitted for clarity.

Analogous syntheses conducted using *o*‐ and *m*‐xylene, however, did not yield the analogous 1D coordination polymer. Rather, these syntheses led exclusively to the 2D coordination polymer [Ag_4_(O_2_CCF_3_)_4_(phen)_2_] (**2**), which excludes the xylene guests. This more densely‐packed phase is propagated in one dimension by an extended arrangement of silver perfluoroacetate units that employ both the *anti* and *syn* lone pairs on the carboxylate oxygen in coordination to Ag^I^ centres,^20^ and in the second dimension through bridging phenazine ligands (Figure 2). The structural motif is analogous to 2D materials generated from cross‐linking of 1D coordination polymers by loss of solvent guest molecules in some of our earlier studies of silver(I) carboxylate coordination polymers containing tetramethylpyrazine^16^ or phenazine^17^ linker ligands.

### Selectivity studies: toluene, *p*‐xylene and benzene

Having demonstrated that three of the arenes investigated (toluene, *p*‐xylene and benzene) were included in the self‐assembly of **1⋅**phen**⋅**arene, the selectivity of this inclusion process was examined by means of pairwise competition experiments between the three arenes. This was achieved by conducting the assembly of **1⋅**phen**⋅**arene in the presence of a 1:1 mixture (by volume) of two of the three possible pairs of arenes. Pawley fitting of X‐ray powder diffraction confirmed the formation of **1⋅**phen**⋅**arene, along with a very small amount of **2**.^21^ Although single crystals of each product were also obtained, the disordered arene guest content could not be fully modelled by single‐crystal X‐ray diffraction, but the model suggested the inclusion of more than one of the arenes as guests. The relative inclusion of the two arenes in each study was determined quantitatively by digesting the crystals in [D_6_]DMSO, and studying the resulting solution by ^1^H NMR spectroscopy and gas chromatography (GC). These data and their analyses are presented in full in the Supporting Information. Pairwise selectivity constants, *K*_A:B_ were determined from the corresponding inclusion experiments [see Eq. ((1))],^22^ each constant being determined as an average of at least four measurements,^23^ and are summarised in Table 1. The results show the selectivity of the coordination polymer host for *p*‐xylene over toluene and benzene, but no measurable selectivity between toluene and benzene (i.e. selectivity of *p*‐xylene>toluene≈benzene).

(1) $$K_{A:B}={\left(K_{B:A}\right)}^{-1}=\left(\frac{Y_{A}}{Y_{B}}\right)\left(\frac{X_{B}}{X_{A}}\right)\;\;\left(X{}_{A}+X{}_{B}=Y{}_{A}+Y{}_{B}=1\right)$$

**Table 1:** Selectivity constants for pairwise competition experiments involving inclusion of the arenes in **1⋅**phen**⋅**arene. *X*_A_ is the mole fraction of A (volume fraction is 0.50 in all cases).

Guest A	Guest B	*X*_A_	*X*_B_	*Y*_A_	*Y*_B_	*K*_A:B_
toluene	benzene	0.46	0.54	0.46(2)	0.54(2)	0.98(8)
*p*‐xylene	benzene	0.42	0.58	0.58(2)	0.42(2)	1.87(12)
*p*‐xylene	toluene	0.77	0.23	0.86(2)	0.14(2)	1.90(29)
*p*‐xylene	toluene	0.56	0.44	0.70(3)	0.30(3)	1.86(30)
*p*‐xylene	toluene	0.46	0.54	0.62(4)	0.38(4)	1.89(28)
*p*‐xylene	toluene	0.36	0.64	0.51(4)	0.49(4)	1.84(30)
*p*‐xylene	toluene	0.18	0.82	0.27(3)	0.73(3)	1.71(29)
*p*‐xylene	toluene					1.84(8) average

**Figure 3:**
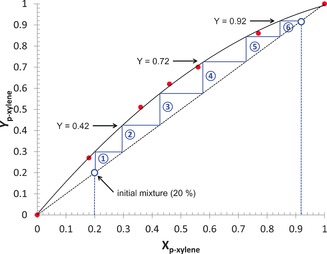
McCabe–Thiele type plot of mole fraction (*X*_*p*‐xylene_) of *p*‐xylene, used in the synthesis of [Ag_4_(O_2_CCF_3_)_4_(phen)_3_]**⋅**phen**⋅**2 {(tol)_*x*_(pxyl)_1−*x*_} (**1⋅**phen**⋅** tol**⋅**pxyl), against the mole fraction (*Y*_*p*‐xylene_) found in the product. The plot illustrates that a sample of *p*‐xylene (92 % pure) may be obtained through six crystallisation/filtration steps from an initial mixture containing only 20 % *p*‐xylene and 80 % toluene.

The selectivity of **1⋅**phen**⋅**arene for *p*‐xylene over toluene was examined in more detail by varying the ratio of toluene and *p*‐xylene used in assembly of the coordination polymer. Plotting these selectivity data as a McCabe–Thiele type plot^24^ (Figure 3) indicates that a mixture of *p*‐xylene and toluene that is initially only 20 mol % *p*‐xylene may be purified to be 92 mol % *p*‐xylene by inclusion in **1⋅**phen**⋅**arene in six crystallisation steps. Analogous studies have been performed by Ward and co‐workers in assessing the behaviour of supramolecular hydrogen‐bonded guanidinium disulfonate hosts for separation of xylenes and isomers of dimethylnapthalene.^18^

### Selectivity studies: *o*‐xylene and *m*‐xylene

Although single arene guest syntheses did not enable inclusion of *o*‐xylene or *m*‐xylene, mixed‐arene syntheses were conducted using these xylenes. Use of a 1:1 mixture of *o*‐xylene:*m*‐xylene exclusively yielded **2**, as observed when these arenes are used alone. Mixed syntheses involving 1:1 volume ratios of either *o*‐xylene or *m*‐xylene with one of *p*‐xylene, toluene or benzene, however, led to the formation of the coordination polymer **1⋅**phen**⋅**arene, as confirmed by Pawley fitting of the PXRD data for the product. As in most previous syntheses the presence of very small amounts of **2** was also evident.^21^ Digestion of the crystalline product and analysis by ^1^H NMR spectroscopy and GC confirmed the presence of both arenes, indicating that the presence of one of the arenes that is more readily included in **1⋅**phen**⋅**arene enables the inclusion of *o*‐xylene or *m*‐xylene. All experimental data and analyses are provided in Supporting Information and selectivity coefficients are given in Table 2.

**Table 2:** Selectivity constants for pairwise competition experiments involving inclusion in **1⋅**phen**⋅**arene of the arenes *p*‐xylene, toluene or benzene in competition with *o*‐xylene or *m*‐xylene.

Guest A	Guest B	*X*_A_	*X*_B_	*Y*_A_	*Y*_B_	*K*_A:B_
*p*‐xylene	*o*‐xylene	0.49	0.51	0.898(1)	0.102(1)	9.13(13)
*p*‐xylene	*m*‐xylene	0.50	0.50	0.93(2)	0.07(2)	14.2(24)
toluene	*o*‐xylene	0.53	0.47	0.79(3)	0.21(3)	3.26(56)
toluene	*m*‐xylene	0.54	0.46	0.87(2)	0.13(2)	5.63(94)
benzene	*o*‐xylene	0.58	0.42	0.83(2)	0.17(2)	3.59(61)
benzene	*m*‐xylene	0.58	0.42	0.93(2)	0.07(2)	9.3(23)

The selectivity coefficients for these inclusion experiments are much larger than those between *p*‐xylene, toluene and benzene, confirming the more facile inclusion of these three arenes compared to *o*‐xylene or *m*‐xylene. The selectivity constants for the uptake of *p*‐xylene, toluene and benzene versus *m*‐xylene are greater than the selectivity constants for their uptake versus *o*‐xylene, suggesting that *m*‐xylene is the least favourable guest of all.

## Conclusion

A one‐dimensional coordination polymer, **1⋅**phen**⋅**arene, which is selective between different arenes through their incorporation during its self‐assembly, has been synthesised and crystallographically characterised. The material directly entraps toluene, *p*‐xylene or benzene, but not *o*‐xylene and *m*‐xylene, when presented with a single arene. Use of *o*‐ or *m*‐xylene instead leads to formation of the two‐dimensional coordination polymer **2**, which contains no arene guest. When assembly takes place in the presence of two of the arenes in a 1:1 volumetric ratio both arenes are incorporated, but the polymer is selective for one of the arenes (although no selectivity between toluene and benzene can be discerned). By this approach either *o*‐xylene or *m*‐xylene can also be included as guests when accompanied by one the other three arenes. Competition experiments have enabled pairwise selectivity coefficients to be determined. The largest selectivity coefficients is 14.2 for *p*‐xylene:*m*‐xylene and the smallest is 1.0 for toluene:benzene.

## Experimental Section

### Crystal syntheses

All starting materials were purchased from Aldrich, Alfa Aesar or Fluorochem and used as received. Light was excluded from all reactions using aluminium foil to minimise decomposition to silver metal. In each case, 0.05 m solutions of the reagents were separately prepared by dissolving silver(I) trifluoroacetate (92 mg, 0.4 mmol) or phenazine (72 mg, 0.4 mmol) in solvent (8 mL). In all cases, large yellow crystals suitable for single‐crystal X‐ray diffraction were formed within one week. In the case of guest competition experiments, exactly seven days was allowed for crystallisation before analysing the guest content. For all syntheses using mixtures of arenes, X‐ray powder diffraction indicates a small amount of coordination polymer **2** as a byproduct. Yield calculations assume a single product and therefore for the mixed‐arene studies are approximate. Elemental analyses were not conducted for materials involving *o*‐xylene or *m*‐xylene, which contained a larger amount of **2** as a byproduct from inspection of PXRD patterns.

**[Ag_4_(O_2_CCF_3_)_4_(phen)_3_]⋅phen⋅2 (toluene) (1⋅phen⋅tol)**: A 0.05 m solution of AgO_2_CCF_3_ (92 mg, 0.40 mmol) in methanol (8 mL) was layered on to a 0.05 m solution of phenazine (72 mg, 0.40 mmol) in toluene (8 mL). Yield 65 % (110 mg, 0.065 mmol); elemental analysis calcd (%): C, 47.00, H 2.70, N 6.26; found: C 46.88, H 2.25, N 6.21.

**[Ag_4_(O_2_CCF_3_)_4_(phen)_3_]⋅phen⋅2 (*p*‐xylene) (1⋅phen⋅pxyl)**: A 0.05 m solution of AgO_2_CCF_3_ (92 mg, 0.40 mmol) in methanol (8 mL) was layered on to a 0.05 m solution of phenazine (72 mg, 0.40 mmol) in *p*‐xylene (8 mL). Yield 56 % (95 mg, 0.056 mmol); elemental analysis calcd (%): C 47.60, H 2.89, N 6.17; found: C 47.35, H 2.57, N 6.11.

**[Ag_4_(O_2_CCF_3_)_4_(phen)_3_]⋅phen⋅2 C_6_H_6_ (1⋅phen⋅benz)**: A 0.05 m solution of AgO_2_CCF_3_ (92 mg, 0.4 mmol) in methanol (8 mL) was layered on to a 0.05 m solution of phenazine (72 mg, 0.4 mmol) in benzene (8 mL). Yield 54 % (91 mg, 0.054 mmol); elemental analysis calcd (%): C 46.39, H 2.52, N 6.36; found: C 46.42, H 2.12, N 6.29.

**[Ag_4_(O_2_CCF_3_)_4_(phen)_3_]⋅phen⋅2 {(toluene)_0.73_⋅(*p*‐xylene)_0.27_} (1⋅phen⋅ tol⋅pxyl)**: A 0.05 m solution of AgO_2_CCF_3_ (92 mg, 0.40 mmol) in methanol (8 mL) was layered on to a 0.05 m solution of phenazine (72 mg, 0.40 mmol) in 4:1 (v/v) toluene:*p*‐xylene (8 mL). Yield 58 % (99 mg, 0.058 mmol); elemental analysis calcd (%): C 47.19, H 2.75, N 6.24 (for *x*=0.73); found: C 47.05, H 2.55, N 6.15.

**[Ag_4_(O_2_CCF_3_)_4_(phen)_3_]⋅phen⋅2 {(toluene)_0.49_⋅(*p*‐xylene)_0.51_} (1⋅phen⋅ tol⋅pxyl)**: A 0.05 m solution of AgO_2_CCF_3_ (92 mg, 0.40 mmol) in methanol (8 mL) was layered on to a 0.05 m solution of phenazine (72 mg, 0.40 mmol) in 6:4 (v/v) toluene:*p*‐xylene (8 mL). Yield 59 % (100 mg, 0.059 mmol): elemental analysis calcd (%): C 47.31, H 2.80, N 6.22 (for *x*=0.49); found: C 47.17, H 2.63, N 6.18.

**[Ag_4_(O_2_CCF_3_)_4_(phen)_3_]⋅phen⋅2 {(toluene)_0.38_⋅(*p*‐xylene)_0.62_} (1⋅phen⋅ tol⋅pxyl)**: A 0.05 m solution of AgO_2_CCF_3_ (92 mg, 0.40 mmol) in methanol (8 mL) was layered on to a 0.05 m solution of phenazine (72 mg, 0.40 mmol) in 1:1 (v/v) toluene:*p*‐xylene (8 mL). Yield 65 % (110 mg, 0.065 mmol); elemental analysis calcd (%): C 47.38, H 2.82, N 6.20 (for *x*=0.38); found: C 46.98, H 2.63, N 6.12.

**[Ag_4_(O_2_CCF_3_)_4_(phen)_3_]⋅phen⋅2 {(toluene)_0.30_⋅(*p*‐xylene)_0.70_} (1⋅phen⋅ tol⋅pxyl)**: A 0.05 m solution of AgO_2_CCF_3_ (92 mg, 0.40 mmol) in methanol (8 mL) was layered on to a 0.05 m solution of phenazine (72 mg, 0.40 mmol) in 4:6 (v/v) toluene:*p*‐xylene (8 mL). Yield 61 % (103 mg, 0.061 mmol); elemental analysis calcd (%): C 47.43, H 2.83, N 6.20 (for *x*=0.30); found: C 47.25, H 2.54, N 6.16.

**[Ag_4_(O_2_CCF_3_)_4_(phen)_3_]⋅phen⋅2 {(toluene)_0.14_⋅(*p*‐xylene)_0.86_} (1⋅phen⋅ tol⋅pxyl)**: A 0.05 m solution of AgO_2_CCF_3_ (92 mg, 0.40 mmol) in methanol (8 mL) was layered on to a 0.05 m solution of phenazine (72 mg, 0.40 mmol) in 1:4 (v/v) toluene:*p*‐xylene (8 mL). Yield 69 % (117 mg, 0.069 mmol); elemental analysis calcd (%): C 47.52, H 2.86, N 6.18 (for *x*=0.14); found: C 47.41, H 2.61, N 6.11.

**[Ag_4_(O_2_CCF_3_)_4_(phen)_3_]⋅phen⋅2 {(toluene)_0.46_⋅(C_6_H_6_)_0.54_} (1⋅phen⋅tol⋅ benz)**: A 0.05 m solution of AgO_2_CCF_3_ (92 mg, 0.40 mmol) in methanol (8 mL) was layered on to a 0.05 m solution of phenazine (72 mg, 0.40 mmol) in 1:1 (v/v) toluene:benzene (8 mL). Yield 56 % (95 mg, 0.056 mmol); elemental analysis calcd (%): C 46.68, H 2.61, N 6.32 (for *x*=0.46); found: C 46.54, H 2.33, N 6.63.

**[Ag_4_(O_2_CCF_3_)_4_(phen)_3_]⋅phen⋅2 {(*p*‐xylene)_0.58_⋅(C_6_H_6_)_0.42_} (1⋅phen⋅ pxyl⋅benz)**: A 0.05 m solution of AgO_2_CCF_3_ (92 mg, 0.40 mmol) in methanol (8 mL) was layered on to a 0.05 m solution of phenazine (72 mg, 0.40 mmol) in 1:1 (v/v) *p*‐xylene:benzene (8 mL). Yield 64 % (108 mg, 0.064 mmol); elemental analysis calcd (%): C 47.10, H 2.73, N, 6.25 (for *x*=0.58); found: C 46.85, H 2.42, N, 6.15.

**[Ag_4_(O_2_CCF_3_)_4_(phen)_3_]⋅phen⋅2 {(C_6_H_6_)_0.81_⋅(*o*‐xylene)_0.19_} (1⋅phen⋅ C_6_H_6_⋅oxyl)**: A 0.05 m solution of AgO_2_CCF_3_ (92 mg, 0.40 mmol) in methanol (8 mL) was layered on to a 0.05 m solution of phenazine (72 mg, 0.40 mmol) in 1:1 (v/v) benzene:*o*‐xylene (8 mL). Yield 40 % (67 mg, 0.040 mmol).

**[Ag_4_(O_2_CCF_3_)_4_(phen)_3_]⋅phen⋅2 {(C_6_H_6_)_0.91_⋅(*m*‐xylene)_0.09_} (1⋅phen⋅C_6_H_6_⋅mxyl)**: A 0.05 m solution of AgO_2_CCF_3_ (92 mg, 0.40 mmol) in methanol (8 mL) was layered on to a 0.05 m solution of phenazine (72 mg, 0.40 mmol) in 1:1 (v/v) benzene:*m*‐xylene (8 mL) . Yield 40 % (68 mg, 0.040 mmol).

**[Ag_4_(O_2_CCF_3_)_4_(phen)_3_]⋅phen⋅2 {(toluene)_0.84_⋅(*o*‐xylene)_0.16_} (1⋅phen⋅ tol⋅oxyl)**: A 0.05 m solution of AgO_2_CCF_3_ (92 mg, 0.40 mmol) in methanol (8 mL) was layered on to a 0.05 m solution of phenazine (72 mg, 0.40 mmol) in 1:1 (v/v) toluene:*o*‐xylene (8 mL). Yield 33 % (56 mg, 0.033 mmol).

**[Ag_4_(O_2_CCF_3_)_4_(phen)_3_]⋅phen⋅2 {(toluene)_0.91_⋅(*m*‐xylene)_0.09_} (1⋅phen⋅tol⋅mxyl)**: A 0.05 m solution of AgO_2_CCF_3_ (92 mg, 0.40 mmol) in methanol (8 mL) was layered on to a 0.05 m solution of phenazine (72 mg, 0.40 mmol) in 1:1 (v/v) toluene:*m*‐xylene (8 mL). Yield 32 % (55 mg, 0.032 mmol).

**[Ag_4_(O_2_CCF_3_)_4_(phen)_3_]⋅phen⋅2 {(*p*‐xylene)_0.90_⋅(*o*‐xylene)_0.10_} (1⋅phen⋅pxyl⋅oxyl)**: A 0.05 m solution of AgO_2_CCF_3_ (92 mg, 0.40 mmol) in methanol (8 mL) was layered on to a 0.05 m solution of phenazine (72 mg, 0.40 mmol) in 1:1 (v/v) *p*‐xylene:*o*‐xylene (8 mL). Yield 42 % (72 mg, 0.042 mmol).

**[Ag_4_(O_2_CCF_3_)_4_(phen)_3_]⋅phen⋅2 {(*p*‐xylene)_0.96_⋅(*m*‐xylene)_0.04_} (1⋅phen⋅pxyl⋅mxyl)**: A 0.05 m solution of AgO_2_CCF_3_ (92 mg, 0.40 mmol) in methanol (8 mL) was layered on to a 0.05 m solution of phenazine (72 mg, 0.40 mmol) in 1:1 (v/v) *p*‐xylene:*m*‐xylene (8 mL). Yield 45 % (77 mg, 0.045 mmol).

**[Ag_2_(O_2_CCF_3_)_2_(phen)] (2)**: A 0.05 m solution of AgO_2_CCF_3_ (92 mg, 0.40 mmol) in methanol (8 mL) was layered on to a 0.05 m solution of phenazine (72 mg, 0.40 mmol) in *o*‐xylene (8 mL). Yield 60 % (75 mg, 0.12 mmol); elemental analysis calcd (%): C 30.90, H 1.30, N 4.50; found: C 30.93, H 0.76, N 4.40. Compound **2** can also be synthesised by using *m*‐xylene or nitrobenzene as the solvent in place of *o*‐xylene. Alternatively, slow evaporation of a 0.05 m solution of silver trifluoroacetate and phenazine in either acetone or tetrahydrofuran (16 mL), or the layering of a 0.05 m solution of silver trifluoroacetate in ethanol (8 mL) onto a 0.05 m solution of phenazine in dichloromethane (8 mL) yields **2**.

### Analytical techniques

**X‐ray crystallography**: Single‐crystal X‐ray diffraction data were collected at 100 K for all compounds on Bruker APEX‐2 diffractometers, using Mo‐K_α_ radiation. Data were corrected for absorption using empirical methods (SADABS), based on symmetry‐equivalent reflections combined with measurements at different azimuthal angles.^25^, ^26^ Crystal structures were solved and refined against all *F* ^2^ values, using the SHELXTL program suite,^27^ or using Olex2.^28^ Non‐hydrogen atoms were refined with anisotropic displacement parameters. Hydrogen atoms were placed in calculated positions and refined using idealised geometries (riding model) and assigned fixed isotropic displacement parameters. Disorder in of the CF_3_ groups compound **1⋅**phen**⋅**tol was modelled with two orientations related by rotation. The crystal structure of **1⋅**phen**⋅**pxyl is reported in the triclinic reduced cell rather than the *C*‐centred monoclinic call analogous to those of **1⋅**phen**⋅**tol and **1⋅**phen**⋅**benz. Although the data could be indexed to the monoclinic cell, successful structure solution or satisfactory structure refinement could not be achieved. Powder diffraction data for **1⋅**phen**⋅**pxyl could also be indexed to the monoclinic cell and a limited Rietveld refinement^29^ based upon a structure model generated from the related **1⋅**phen**⋅** tol structure suggested that the structure probably does conform to the monoclinic cell (see Supporting Information). The crystal structures of the mixed‐arene‐guest materials were determined, but are not reported. These determinations were sufficient to establish them as isostructural with the single‐arene‐guest materials, but did not permit the quantity of the minor‐component arene guests to be reliably established crystallographically. Crystal data for compounds **1⋅**phen**⋅**tol, **1⋅**phen**⋅**pxyl, **1⋅**phen**⋅**benz and **2** are summarised in Table 3.

**Table 3:** Data collection, structure solution and refinement parameters for crystal structures of **1⋅**phen**⋅**tol, **1⋅**phen**⋅**pxyl, **1⋅**phen**⋅**benz and **2**.

	**1⋅**phen**⋅**tol	**1⋅**phen**⋅**pxyl	**1⋅**phen**⋅**benz	**2**
crystal habitat	plate	plate	block	plate
crystal colour	yellow	yellow	yellow	yellow
crystal size [mm]	0.81×0.36×0.02	0.33×0.21×0.06	0.25×0.25×0.22	0.34×0.17×0.05
crystal system	monoclinic	triclinic	monoclinic	monoclinic
space group, *Z*	*C*2/*c*, 4	*P* $\bar{1}$ , 2	*C*2/*c*, 4	*C*2/*c*
*a* [Å]	30.643(4)	10.2832(4)	30.7456(10)	24.2847(12)
*b* [Å]	10.136(1)	16.0990(6)	10.0068(3)	5.8277(3)
*c* [Å]	25.742(3)	21.615(1)	25.7289(8)	16.1950(8)
*α* [°]	90	85.176(3)	90	90
*β* [°]	126.031(3)	76.719(3)	125.7621(14)	131.145(2)
*γ* [°]	90	71.424(3)	90	90
*V* [Å^3^]	6465.9(14)	3301.0(2)	6423.3(4)	1725.97(15)
*ρ*_calcd_ [Mg m^−3^]	1.837	1.827	1.823	2.394
*T* [K]	100	100	100	100
*μ*_(Mo‐Ka)_ [mm^−1^]	1.294	1.268	1.301	2.361
*θ* range [°]	1.96 to 27.69	1.936 to 27.572	2.599 to 26.817	2.23 to 27.66
reflns collected	52233	40734	54203	11422
independent reflns (*R*_int_)	7451 (0.0793)	14157 (0.0509)	6880 (0.0574)	1997 (0.0195)
reflns used in refinement, *n*	7451	14157	6880	1997
LS parameters, *p*	536	581	451	137
restraints, *r*	24	0	0	0
*R*1 (*F*)^[a],^ [*I*>2.0σ(*I*)]	0.0566	0.1195	0.0406	0.0165
*wR*2 (*F* ^*2*^)^[a]^, all data	0.1508	0.3123	0.656	0.0418
*S*(*F* ^*2*^)^[a]^, all data	0.960	1.159	1.035	1.068

[a] *R*1(*F*)=Σ(|*F*_o_|−|*F*_c_|)/Σ|*F_o_*|; *wR*2(*F* ^2^)=[Σ*w*(*F*_o_^2^−*F*_c_^2^)^2^/Σ*wF*_o_^4^]^1/2^; *S*(*F* ^2^)=[Σ*w*(*F*_o_^2^−*F*_c_^2^)^2^/(*n+r*−*p*)]^1/2^.

CCDC 14575 (**1⋅**phen**⋅**tol), 14576 (**1⋅**phen**⋅**pxyl), 14578 (**1⋅**phen**⋅**benz), and 14577 **2** contain the supplementary crystallographic data. These data can be obtained free of charge by The Cambridge Crystallographic Data Centre.

**Powder X‐ray diffraction**: Samples prepared as described above were loaded into borosilicate capillaries of diameter 0.7 mm. Data were collected on beamline I11,^30^, ^31^ at Diamond Light Source (X‐ray wavelengths given in Supporting Information). Data were collected using a wide‐angle (90°) PSD detector comprised of 18 Mythen‐2 modules. Scans were collected in pairs with a 0.25° 2*θ* offset (to account for the gaps between the Mythen‐2 modules). These pairs of scans were then summed. A series of such scans amounting to a total of 52 s of exposure time was conducted and summed for each sample. Diffraction patterns were indexed and fitted using the TOPAS Academic program,^32^ by Pawley refinement^33^ for data with *d*_min_≤1.18 Å in each case, using starting models from previous single crystal structure determinations. Full details of refinements and all fitted patterns are included in Supporting Information.

**Elemental analysis**: Elemental analyses were carried out by the University of Sheffield Department of Chemistry elemental analysis service, using a PerkinElmer 2400 CHNS/O Series II Elemental Analyser. Elemental analyses were conducted immediately upon removal of the crystals from the mother liquor, to prevent loss of the arene solvent contained.

^**1**^ **H NMR spectroscopy**: Analytes were air‐dried for precisely five minutes, and split into two equal portions (thus giving two measurements) and dissolved in [D_6_]DMSO, then filtered through cotton wool. ^1^H NMR spectra were measured on a Bruker AV 400 MHz spectrometer. The NMR spectra can be found in the Supporting Information, Section 4. The NMR spectra were analysed using the Bruker TOPSPIN 3.1 programme. Methyl peaks for mixed xylene systems, which did not show complete baseline separation, were deconvoluted using the mixed‐line descriptor (mixed Lawrencian & Gaussian) deconvolution function in TOPSPIN.

**Gas chromatography**: The solutions used for ^1^H NMR were transferred to glass vials using crimped caps, and then analysed using a PerkinElmer Autosystem GC with an Alltech^TM^ Heliflex^TM^ AT‐1 capillary column (L×I.D. 30 m×0.32 mm×d_f_ 5.00 μm), heating from 40 to 200 °C at 10 °C min^−1^. Expected guest retention times were found to be 9.9 min (benzene), 12.7 min (toluene), 15.1 min (*p*‐xylene), 15.2 min (*m*‐xylene‐indistinguishable from *p*‐xylene) and 15.7 min (*o*‐xylene). Relative content of guests was determined by direct comparison of chromatogram peak areas. The gas chromatograms can be found in the Supporting Information.

## Supporting information

As a service to our authors and readers, this journal provides supporting information supplied by the authors. Such materials are peer reviewed and may be re‐organized for online delivery, but are not copy‐edited or typeset. Technical support issues arising from supporting information (other than missing files) should be addressed to the authors.

Supplementary
