# Supplementary material for: Arene Selectivity by a Flexible Coordination Polymer Host
Source: Chemistry. 2016 Aug 2;22(37):13120–6. doi: 10.1002/chem.201601870 (PMC5096259; doi:10.1002/chem.201601870)
Supplement: Supplementary file 1 — Supplementary [file CHEM-22-13120-s001.pdf]

CHEMISTRY
A European Journal

Supporting Information

Arene Selectivity by a Flexible Coordination Polymer Host

James S. Wright,[a] Iñigo J. Vitórica-Yrezábal,[a, b] Stephen P. Thompson,[c] and Lee Brammer*[a]

chem_201601870_sm_miscellaneous_information.pdf

Supporting Information

Arene selectivity by a flexible coordination polymer host

James S. Wright,[a] Iñigo J. Vitórica-Yrezábal,[a,b] Stephen P. Thompson[c] and Lee Brammer*[a]

[a] *Dr. J. S. Wright, Dr. I. J. Vitórica-Yrezábal, Prof. L. Brammer*

Department of Chemistry, University of Sheffield, Brook Hill, Sheffield S3 7HF (UK).

E-mail: lee.brammer@sheffield.ac.uk

[b] *Dr. I. J. Vitórica-Yrezábal*

Current address:

School of Chemistry, University of Manchester, Oxford Road, Manchester M13 9PL (UK).

[c] *Dr. S. P. Thompson*

Diamond Light Source, Harwell Science and Innovation Campus, Didcot, Oxfordshire OX11 0DE (UK).

Table of Contents

1. Phase purity checks by XRPD, pure arene guest or solvent-free systems Page S2

2. Structure solution / Rietveld refinement, **1.phen.pxyl** Page S4

3. Phase purity checks by XRPD, mixed arene investigations Page S5

4. ^{1}H-NMR spectroscopic analysis of mixed arene investigations Page S15

5. Gas chromatographic analysis of mixed arene investigations Page S34

6. Selectivity constant calculations Page S44

7. References Page S44

1. Phase-purity checks by XRPD for 1.phen.arene and 2

In all cases, the yellow microcrystalline product was loaded into a 0.7 mm borosilicate capillary. X-ray diffraction data were collected (wavelength noted in each case) at beamline I11 at Diamond Light Source,[S1,S2] equipped with a wide angle (90 °) PSD detector comprising 18 Mythen-2 modules. A pair of scans was conducted at room temperature, related by a 0.25 ° detector offset to account for gaps between detector modules. Five such scan pairs (ten seconds exposure) were collected, preceded and followed by a one-second scan to check for beam damage (total exposure 52 seconds). The resulting patterns were summed to give the final pattern for structural analysis. All data were collected at room temperature. The powder patterns were indexed using the TOPAS program.[S3]

Phase-purity check, 1.phen.tol. (λ = 0.826136(2) Å)

The pattern was compared with calculated X-ray powder patterns for **1.phen.tol** already established from single-crystal X-ray diffraction. The unit cell parameters of **1.phen.tol** were used as a starting point for Pawley refinement,[S4] employing 2253 parameters (8 background, 1 zero error, 5 profile, 4 cell, 2235 reflections). Pawley refinement converged to R_{wp} = 6.76, $R_{wp'}$ = 13.31. [a = 30.7369(8) Å, b = 10.3716(4) Å, c = 26.1040(6) Å, β = 125.807(1)°, V = 6748.8(4) Å^3].

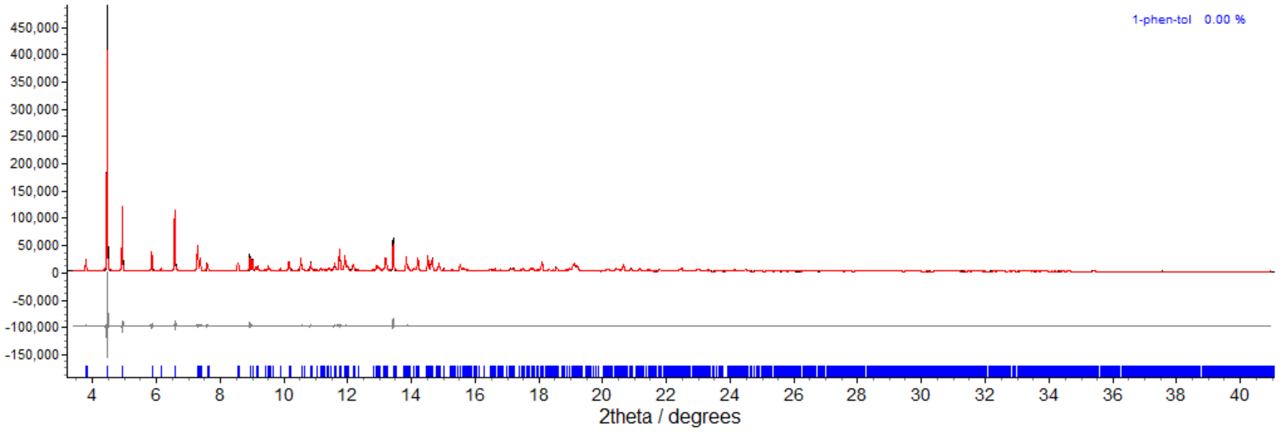

Figure S1. Observed (black) and calculated (red) profiles and difference plot [I_{obs}-I_{calc}] (grey) of the Pawley refinement. (2θ range 3.40 – 41.0 °, d_{min} = 1.18 Å).

Phase-purity check, 1.phen.C_6H_6. (λ = 0.82562(1) Å)

The pattern was compared with calculated X-ray powder patterns for **1.phen.C_6H_6** already established from single-crystal X-ray diffraction. The unit cell parameters of **1.phen.C_6H_6** were used as a starting point for Pawley refinement,[S4] employing 2406 parameters (6 background, 1 zero error, 5 profile, 4 cell, 2390 reflections). Pawley refinement converged to R_{wp} = 11.12, $R_{wp'}$ = 12.43. [a = 30.814(2) Å, b = 10.2490(8) Å, c = 26.009(1) Å, β = 125.474(3)°, V = 6689.3(8) Å^3].

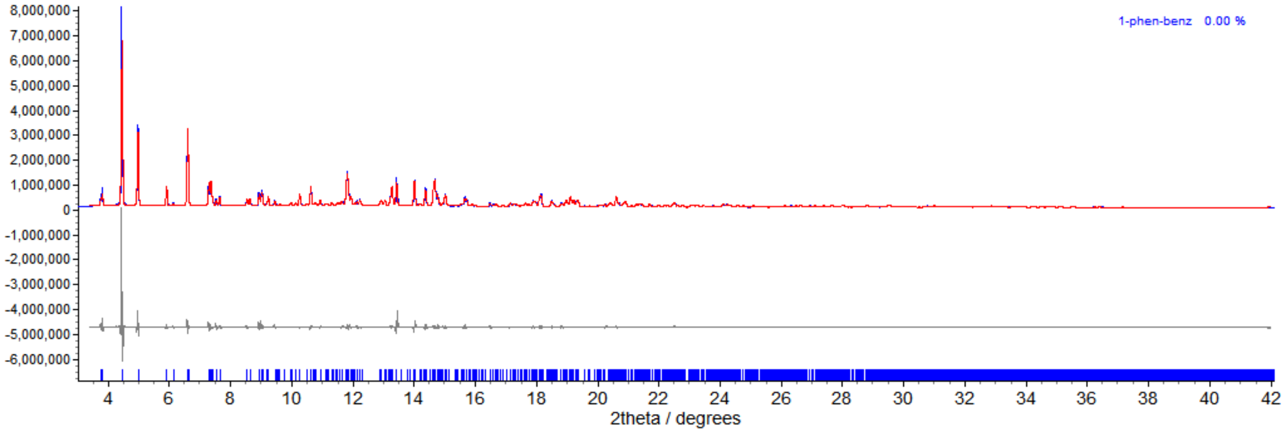

Figure S2. Observed (blue) and calculated (red) profiles and difference plot [I_{obs}-I_{calc}] (grey) of the Pawley refinement. (2θ range 3.40 – 42.0 °, d_{min} = 1.01 Å).

Phase-purity check, 2. (λ = 0.82562(1) Å)

The pattern was compared with calculated X-ray powder patterns for **2** already established from single-crystal X-ray diffraction. The unit cell parameters of **2** were used as a starting point for Pawley refinement,[S4] employing 1557 parameters (9 background, 1 zero error, 5 profile, 4 cell, 1538 reflections). Pawley refinement converged to R_{wp} = 5.29, $R_{wp'}$ = 12.33. [a = 24.461(2) Å, b = 5.71760(5) Å, c = 16.9103(1) Å, β = 131.4731(5)°, V = 1772.28(3) Å^3].

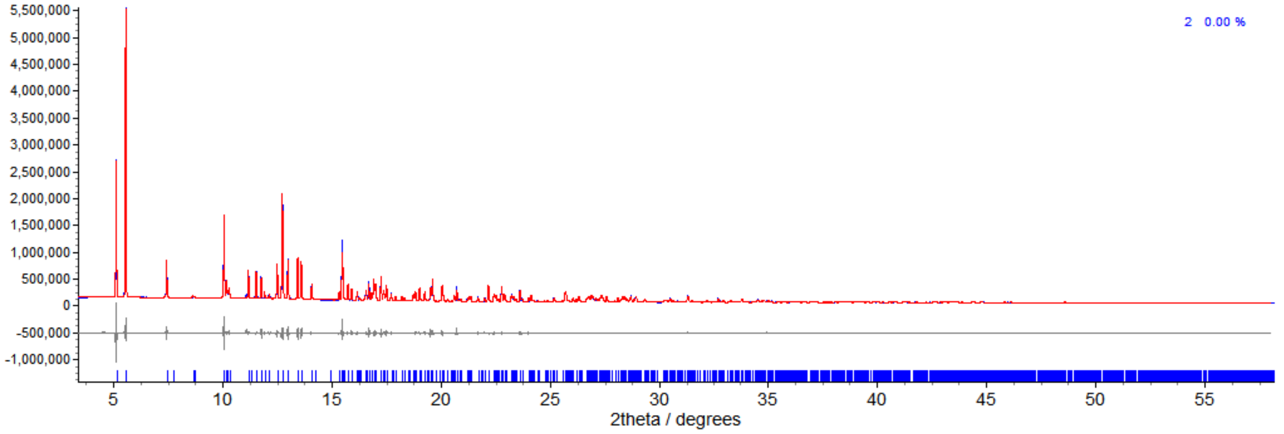

Figure S3. Observed (blue) and calculated (red) profiles and difference plot [I_{obs}-I_{calc}] (grey) of the Pawley refinement. (2θ range 3.40 – 58.0 °, d_{min} = 0.85 Å).

2. Structure solution / Rietveld refinement, 1.phen.pxyl

The data were collected as described in section 1 (λ = 0.82562(1) Å). The pattern was compared with calculated X-ray powder patterns for the known phases **1.phen.tol** and **1.phen.C_6H_6**, already established from single-crystal X-ray diffraction. The unit cell parameters of **1.phen.pxyl** as determined from the orientation matrix determination on single crystals were used as a starting point for Pawley refinement,[S4] employing 2242 parameters (6 background, 1 zero error, 5 profile, 4 cell, 2226 reflections). Pawley refinement converged to R_{wp} = 7.90, $R_{wp'}$ = 15.75. [a = 30.807(2) Å, b = 10.558(1) Å, c = 26.231(1) Å, β = 125.702(3)°, V = 6929(1) $Å^3$]. The starting model used for the Rietveld refinement,[S5] conducted using TOPAS, was a structure for **1.phen.pxyl** built in Accelrys Materials Studio 6.0, using the structure of **1.phen.tol** as a starting point but replacing toluene by *p*-xylene in the same location). Adjustment of the *p*-xylene position and orientation by simulated annealing was attempted, but was unsuccessful. Therefore refinement was conducted with fixed atomic coordinates and employed 21 parameters (6 background, 1 zero error, 5 profile, 4 cell, 1 scale, 3 thermal parameters to describe the silver ions, coordination framework and *p*-xylene guest, 1 preferred orientation correction in the 00X direction). Rietveld refinement converged to R_{wp} = 17.53, $R_{wp'}$ = 38.35. [**1.phen.pxyl:** a = 30.758(2) Å, b = 10.5183(5) Å, c = 26.238(2) Å, β = 126.082(5)°, V = 6860.2(8) $Å^3$].

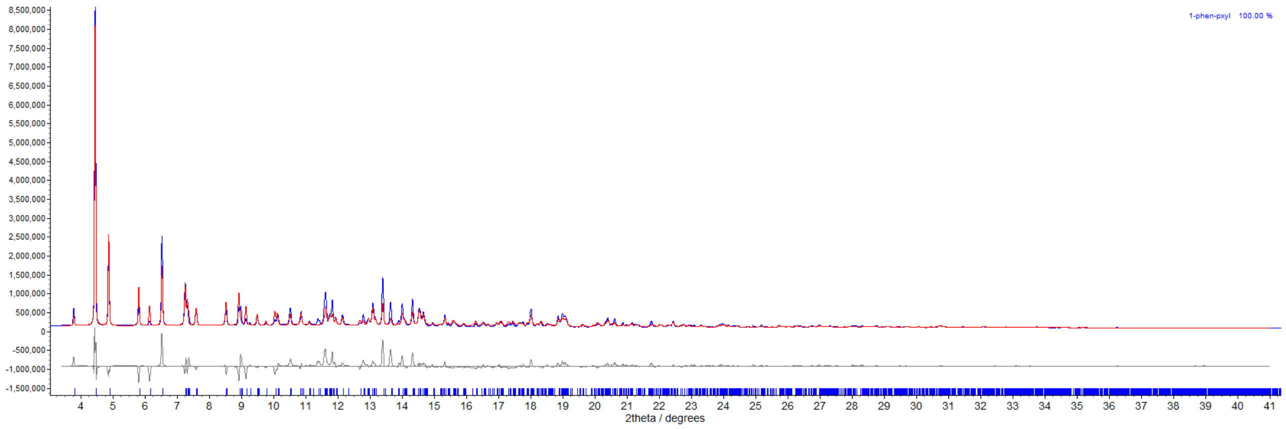

Figure S4. Observed (blue) and calculated (red) profiles and difference plot [I_{obs}-I_{calc}] (grey) of the Rietveld refinement. (2θ range 3.40 – 41.0 °, d_{min} = 1.18 Å).

3. Phase-purity checks by XRPD for 1.phen.arene compounds with mixed arene prepared from mixed arene solutions.

In all cases, the yellow microcrystalline product was loaded into a 0.7 mm borosilicate capillary. X-ray diffraction data were collected at room temperature at beamline I11, Diamond Light Source,[S1,S2] which is equipped with a wide angle (90 °) PSD detector comprising 18 Mythen-2 modules. A pair of scans was conducted, each related by a 0.25 ° detector offset to account for gaps between detector modules. Five such scan pairs (10 s exposure) were collected, preceded and followed by a 1 s scan to check for beam damage (total exposure 52 s). The resulting patterns were summed to give the final pattern for structural analysis. The powder patterns were indexed using the TOPAS program.[S3]

3.1. Mixed toluene / p-xylene systems

In all cases, inspection of the X-ray powder diffraction patterns indicated the presence of a small amount of the solvent-free crystalline phase **2**. This was therefore included along with the major phase in the Pawley refinement. The peaks corresponding to the arene-free phase **2** are indicated by blue tickmarks in all diagrams below, demonstrating their low intensity and therefore likely small relative content. Rietveld refinement was not feasible for these materials.

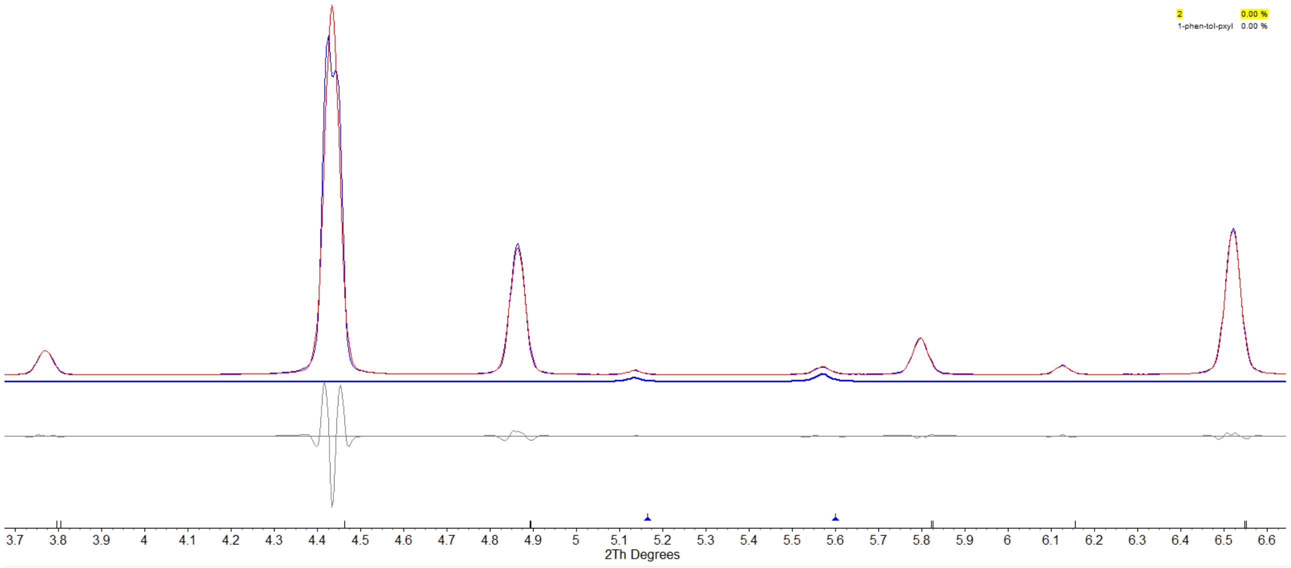

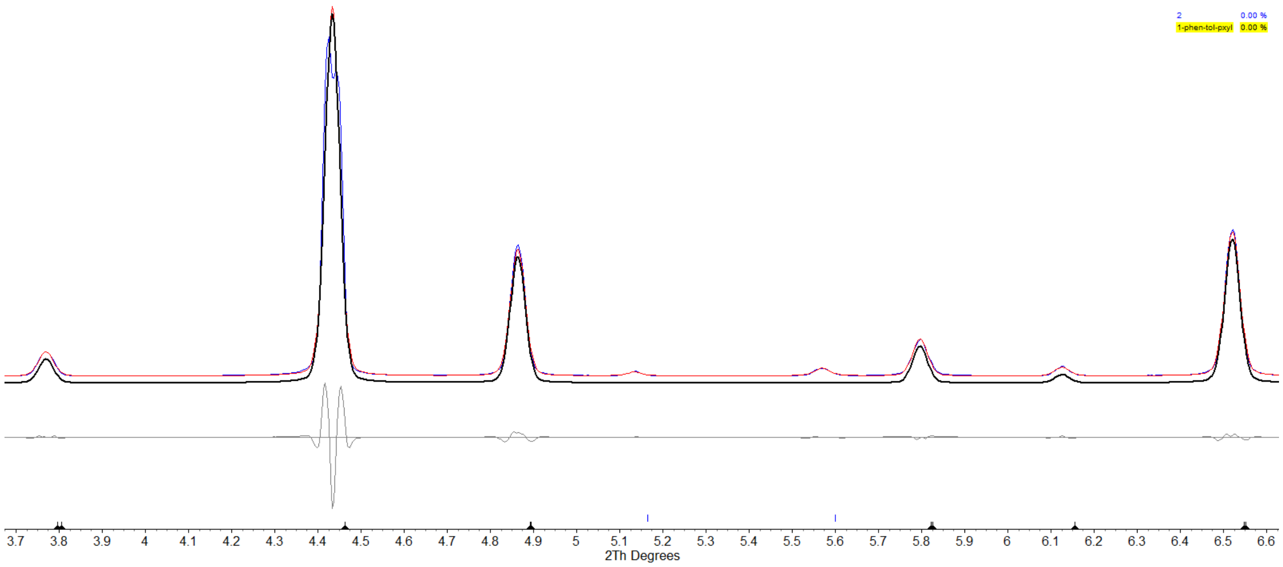

Figure S5. Demonstration of the presence and relative intensities of reflections using low-angle data, corresponding to the phases **1.phen.arene** and **2**, in this example shown for **1.phen.tol$_x$.pxyl$_{(1-x)}$ (x = 0.73 by NMR/GC)**. Calculated (red) profile is the result of Pawley refinement, and the difference plot [I_{obs}-I_{calc}] is shown in grey. ***Above:*** Overlay in blue showing reflections consistent with phase **2** and ***below:*** overlay in black showing reflections consistent with the appropriate **1.phen.arene** phase.

Phase-purity check, 1.phen.tol$_x$.pxyl$_{(1-x)}$ (x = 0.73 average by NMR/GC).

(λ = 0.82562(1) Å)

The pattern was compared with calculated X-ray powder patterns for **1.phen.tol**, **1.phen.pxyl** and **2**, already established from single-crystal X-ray diffraction. The unit cell parameters of **1.phen.tol** and **2** were used as a starting point for Pawley refinement,[S4] employing 3081 parameters (7 background, 1 zero error, 9 profile, 8 cell, 3056 reflections). Pawley refinement converged to R_{wp} = 7.59, $R_{wp'}$ = 12.22. [phase "**1.phen.tol$_x$.pxyl$_{(1-x)}$**": a = 30.805(3) Å, b = 10.437(1) Å, c = 26.206(1) Å, β = 125.752(4)°, V = 6837(1) Å^3; phase "**2**": a = 24.584(6) Å, b = 5.733(2) Å, c = 16.979(2) Å, β = 131.56(2)°, V = 1790.6(9) Å^3].

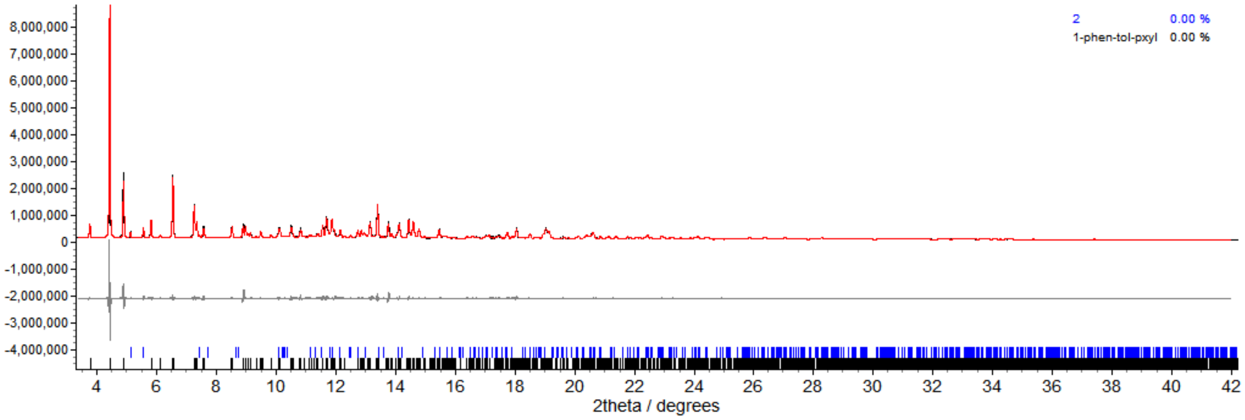

Figure S6. Observed (blue) and calculated (red) profiles and difference plot [I_{obs}-I_{calc}] (grey) of the Pawley refinement. (2θ range 3.40 – 42.0 °, d_{min} = 1.15 Å).

Phase-purity check, 1.phen.tol$_x$.pxyl$_{(1-x)}$ (x = 0.49 average by NMR/GC).

(λ = 0.82562(1) Å)

The pattern was compared with calculated X-ray powder patterns for **1.phen.tol**, **1.phen.pxyl** and **2** already established from single-crystal X-ray diffraction. The unit cell parameters of **1-phen-tol** and **2** were used as a starting point for Pawley refinement,[S4] employing 3082 parameters (7 background, 1 zero error, 9 profile, 8 cell, 3057 reflections). Pawley refinement converged to R_{wp} = 5.25, $R_{wp'}$ = 13.89. [phase "**1.phen.tol$_x$.pxyl$_{(1-x)}$**": a = 30.825(3) Å, b = 10.466(1) Å, c = 26.220(1) Å, β = 125.781(4)°, V = 6862 (1) Å^3; phase "**2**": a = 24.700(2) Å, b = 5.692(6) Å, c = 16.961(5) Å, β = 131.83(3)°, V = 1776(2) Å^3].

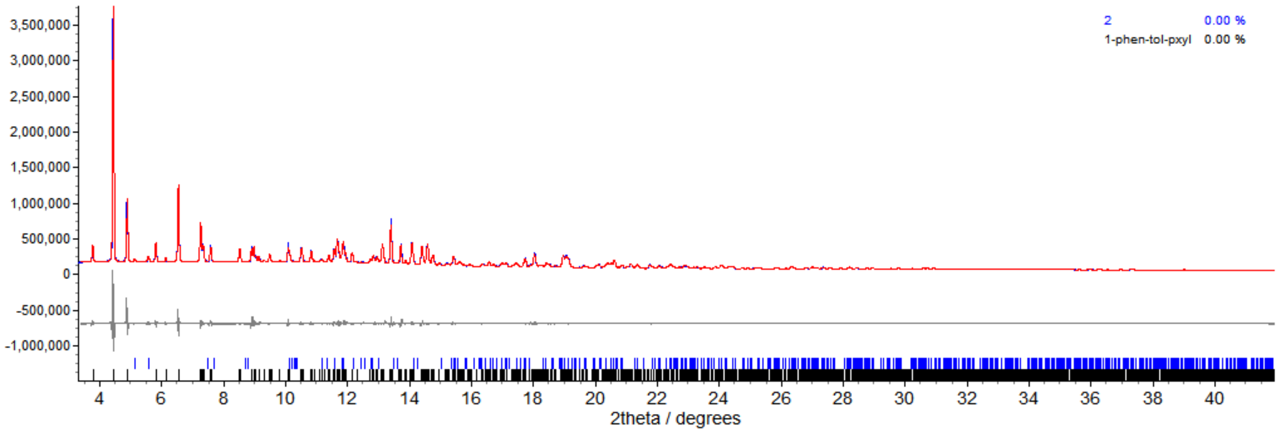

Figure S7. Observed (blue) and calculated (red) profiles and difference plot [I$_{obs}$-I$_{calc}$] (grey) of the Pawley refinement. (2θ range 3.40 – 42.0 °, d_{min} = 1.15 Å).

Phase-purity check, 1.phen.tol$_x$.pxyl$_{(1-x)}$ (x = 0.38 average by NMR/GC).

(λ = 0.82562(1) Å)

The pattern was compared with calculated X-ray powder patterns for **1.phen.tol**, **1.phen.pxyl** and **2** already established from single-crystal X-ray diffraction. The unit cell parameters of **1-phen-tol** and **2** were used as a starting point for Pawley refinement,[S4] employing 3088 parameters (7 background, 1 zero error, 9 profile, 8 cell, 3063 reflections). Pawley refinement converged to R_{wp} = 9.27, $R_{wp'}$ = 16.08. [phase "**1.phen.tol$_x$.pxyl$_{(1-x)}$**": a = 30.829(3) Å, b = 10.482(1) Å, c = 26.328(1) Å, β = 126.068(3)°, V = 6877(1) Å^3; phase "**2**": a = 24.568(2) Å, b = 5.7119(9) Å, c = 16.9746(6) Å, β = 131.514(4)°, V = 1783.7(3) Å^3].

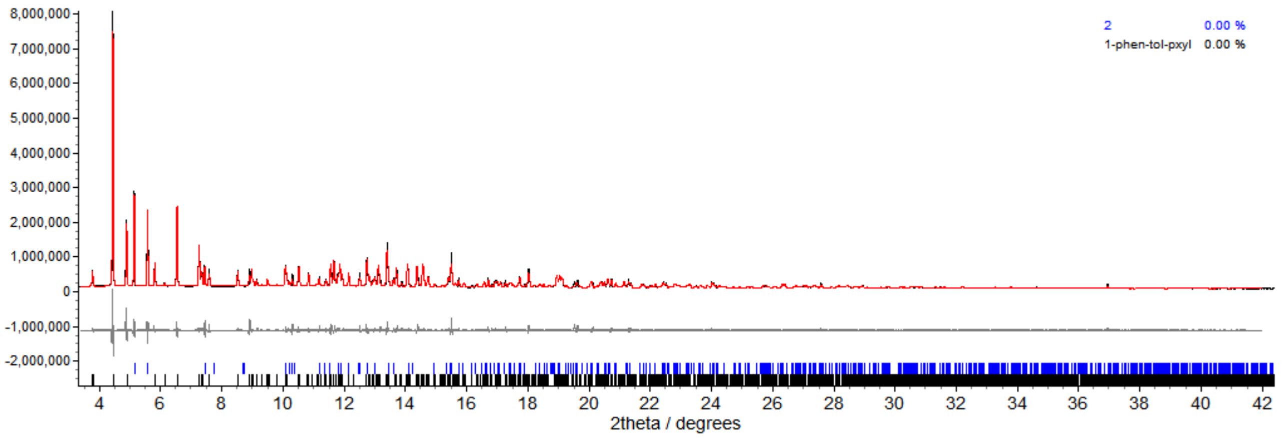

Figure S8. Observed (black) and calculated (red) profiles and difference plot [I_{obs}-I_{calc}] (grey) of the Pawley refinement. (2θ range 3.40 – 42.0 °, d_{min} = 1.15 Å).

Phase-purity check, 1.phen.tol$_x$.pxyl$_{(1-x)}$ (x = 0.30 average by NMR/GC).

(λ = 0.82562(1) Å)

The pattern was compared with calculated X-ray powder patterns for **1.phen.tol**, **1.phen.pxyl** and **2** already established from single-crystal X-ray diffraction. The unit cell parameters of **1-phen-tol** and **2** were used as a starting point for Pawley refinement,[S4] employing 3082 parameters (7 background, 1 zero error, 9 profile, 8 cell, 3057 reflections). Pawley refinement converged to R_{wp} = 6.93, $R_{wp'}$ = 14.39. [phase "**1.phen.tol$_x$.pxyl$_{(1-x)}$**": *a* = 30.8290(5) Å, *b* = 10.4924(1) Å, *c* = 26.2200(3) Å, β = 125.772(1)°, *V* = 6881.4(2) Å^3; phase "**2**": *a* = 24.5283(7) Å, *b* = 5.7202(2) Å, *c* = 16.9602(4) Å, β = 131.452(2)°, *V* = 1783.6(1) Å^3].

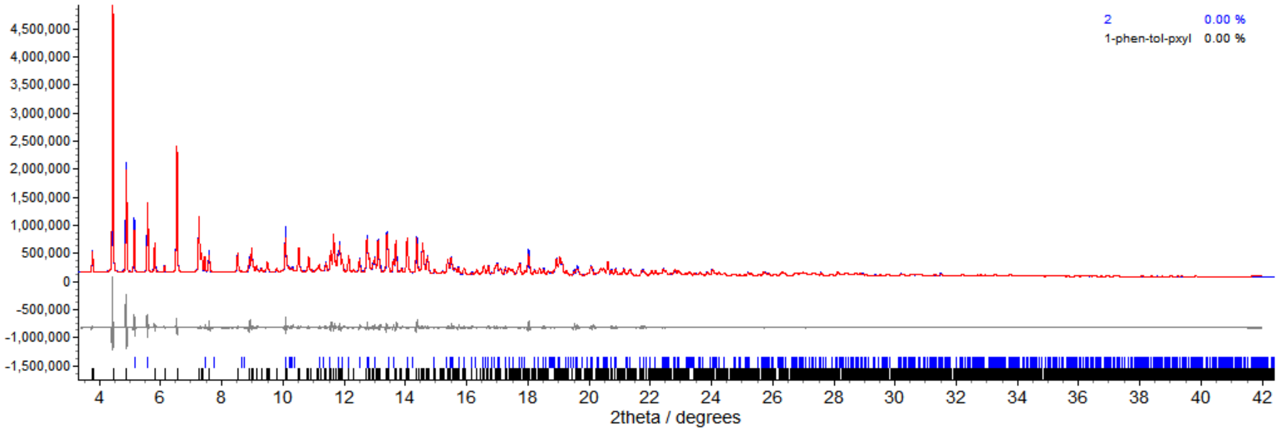

Figure S9. Observed (blue) and calculated (red) profiles and difference plot [I_{obs}-I_{calc}] (grey) of the Pawley refinement. (2θ range 3.40 – 42.0 °, d_{min} = 1.15 Å).

Phase-purity check, 1.phen.tol$_x$.pxyl$_{(1-x)}$ (x = 0.14 average by NMR/GC).

(λ = 0.82562(1) Å)

The pattern was compared with calculated X-ray powder patterns for **1.phen.tol**, **1.phen.pxyl** and **2** already established from single-crystal X-ray diffraction. The unit cell parameters of **1-phen-tol** and **2**

were used as a starting point for Pawley refinement,[S4] employing 3096 parameters (7 background, 1 zero error, 9 profile, 8 cell, 3071 reflections). Pawley refinement converged to R_{wp} = 4.46, $R_{wp'}$ = 8.68. [phase "**1.phen.tol$_x$.pxyl$_{(1-x)}$**": a = 30.7566(2) Å, b = 10.49438(6) Å, c = 26.1616(2) Å, β = 125.8664(7)°, V = 6843.1(1) Å^3; phase "**2**": a = 24.472(2) Å, b = 5.7138(7) Å, c = 16.916(1) Å, β = 131.507(6)°, V = 1771.4(3) Å^3].

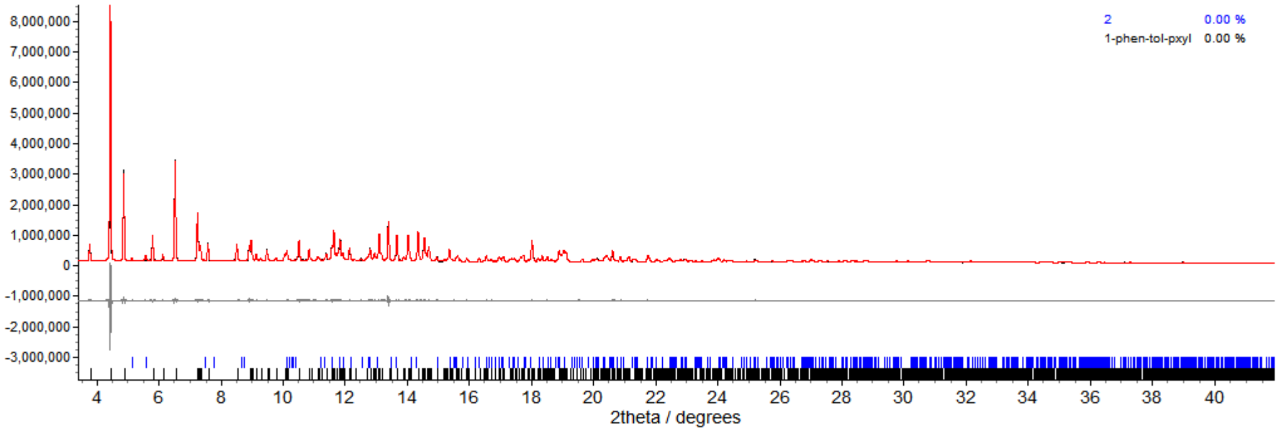

Figure S10. Observed (black) and calculated (red) profiles and difference plot [I_{obs}-I_{calc}] (grey) of the Pawley refinement. (2θ range 3.40 – 42.0 °, d_{min} = 1.15 Å).

3.2. Mixed toluene & other arenes

In all cases, visual inspection of the X-ray powder diffraction patterns indicated the presence of a small amount of the solvent-free crystalline phase **2**. This was therefore included in the Pawley refinement. The peaks corresponding to the solvent free phase **2** are indicated by blue tickmarks in all diagrams below, demonstrating their low intensity and therefore likely small relative content (see figure S5).

Inclusion of other arenes is, however, also indicated in these cases by expansion of the unit cell of the solvent-containing phase **1.phen.tol$_x$.arene$_{(1-x)}$**, compared with the room temperature unit cell of **1.phen.tol**. However, the resolution of the single-crystal or powder X-ray diffraction data is not sufficient to enable the proportion of xylene to be determined.

Phase-purity check, 1.phen.tol$_x$.oxyl$_{(1-x)}$ (x = 0.79 average by NMR/GC).

(λ = 0.82562 (1) Å)

The pattern was compared with calculated X-ray powder patterns for **1.phen.tol** and **2** already established from single-crystal X-ray diffraction. The unit cell parameters of **1-phen-tol** and **2** were used as a starting point for Pawley refinement,[S4] employing 3073 parameters (7 background, 1 zero error, 9 profile, 8 cell, 3048 reflections). Pawley refinement converged to R_{wp} = 5.96, $R_{wp'}$ = 11.54. [phase "**1.phen.tol$_x$.oxyl$_{(1-x)}$**": a = 30.8329(3) Å, b = 10.3887(1) Å, c = 26.2127(2) Å, β = 125.6919(6)°,

V = 6819.1(1) Å^3; phase "**2**": a = 24.5668(9) Å, b = 5.5933(1) Å, c = 16.9478(6) Å, β = 131.618(2)°, V = 1741.0(1) Å^3].

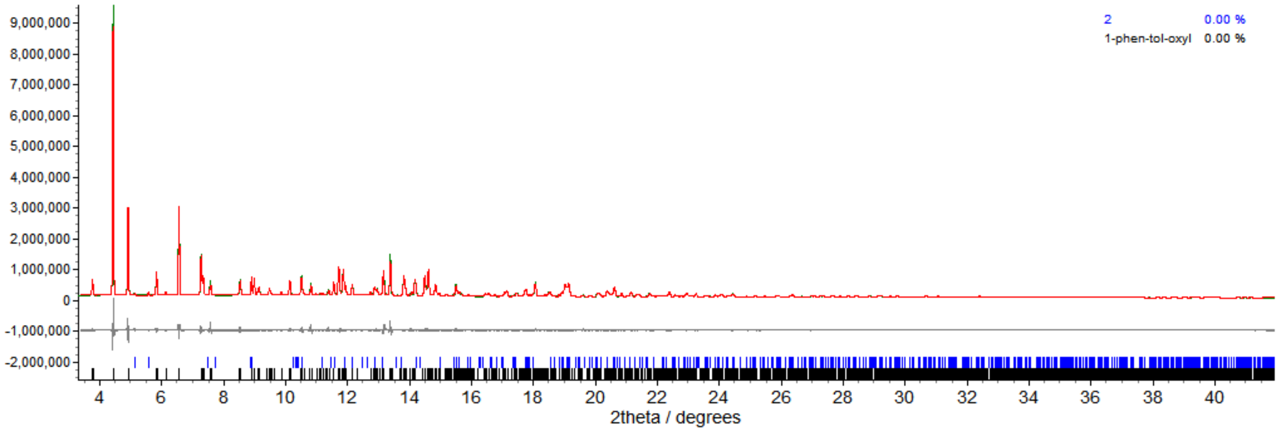

Figure S11. Observed (green) and calculated (red) profiles and difference plot [I_{obs}-I_{calc}] (grey) of the Pawley refinement. (2θ range 3.40 – 42.0 °, d_{min} = 1.15 Å).

Phase-purity check, 1.phen.tol$_x$.mxyl$_{(1-x)}$ (x = 0.87 average by NMR/GC).

(λ = 0.82562 (1) Å)

The pattern was compared with calculated X-ray powder patterns for **1.phen.tol** and **2** already established from single-crystal X-ray diffraction. The unit cell parameters of **1-phen-tol** and **2** were used as a starting point for Pawley refinement,[S4] employing 3054 parameters (7 background, 1 zero error, 9 profile, 8 cell, 3029 reflections). Pawley refinement converged to R_{wp} = 12.24, R_{wp}' = 23.35. [phase "**1.phen.tol$_x$.mxyl$_{(1-x)}$**": a = 30.7486(6) Å, b = 10.3912(1) Å, c = 26.1434(5) Å, β = 125.812(1)°, V = 6774.0(2) Å^3; phase "**2**": a = 24.482(2) Å, b = 5.5830(5) Å, c = 16.9206(8) Å, β = 131.479(5)°, V = 1732.7(3) Å^3].

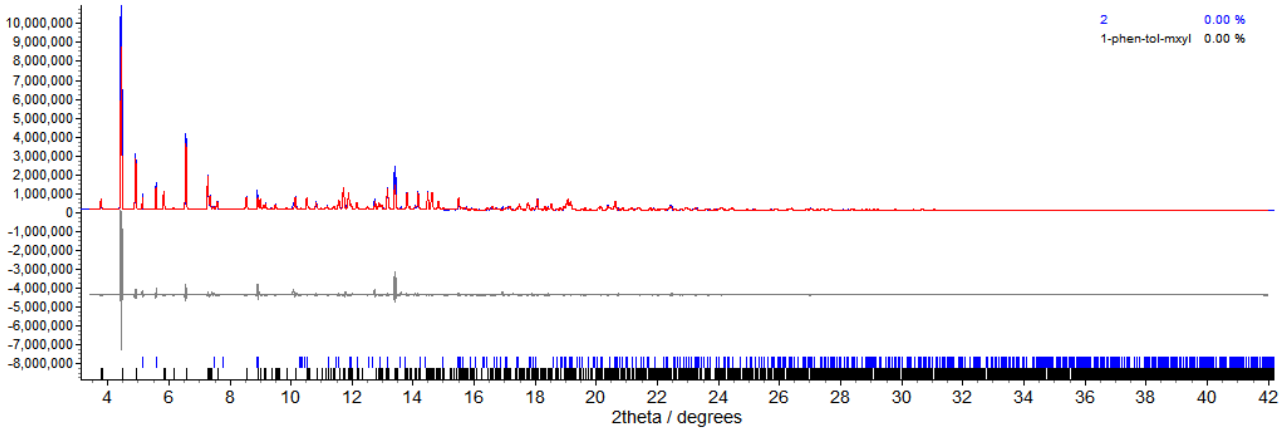

Figure S12. Observed (blue) and calculated (red) profiles and difference plot [I_{obs}-I_{calc}] (grey) of the Pawley refinement. (2θ range 3.40 – 42.0 °, d_{min} = 1.15 Å).

Phase-purity check, 1.phen.tol$_x$.$C_6H_{6(1-x)}$ (x = 0.46 average by NMR/GC).

(λ = 0.82562 (1) Å)

The pattern was compared with calculated X-ray powder patterns for **1.phen.tol** and **2** already established from single-crystal X-ray diffraction. The unit cell parameters of **1.phen-tol** and **2** were used as a starting point for Pawley refinement,[S4] employing 3054 parameters (7 background, 1 zero error, 9 profile, 8 cell, 3029 reflections). Pawley refinement converged to R_{wp} = 12.43, $R_{wp'}$ = 22.02. [phase "**1.phen.tol$_x$.$C_6H_{6(1-x)}$**": *a* = 30.8065(6) Å, *b* = 10.3180(2) Å, *c* = 26.1278(5) Å, β = 125.643(1)°, *V* = 6749.1(2) Å^3; phase "**2**": *a* = 24.6960(8) Å, *b* = 5.6087(2) Å, *c* = 16.9865(7) Å, β = 131.831(3)°, *V* = 1753.1(1) Å^3].

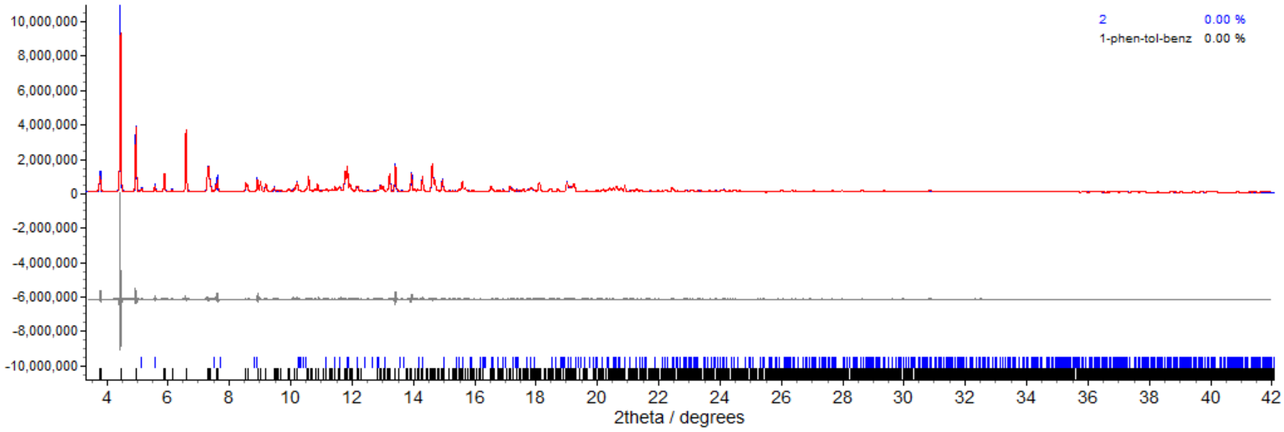

Figure S13. Observed (blue) and calculated (red) profiles and difference plot [I_{obs}-I_{calc}] (grey) of the Pawley refinement. (2θ range 3.40 – 42.0 °, d_{min} = 1.15 Å).

3.3. Mixed p-xylene / other arene systems

In all cases, visual inspection of the X-ray powder diffraction patterns indicated the presence of a small amount of the solvent-free crystalline phase **2**. This was therefore included in the Pawley refinement. The peaks corresponding to the solvent free phase **2** are indicated by blue tickmarks in all diagrams below, demonstrating their low intensity and therefore likely small relative content (see Figure S5).

Inclusion of other arenes is, however, also indicated in these cases by expansion of the unit cell of the solvent-containing phase **1.phen.tol$_x$.arene$_{(1-x)}$**, compared with the room temperature unit cell of **1.phen.pxyl**. However, the resolution of the single-crystal or powder X-ray diffraction data is not sufficient to enable the proportion of xylene to be determined.

Phase-purity check, 1.phen.pxyl$_x$.oxyl$_{(1-x)}$ (x = 0.90 average by NMR/GC).

(λ = 0.82562 (1) Å)

The pattern was compared with calculated X-ray powder patterns for **1.phen.pxyl** and **2** already established from single-crystal X-ray diffraction. The unit cell parameters of **1-phen-pxyl** and **2** were used as a starting point for Pawley refinement,[S4] employing 3112 parameters (7 background, 1 zero

error, 9 profile, 8 cell, 3087 reflections). Pawley refinement converged to R_{wp} = 8.36, $R_{wp'}$ = 18.38. [phase "**1.phen.pxyl$_x$.oxyl$_{(1-x)}$**": a = 30.8405(6) Å, b = 10.5488(2) Å, c = 26.2405 (4) Å, β = 125.718(1)°, V = 6931.1(2) Å^3; phase "**2**": a = 24.5580(9) Å, b = 5.7634(2) Å, c = 16.9723(6) Å, β = 131.497(2)°, V = 1799.2(1) Å^3].

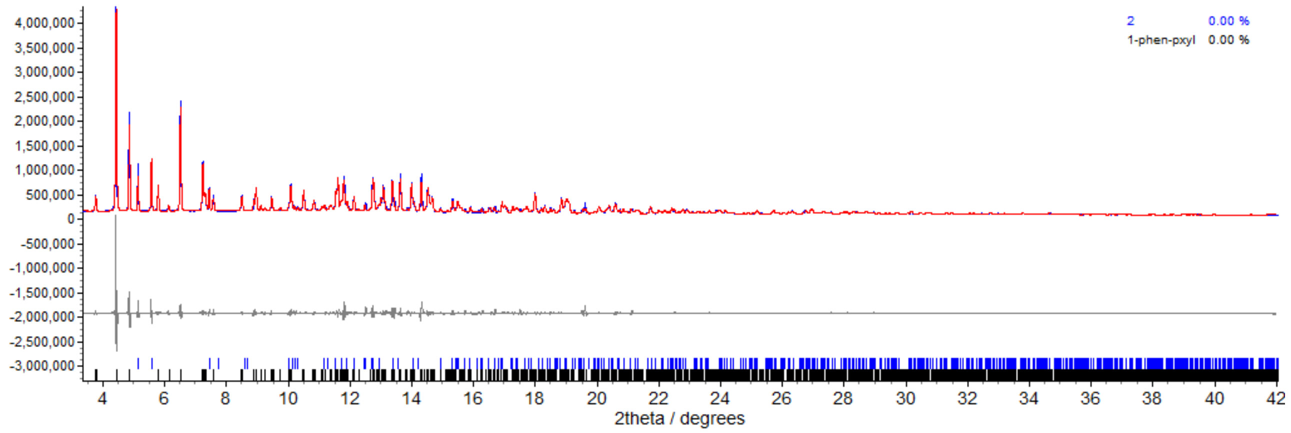

Figure S14. Observed (blue) and calculated (red) profiles and difference plot [I_{obs}-I_{calc}] (grey) of the Pawley refinement. (2θ range 3.40 – 42.0 °, d_{min} = 1.15 Å).

Phase-purity check, 1.phen.pxyl$_x$.mxyl$_{(1-x)}$ (x = 0.93 by NMR).

(λ = 0.82562 (1) Å)

The pattern was compared with calculated X-ray powder patterns for **1.phen.pxyl** and **2** already established from single-crystal X-ray diffraction. The unit cell parameters of **1-phen-pxyl** and **2** were used as a starting point for Pawley refinement,[S4] employing 3121 parameters (7 background, 1 zero error, 9 profile, 8 cell, 3096 reflections). Pawley refinement converged to R_{wp} = 13.13, $R_{wp'}$ = 26.86. [phase "**1.phen.pxyl$_x$.mxyl$_{(1-x)}$**": a = 30.7731(4) Å, b = 10.5280(1) Å, c = 26.1686(4) Å, β = 125.807(1)°, V = 6875.7(2) Å^3; phase "**2**": a = 24.500(1) Å, b = 5.7306(4) Å, c = 16.927(1) Å, β = 131.408(4)°, V = 1782.4(2) Å^3].

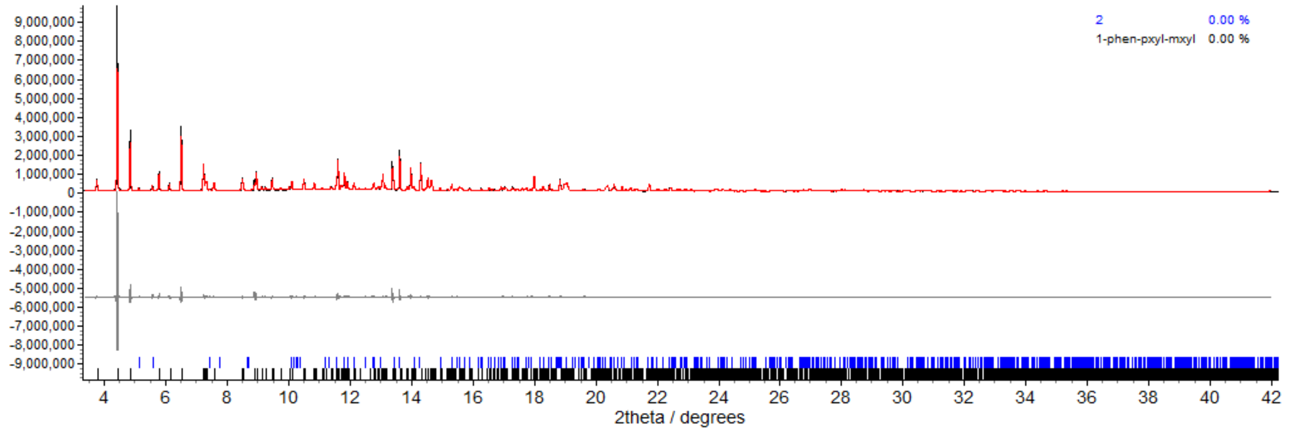

Figure S15. Observed (black) and calculated (red) profiles and difference plot [I_{obs}-I_{calc}] (grey) of the Pawley refinement. (2θ range 3.40 – 42.0 °, d_{min} = 1.15 Å).

Phase-purity check, 1.phen.pxyl$_x$.$C_6H_{6(1-x)}$ (x = 0.62 average by NMR/GC).

(λ = 0.82562 (1) Å)

The pattern was compared with calculated X-ray powder patterns for **1.phen.pxyl** and **2** already established from single-crystal X-ray diffraction. The unit cell parameters of **1.phen.pxyl** and **2** were used as a starting point for Pawley refinement,[S4] employing 3089 parameters (7 background, 1 zero error, 9 profile, 8 cell, 3064 reflections). Pawley refinement converged to R_{wp} = 9.40, $R_{wp'}$ = 17.21. [phase "**1.phen.pxyl$_x$.$C_6H_{6(1-x)}$**": a = 30.8043(6) Å, b = 10.4024(2) Å, c = 26.1674(5) Å, β = 125.657(1)°, V = 6813.1(2) Å^3; phase "**2**": a = 24.5305(6) Å, b = 5.7212(1) Å, c = 16.9661(4) Å, β = 131.455(1)°, V = 1784.57(1) Å^3].

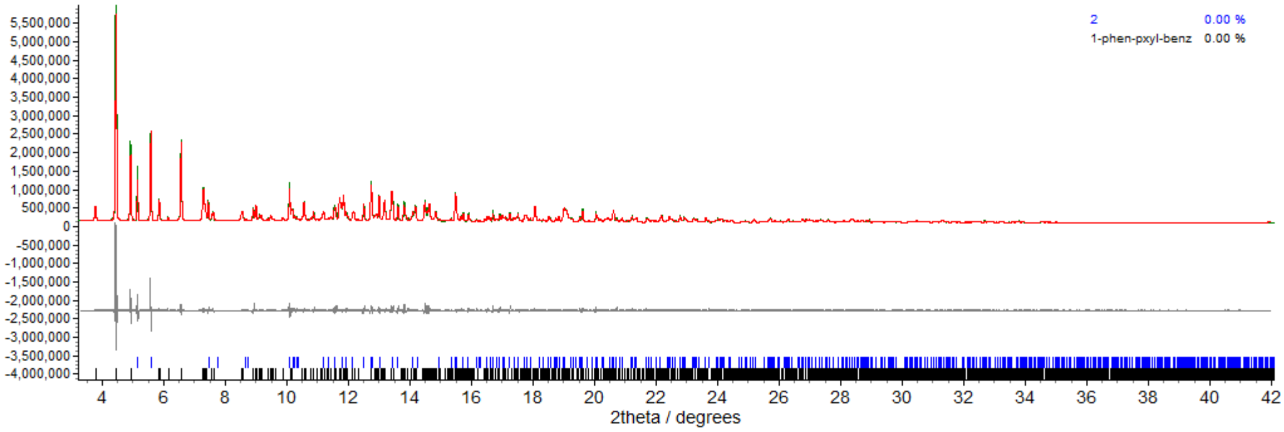

Figure S16. Observed (green) and calculated (red) profiles and difference plot [I_{obs}-I_{calc}] (grey) of the Pawley refinement. (2θ range 3.40 – 42.0 °, d_{min} = 1.15 Å).

3.4. Mixed benzene / other arene systems

In all cases, visual inspection of the X-ray powder diffraction patterns indicated the presence of a small amount of the solvent-free crystalline phase **2** (though this is appreciably larger component in these benzene-containing systems). This was therefore included in the Pawley refinement. The peaks corresponding to the solvent free phase **2** are indicated by blue tickmarks in all diagrams below, demonstrating their low intensity and therefore likely small relative content (see Figure S5).

Inclusion of other arenes is however also indicated in these cases by expansion of the unit cell of the solvent-containing phase **1.phen.$C_6H_{6(x)}$."arene"$_{(1-x)}$**, compared with the room temperature unit cell of **1.phen.C_6H_6**. However, the resolution of the single-crystal or powder X-ray diffraction data is not sufficient to enable the proportion of xylene to be determined.

Phase-purity check, 1.phen.$C_6H_{6(x)}$.oxyl$_{(1-x)}$ (x = 0.81 average by NMR/GC).

(λ = 0.826210 (5) Å)

The pattern was compared with calculated X-ray powder patterns for **1.phen.C_6H_6** and **2** already established from single-crystal X-ray diffraction. The unit cell parameters of **1.phen. C_6H_6** and **2** were

used as a starting point for Pawley refinement,[S4] employing 6037 parameters (6 background, 1 zero error, 9 profile, 8 cell, 6013 reflections). Pawley refinement converged to R_{wp} = 5.90, $R_{wp'}$ = 12.14. [phase "**1.phen.$C_6H_{6(x)}$.oxyl$_{(1-x)}$**": *a* = 30.8326(2) Å, *b* = 10.24680(7) Å, *c* = 26.0010(2) Å, β = 125.3680(5)°, *V* = 6700.9(1) Å^3; phase "**2**": *a* = 24.4952(5) Å, *b* = 5.7359(1) Å, *c* = 16.9541(4) Å, β = 131.429(2)°, *V* = 1786.03(9) Å^3].

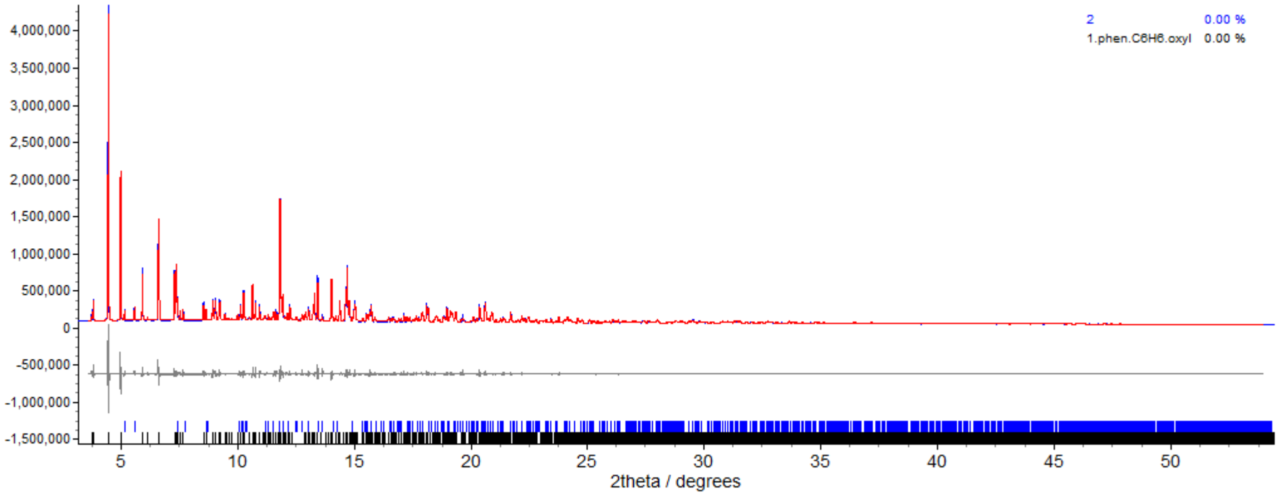

Figure S17. Observed (blue) and calculated (red) profiles and difference plot [I_{obs}-I_{calc}] (grey) of the Pawley refinement. (2θ range 3.60 – 54 °, d_{min} = 0.91 Å).

Phase-purity check, 1.phen.$C_6H_{6(x)}$.mxyl$_{(1-x)}$ (x = 0.93 average by NMR/GC).

(λ = 0.826210 (5) Å)

The pattern was compared with calculated X-ray powder patterns for **1.phen.C_6H_6** and **2** already established from single-crystal X-ray diffraction. The unit cell parameters of **1.phen. C_6H_6** and **2** were used as a starting point for Pawley refinement,[S4] employing 6059 parameters (6 background, 1 zero error, 9 profile, 8 cell, 6035 reflections). Pawley refinement converged to R_{wp} = 7.31, $R_{wp'}$ = 15.99. [phase "**1.phen.$C_6H_{6(x)}$.mxyl$_{(1-x)}$**": *a* = 30.8212(3) Å, *b* = 10.25504(8) Å, *c* = 25.9970(2) Å, β = 125.4673(7)°, *V* = 6692.1(1) Å^3; phase "**2**": *a* = 24.4947(4) Å, *b* = 5.7162(1) Å, *c* = 16.9482(2) Å, β = 131.487(1)°, *V* = 1777.65(5) Å^3].

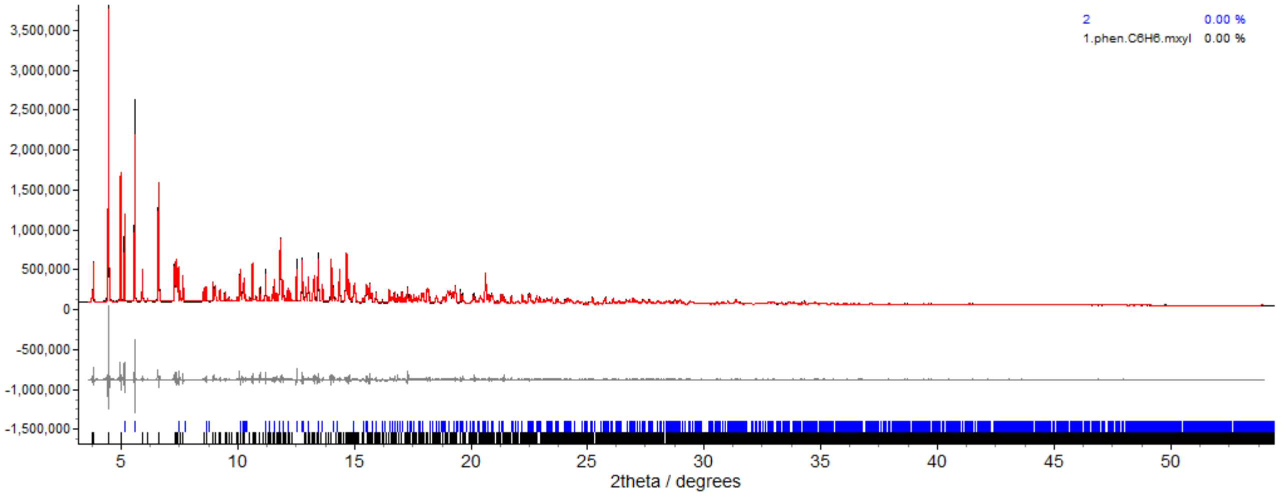

Figure S18. Observed (black) and calculated (red) profiles and difference plot [I_{obs}-I_{calc}] (grey) of the Pawley refinement. (2θ range 3.60 – 54 °, d_{min} = 0.91 Å).

4. ^{1}H-NMR spectroscopic analysis of mixed arene investigations

The ^{1}H NMR spectra, acquired as indicated in the Experimental Section, were analysed using Bruker Topspin 3.2. The integrated peak areas for the aromatic and aliphatic proton signals of the arene guests (calibrating the integrals upon the assumption that the aromatic proton signal for phenazine represents exactly 32 protons per formula unit) were compared, scaling for the different number of protons represented by each, to give an overall arene ratio in the cases of mixed arene systems. The proton signals in all following spectra are labelled as shown in Figure **S19**.

The ratio of arene guests in mixed systems can thus be determined from appropriately weighted integrals for the two guests.

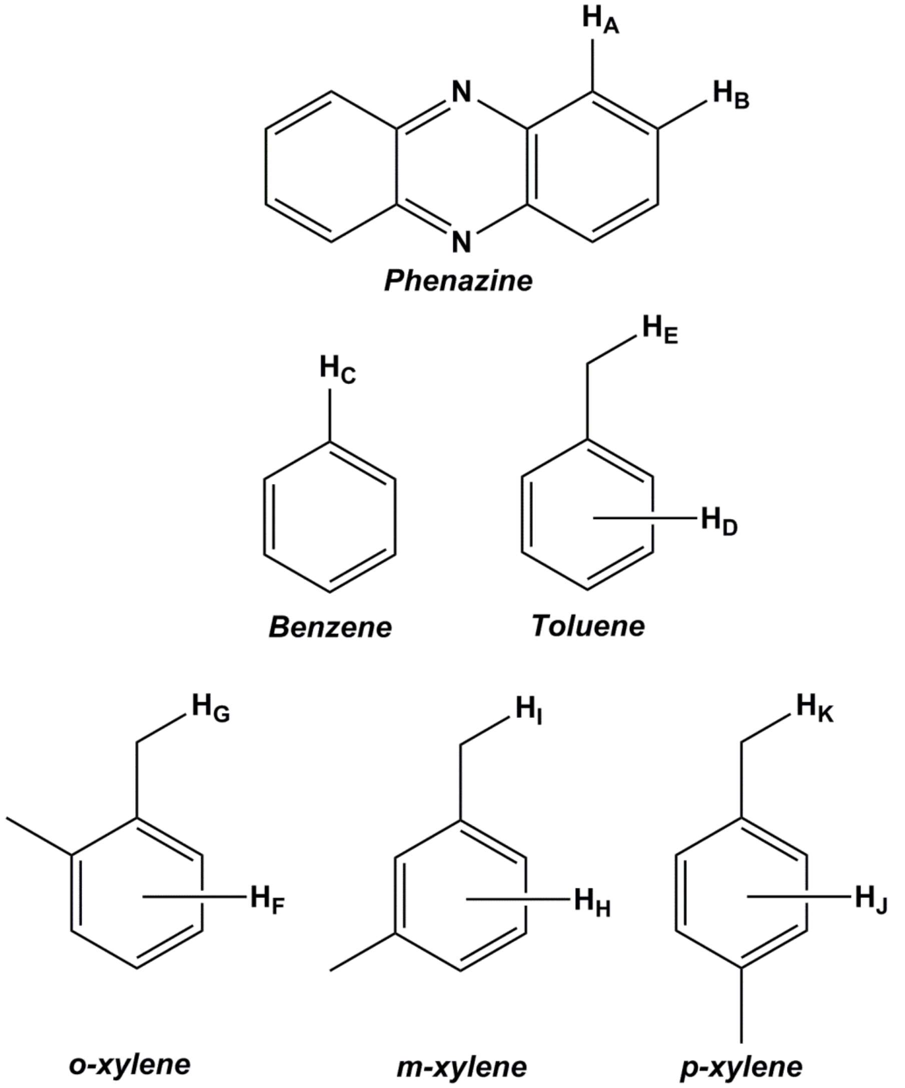

Figure S19. ^{1}H-NMR environment labels used for all spectra shown in this section.

4.1. Pure arene or guest-free systems

^{1}H-NMR, 1.phen.tol.

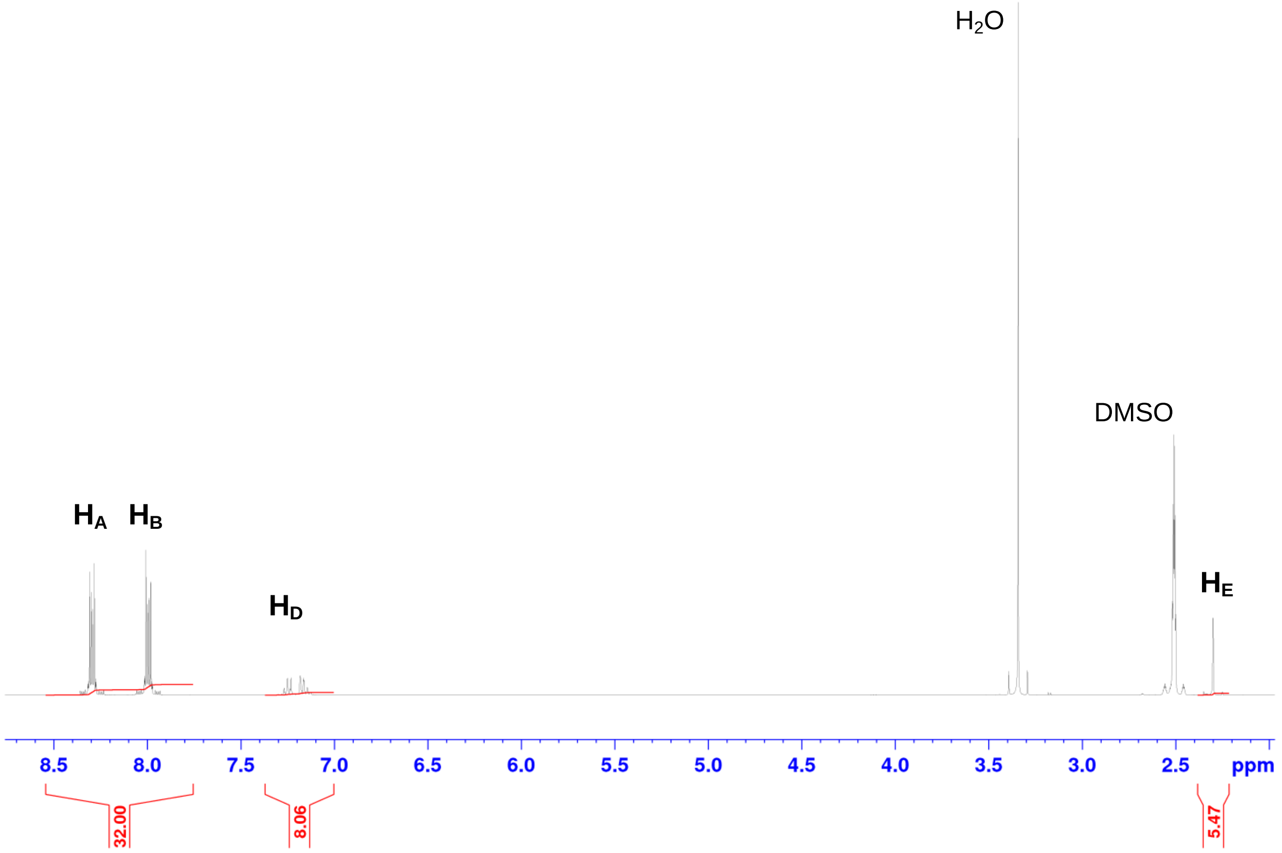

Figure S20. Annotated ^{1}H-NMR spectrum in d6-DMSO for **1.phen.tol**.

^{1}H-NMR, 1.phen.pxyl.

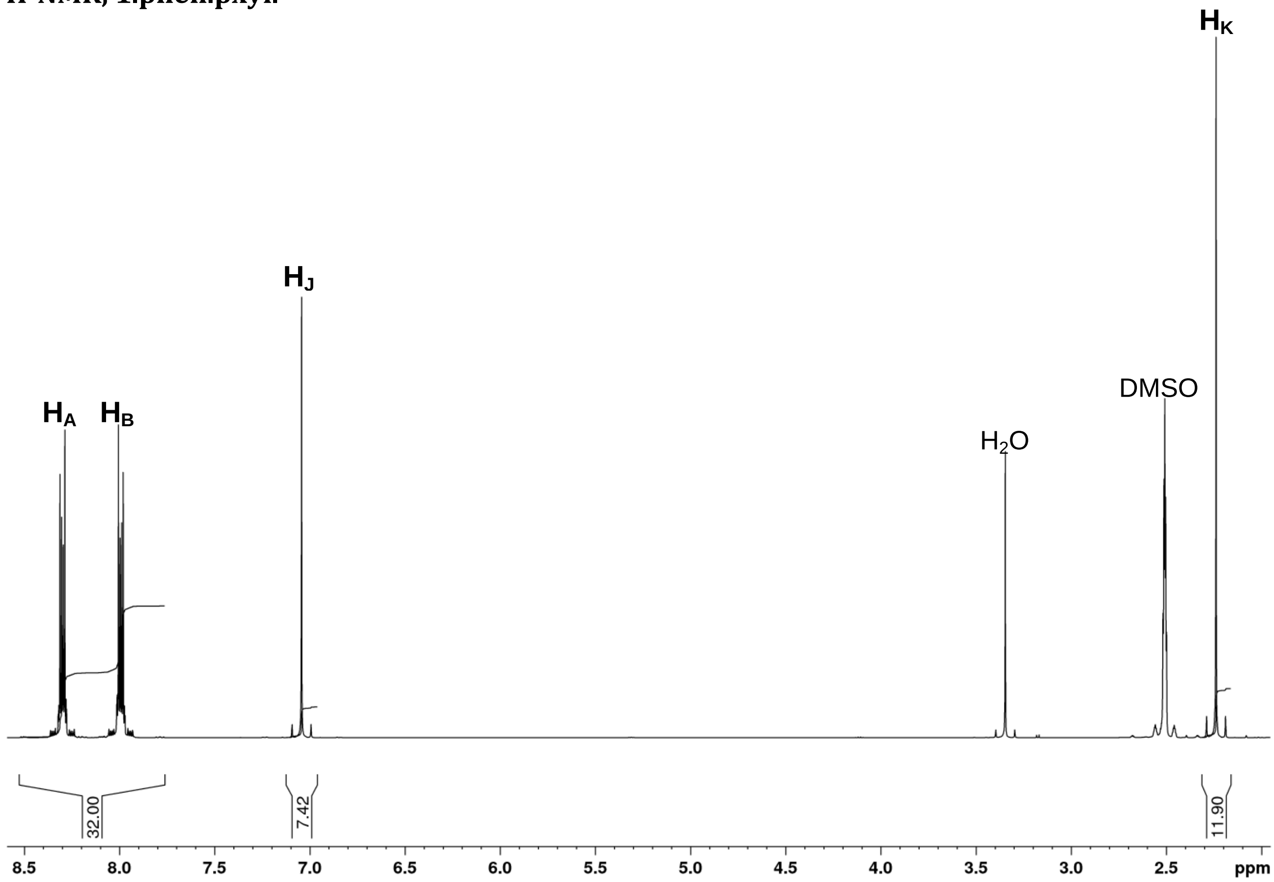

Figure S21. Annotated ^{1}H-NMR spectrum in d6-DMSO for **1.phen.pxyl**.

^{1}H-NMR, 2

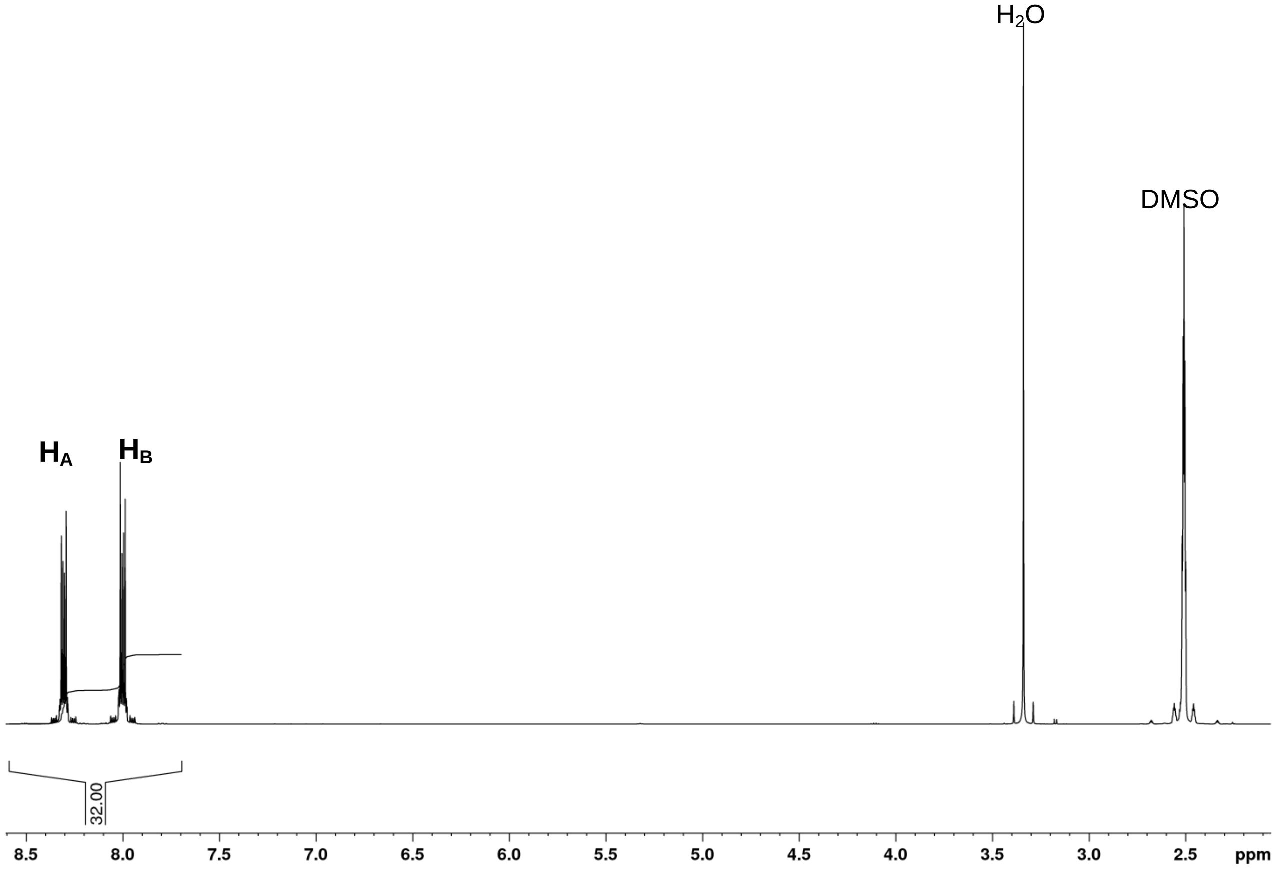

Figure S22. Annotated ^{1}H-NMR spectrum in d6-DMSO for **2**.

^{1}H-NMR, 1.phen.C_6H_6.

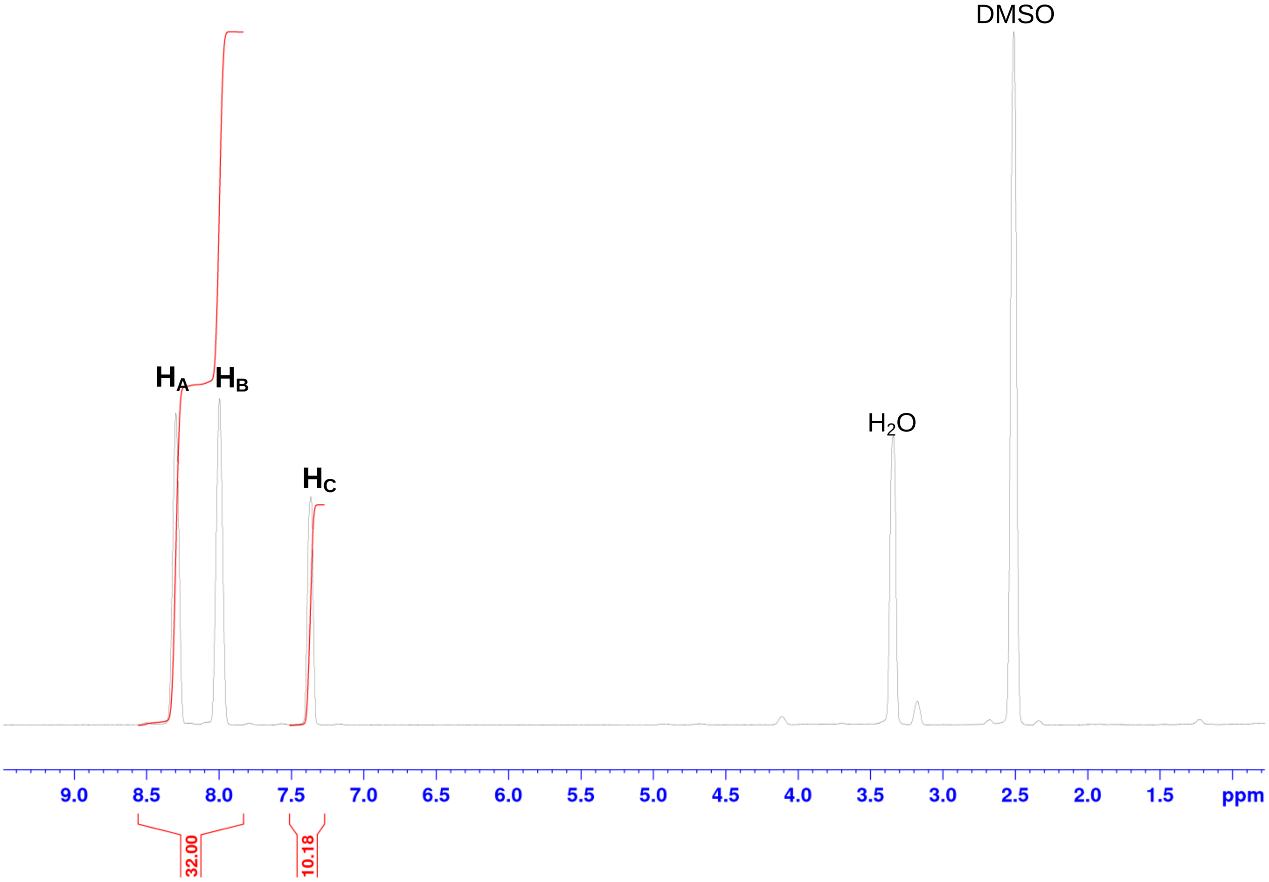

Figure S23. Annotated ^{1}H-NMR spectrum in d6-DMSO for **1.phen.C_6H_6**.

4.2. Mixed toluene/p-xylene systems

^{1}H-NMR, 1.phen.tol$_{x}$.pxyl$_{(1-x)}$ (x = 0.73 average by NMR/GC).

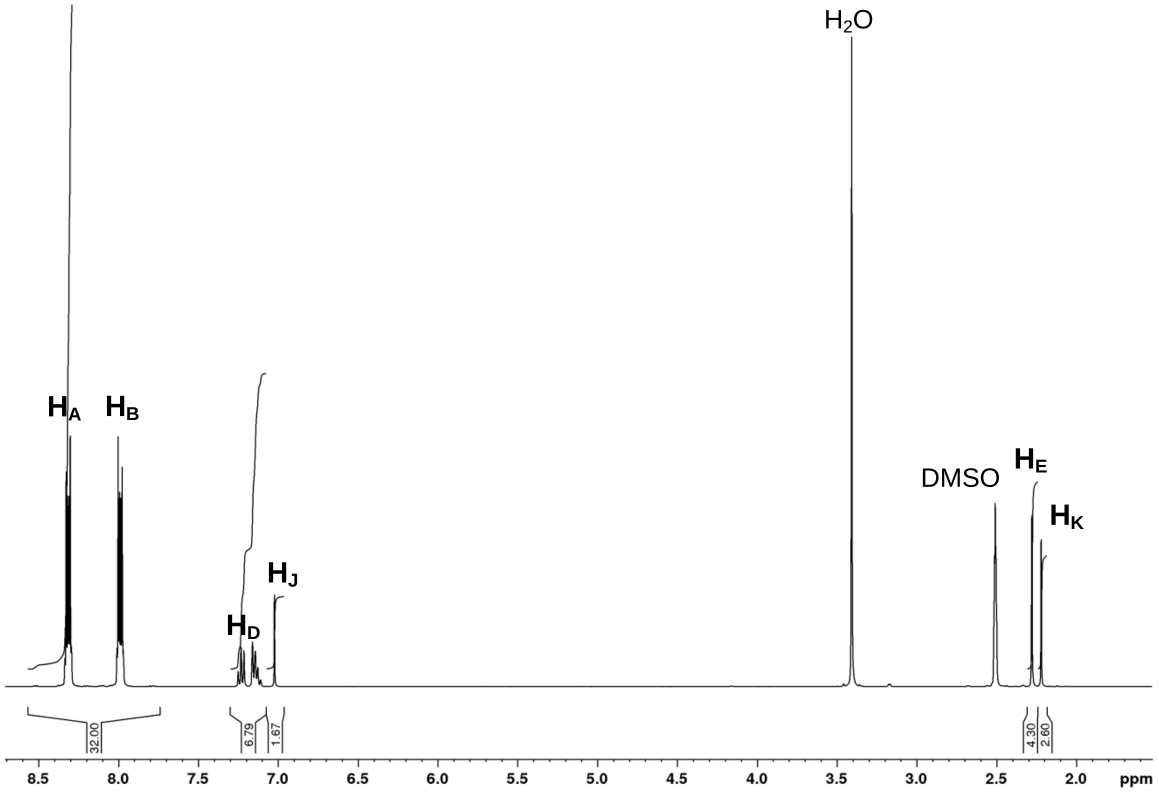

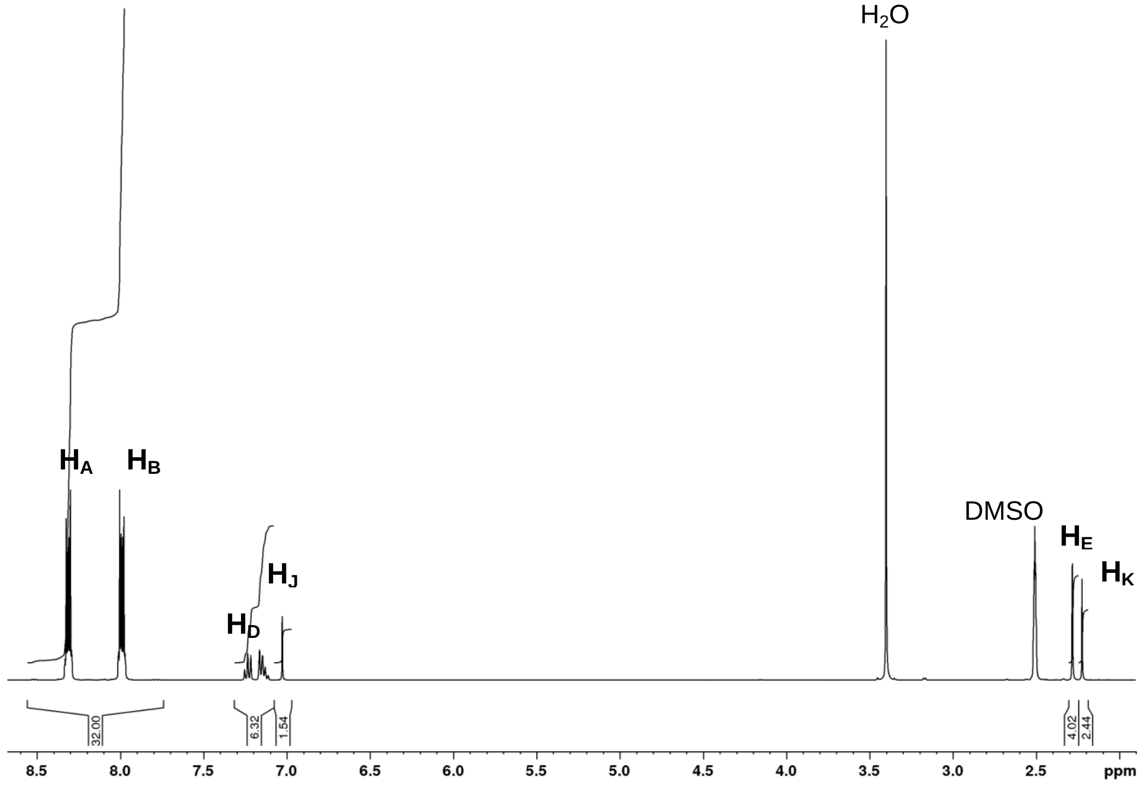

Figure S24. Annotated ^{1}H-NMR spectra in d6-DMSO for **1.phen.tol$_{x}$.pxyl$_{(1-x)}$**.

^{1}H-NMR, 1.phen.tol$_x$.pxyl$_{(1-x)}$ (x = 0.49 average by NMR/GC).

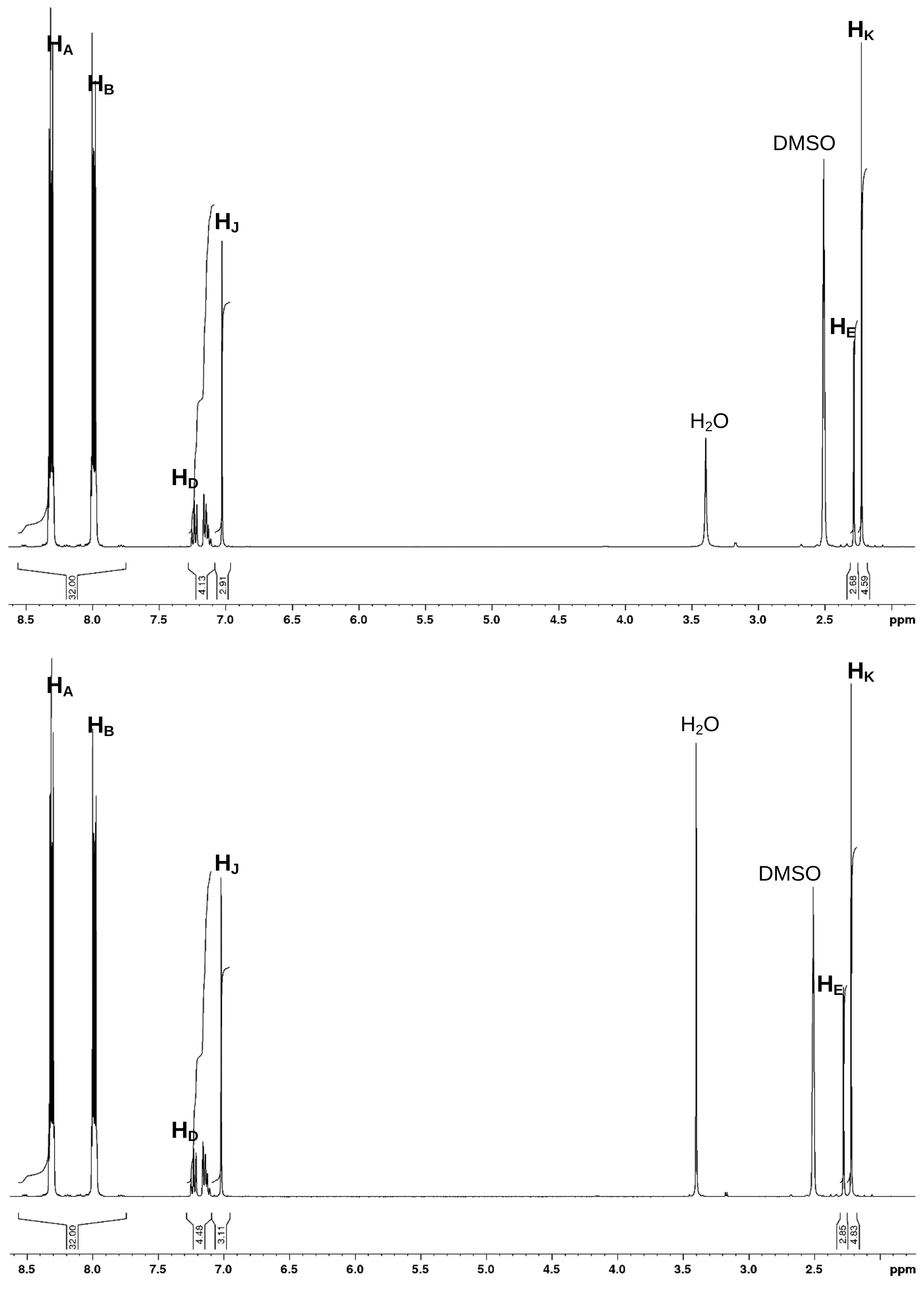

Figure S25. Annotated ^{1}H-NMR spectra in d6-DMSO for **1.phen.tol$_x$.pxyl$_{(1-x)}$**.

^{1}H-NMR, 1.phen.tol$_{x}$.pxyl$_{(1-x)}$ (x = 0.38 average by NMR/GC).

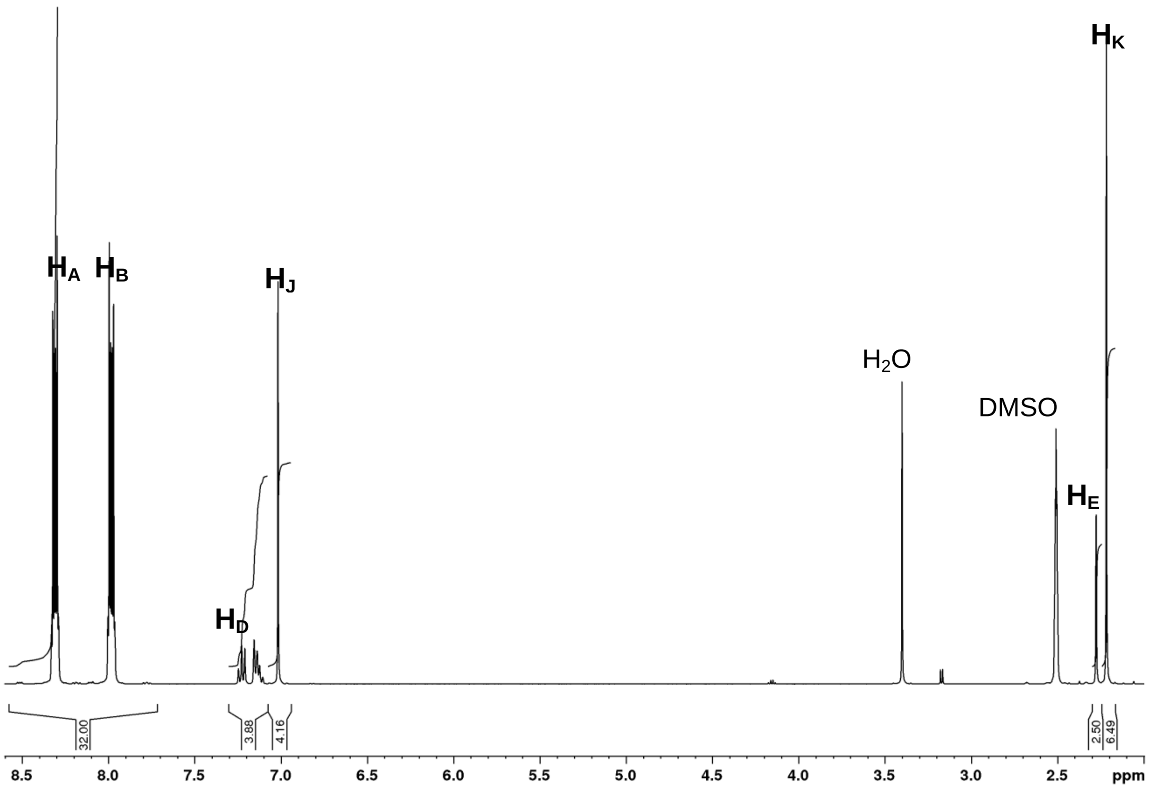

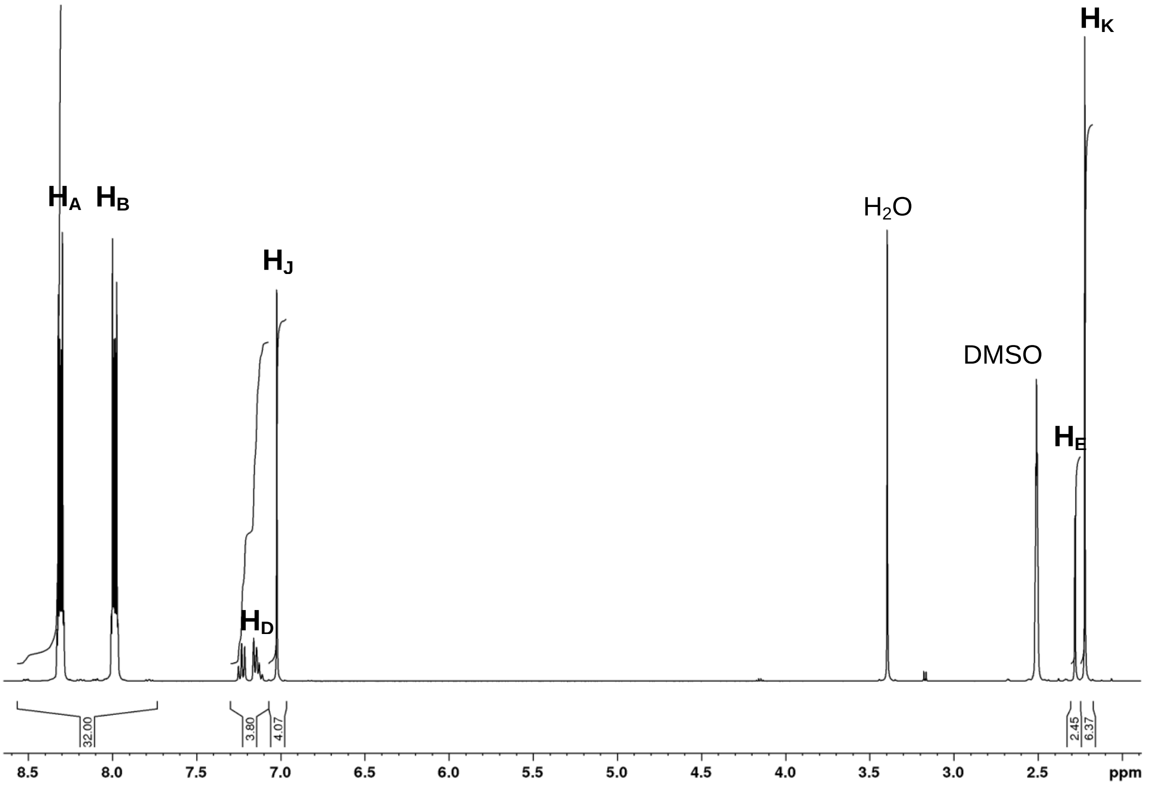

Figure S26. Annotated ^{1}H-NMR spectra in d6-DMSO for **1.phen.tol$_{x}$.pxyl$_{(1-x)}$**.

^{1}H-NMR, 1.phen.tol$_{x}$.pxyl$_{(1-x)}$ (x = 0.30 average by NMR/GC).

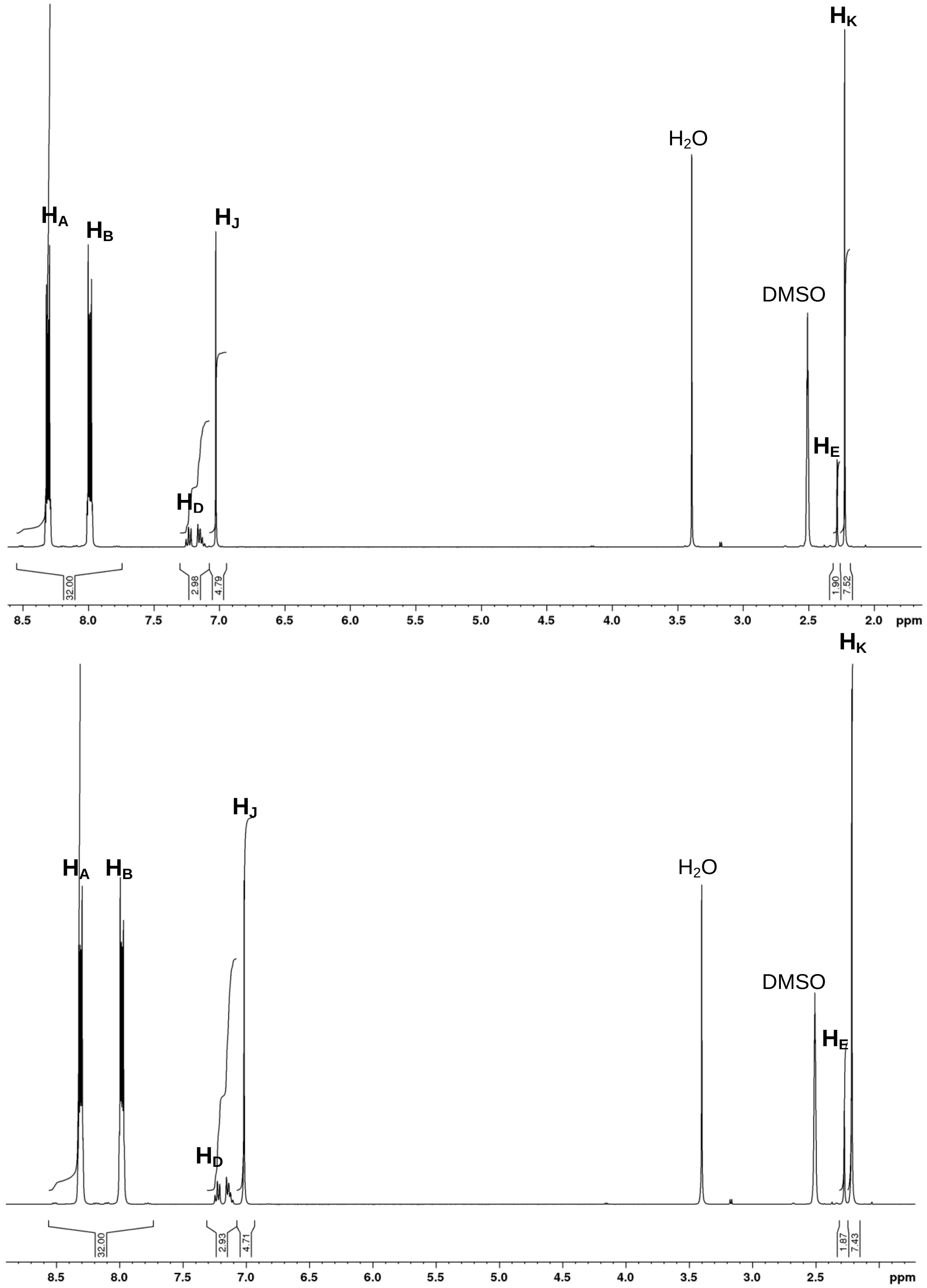

Figure S27. Annotated ^{1}H-NMR spectra in d6-DMSO for **1.phen.tol$_{x}$.pxyl$_{(1-x)}$**.

^{1}H-NMR, 1.phen.tol$_{x}$.pxyl$_{(1-x)}$ (x = 0.14 average by NMR/GC).

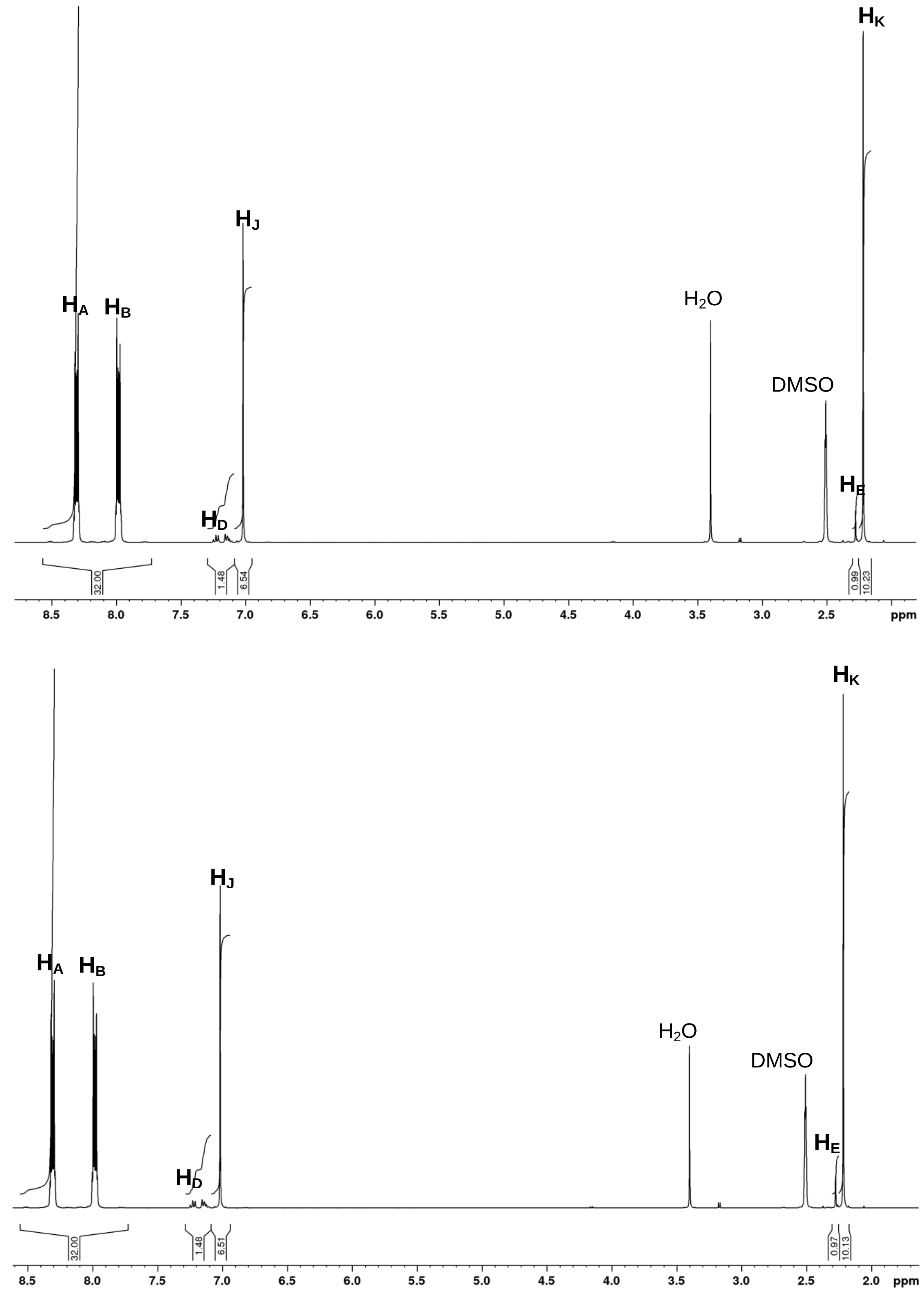

Figure S28. Annotated ^{1}H-NMR spectra in d6-DMSO for **1.phen.tol$_{x}$.pxyl$_{(1-x)}$**.

4.3. Mixed toluene/other arene systems

^{1}H-NMR, 1.phen.tol$_x$.oxyl$_{(1-x)}$ (x = 0.79 average by NMR/GC).

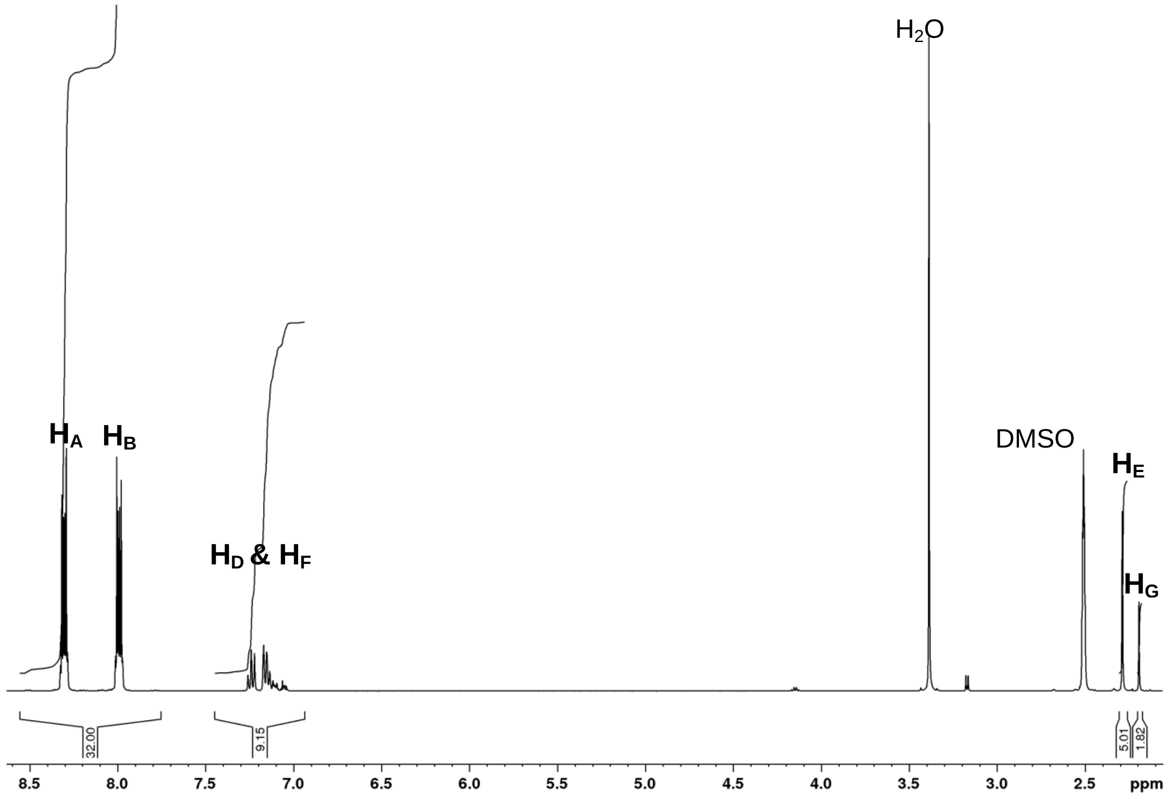

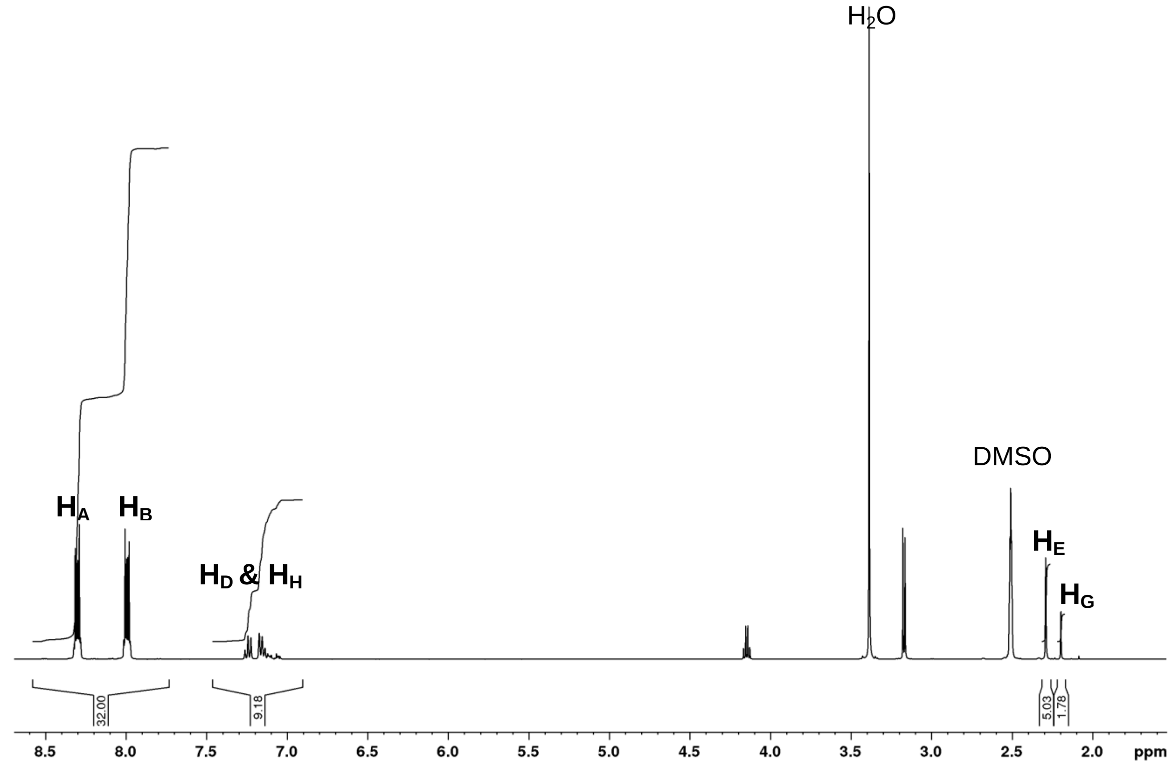

Figure S29. Annotated ^{1}H-NMR spectra in d6-DMSO for **1.phen.tol$_x$.oxyl$_{(1-x)}$**.

^{1}H-NMR, 1.phen.tol$_x$.mxyl$_{(1-x)}$ (x = 0.87 average by NMR/GC).

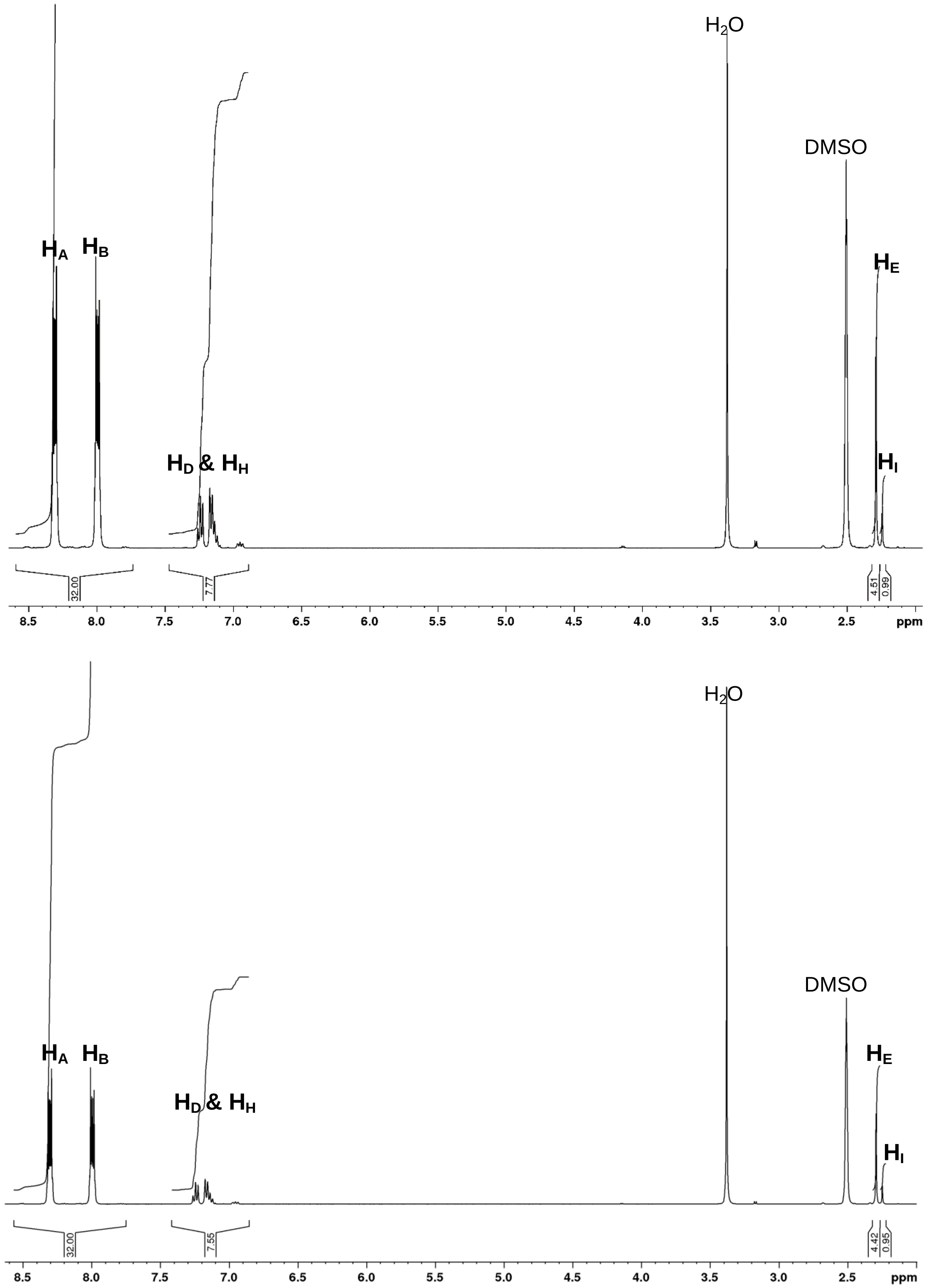

Figure S30. Annotated ^{1}H-NMR spectra in d6-DMSO for **1.phen.tol$_x$.mxyl$_{(1-x)}$**.

^{1}H-NMR, 1.phen.tol$_x$.C$_6$H$_{6(1-x)}$ (x = 0.46 average by NMR/GC).

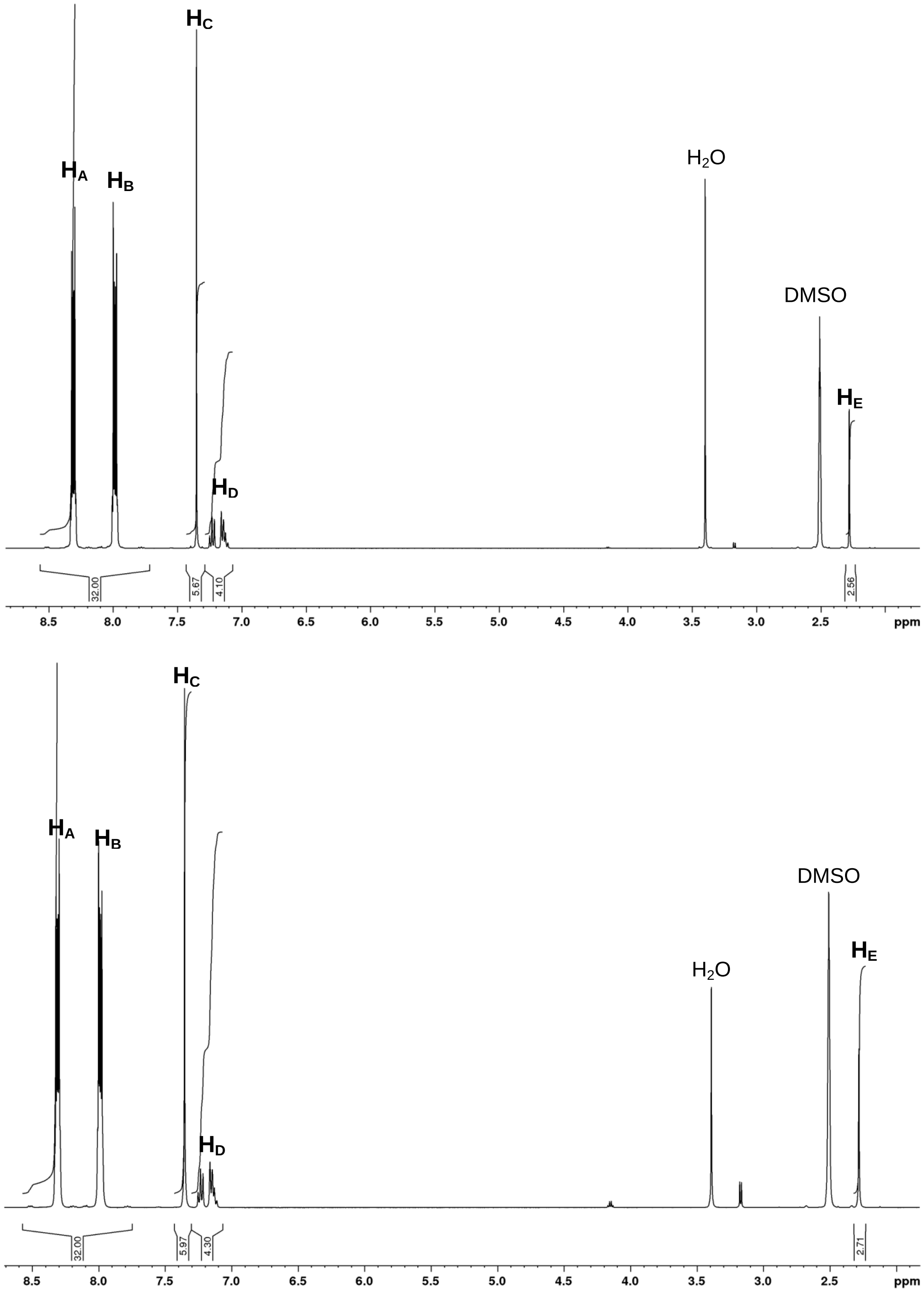

Figure S31. Annotated ^{1}H-NMR spectra in d6-DMSO for **1.phen.tol$_x$.benz$_{(1-x)}$**.

4.4. Mixed p-xylene/other arene systems

^{1}H-NMR, 1.phen.pxyl$_{x}$.oxyl$_{(1-x)}$ (x = 0.90 average by NMR/GC).

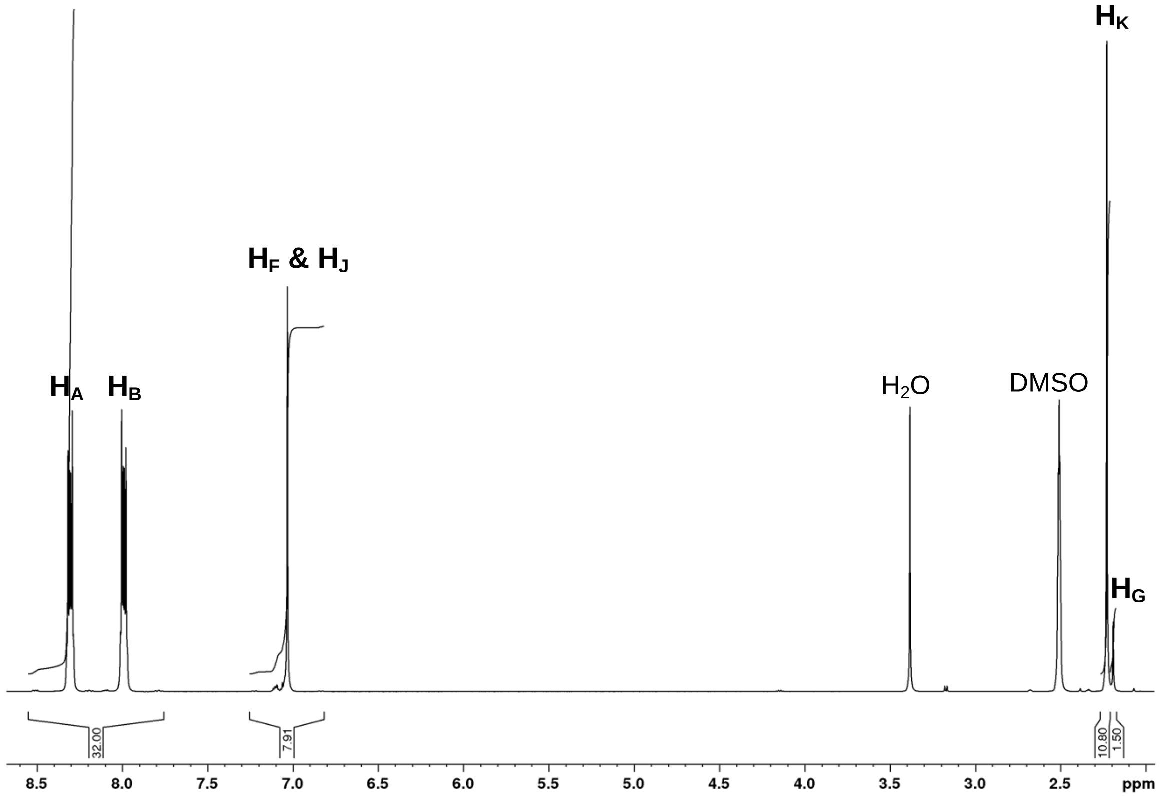

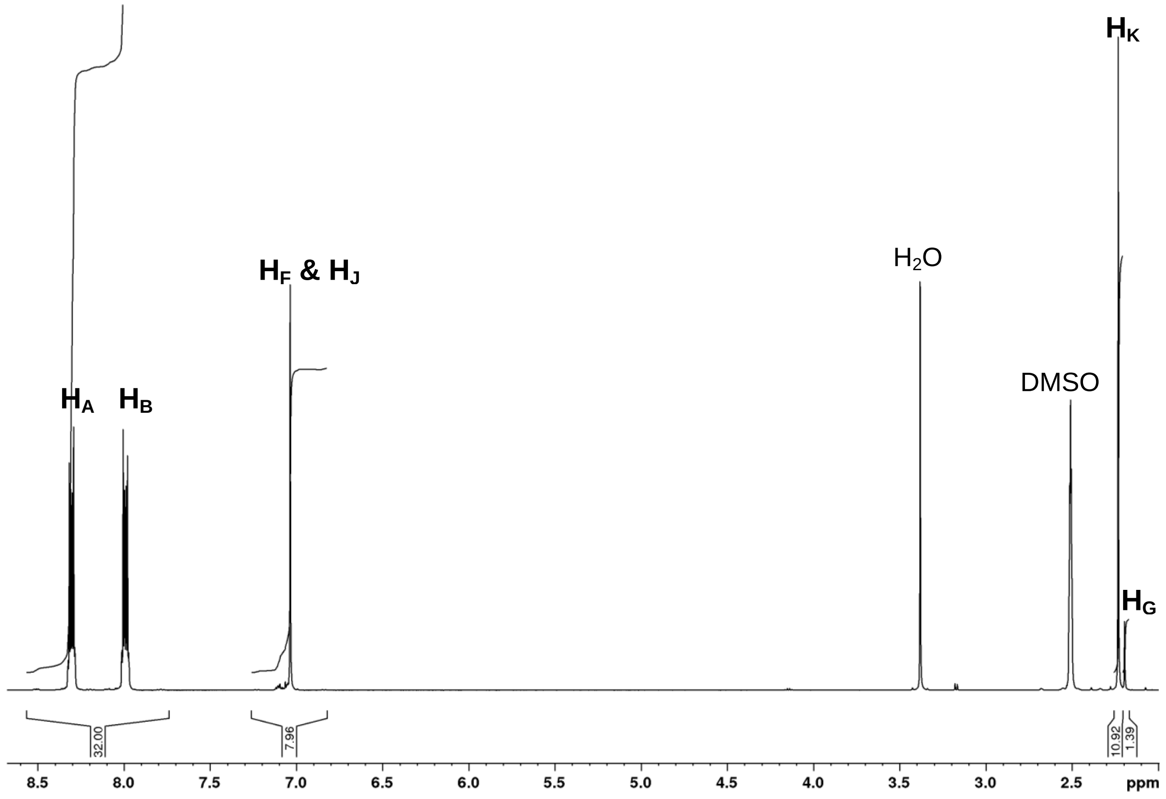

Figure S32. Annotated ^{1}H-NMR spectra in d6-DMSO for **1.phen.pxyl$_{x}$.oxyl$_{(1-x)}$**.

^{1}H-NMR, 1.phen.pxyl$_x$.mxyl$_{(1-x)}$ (x = 0.93 by NMR).

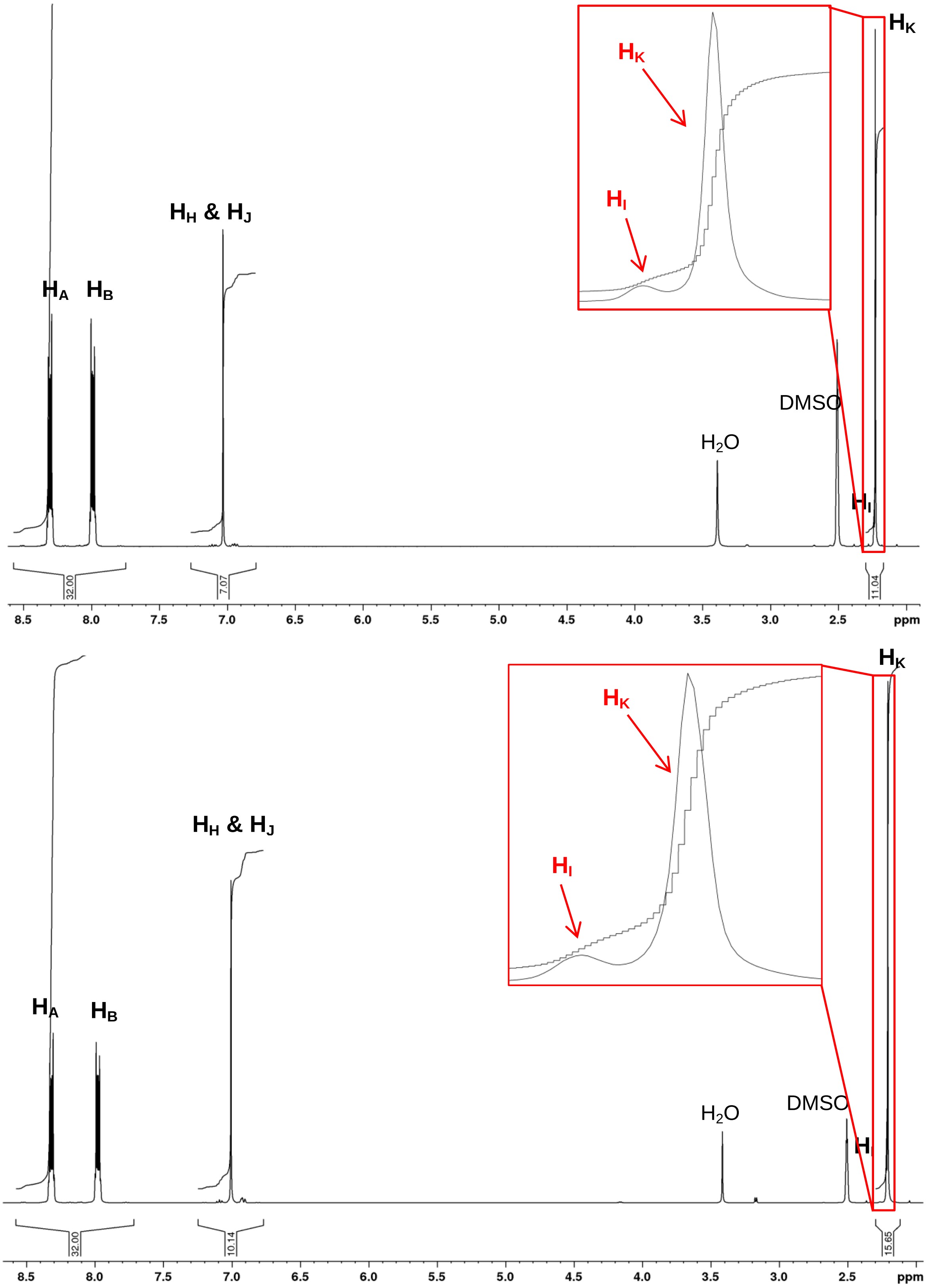

Figure S33 (continued on next page). Annotated ^{1}H-NMR spectra (and expansions showing the presence of *m*-xylene) in d6-DMSO for **1.phen.pxyl$_x$.mxyl$_{(1-x)}$**, from four separate batches. Deconvolution of the **H$_I$** and **H$_K$** singlets enabled the –CH_3 integrals to be determined.

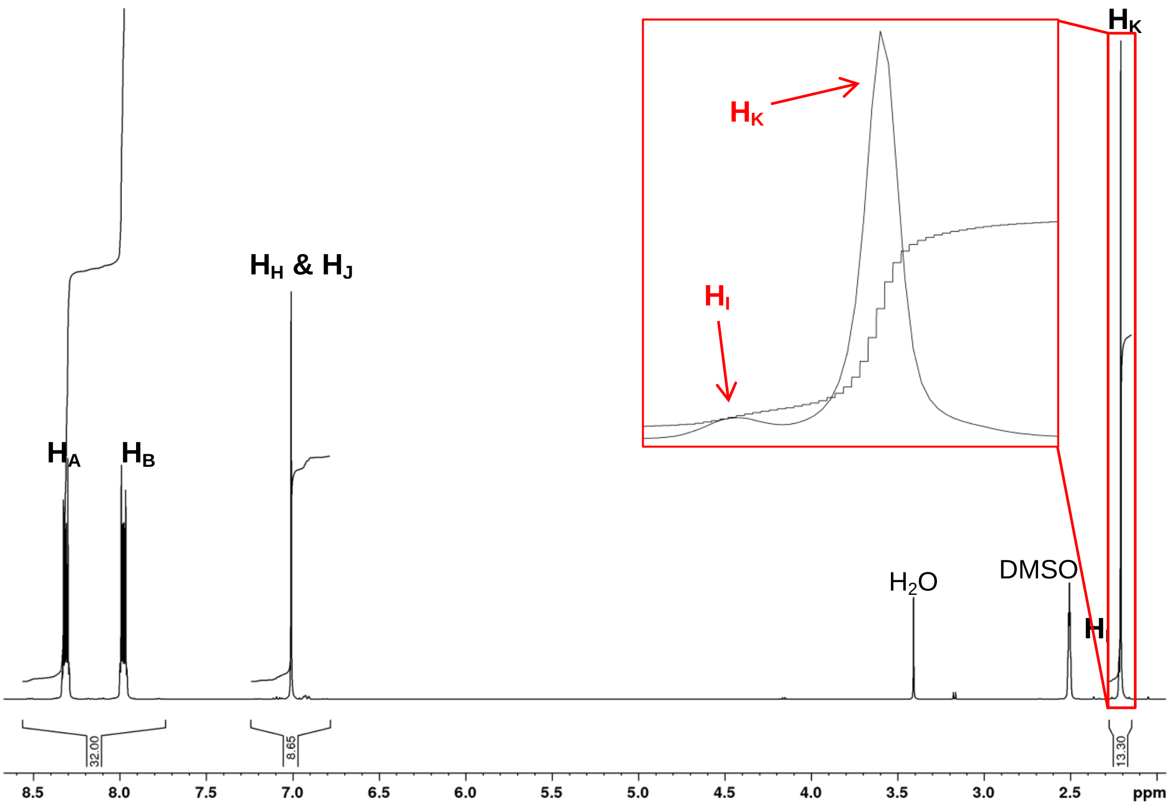

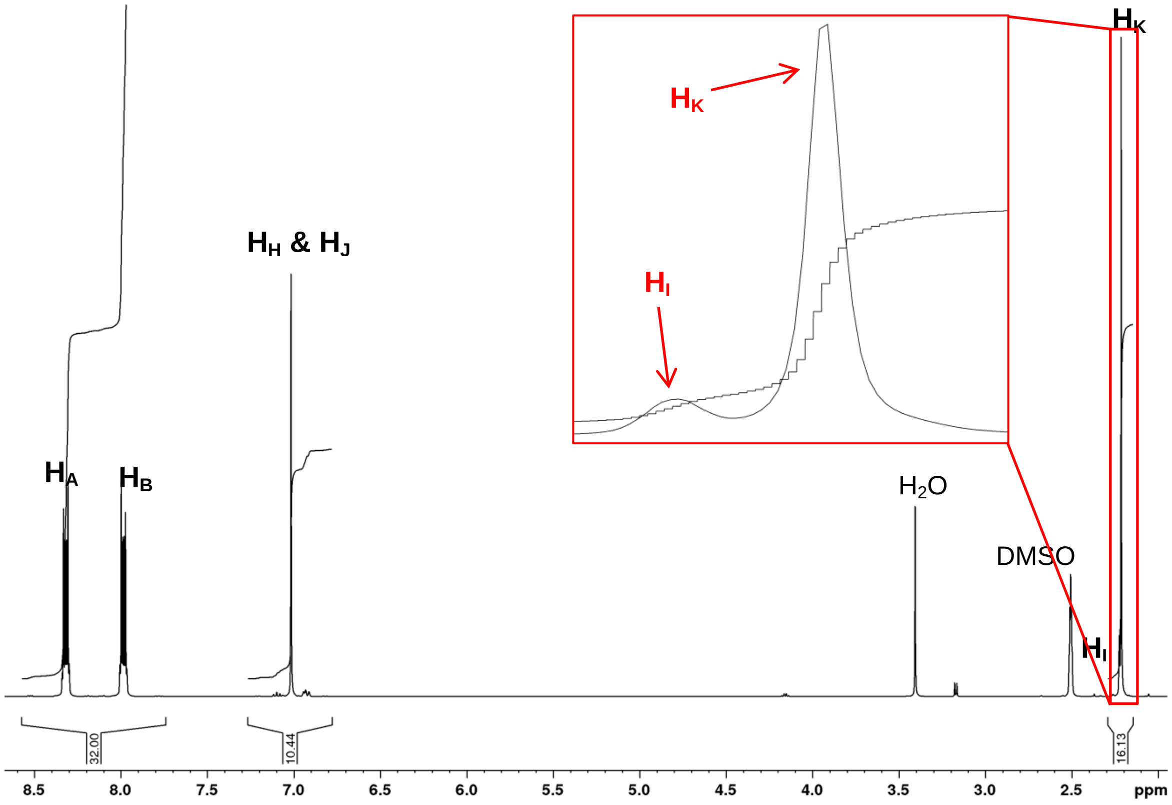

Figure S33 (continued). Annotated ^{1}H-NMR spectra (and expansions showing the presence of *m*-xylene) in d6-DMSO for **1.phen.pxyl$_x$.mxyl$_{(1-x)}$**, from four separate batches. Deconvolution of the **H$_I$** and **H$_K$** singlets allowed the $–CH_3$ integrals to be determined.

^{1}H-NMR, 1.phen.pxyl$_x$. $C_6H_{6\ (1-x)}$ (x = 0.58 average by NMR/GC).

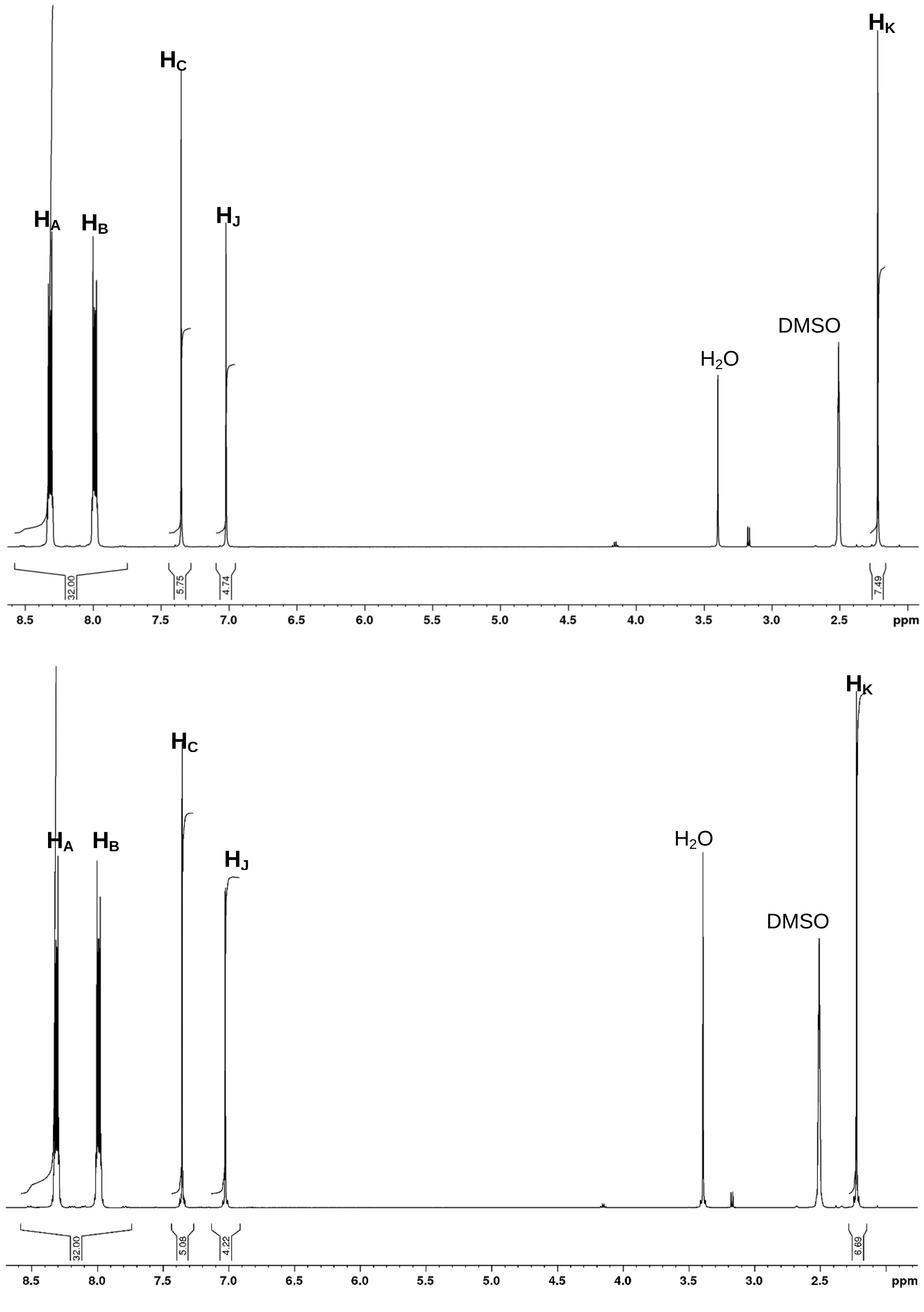

Figure S34. Annotated ^{1}H-NMR spectra in d6-DMSO for **1.phen.pxyl$_x$.$C_6H_{6\ (1-x)}$**.

4.5. Mixed benzene/other arene systems

^{1}H-NMR, 1.phen. $C_6H_{6(x)}$.oxyl$_{(1-x)}$ (x = 0.83 average by NMR/GC).

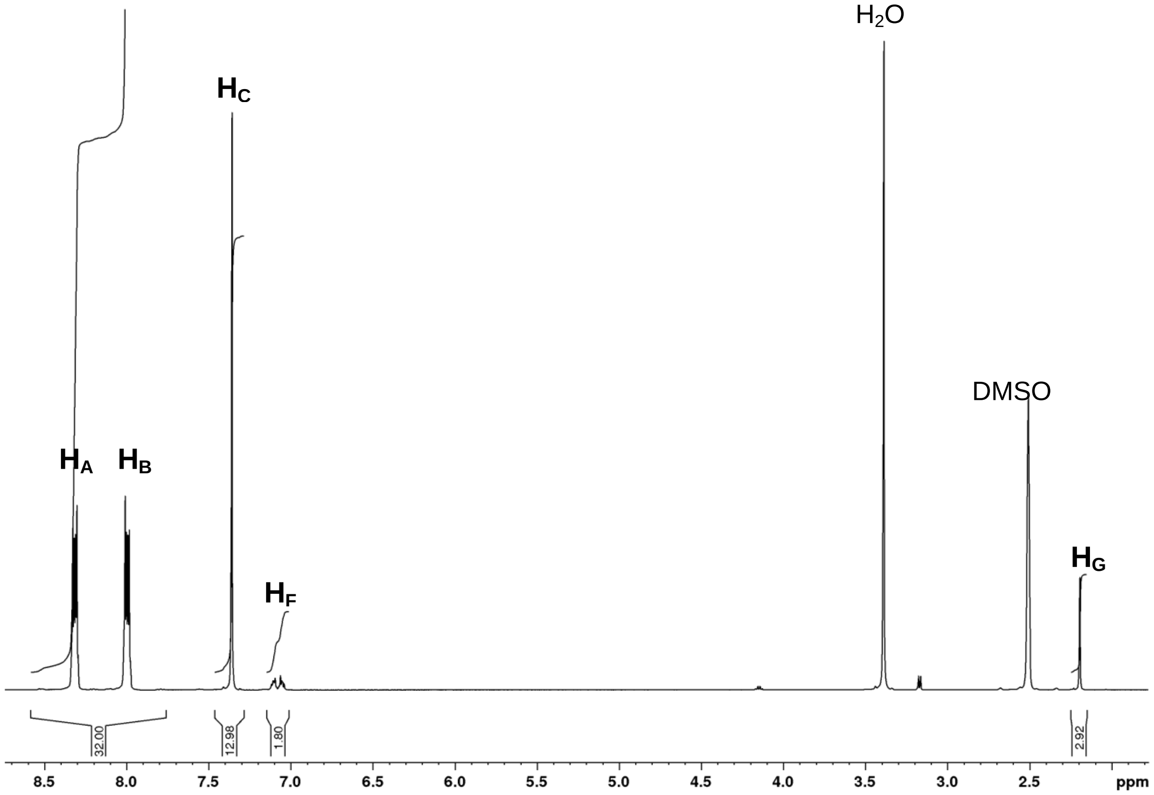

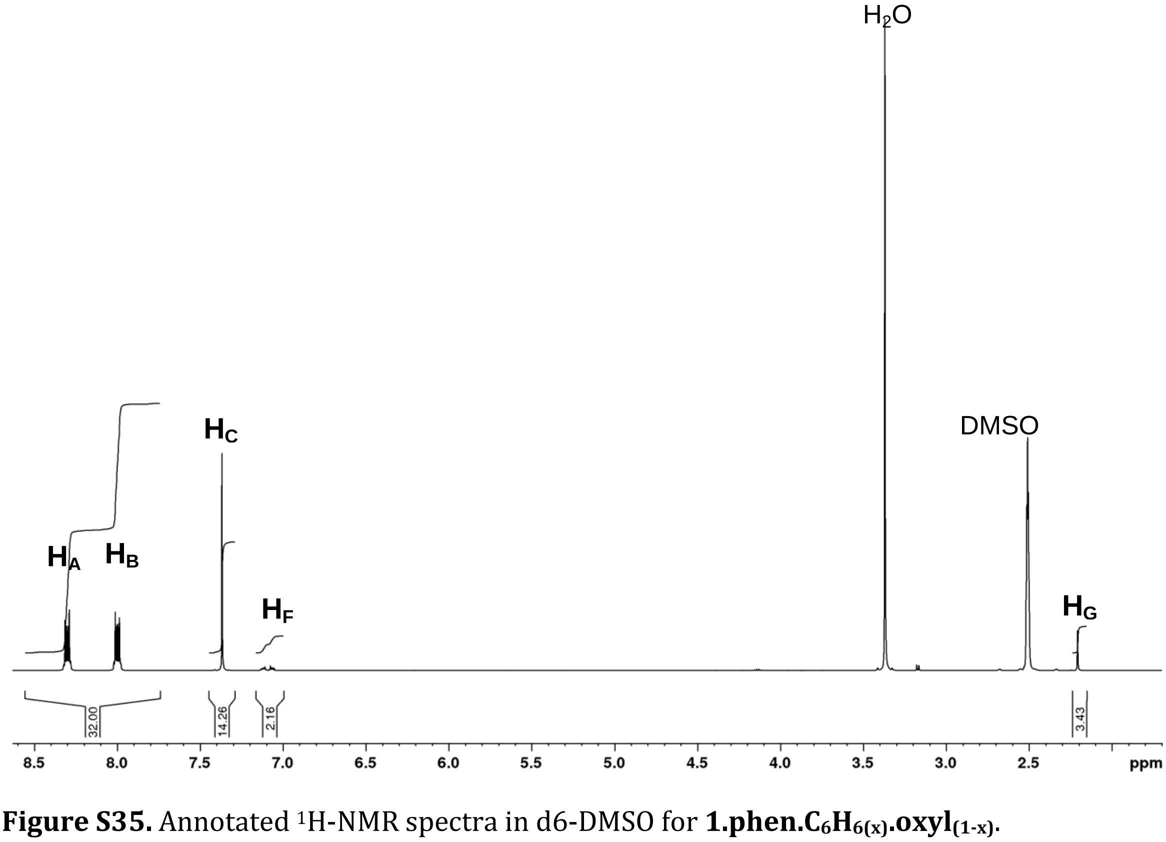

Figure S35. Annotated ^{1}H-NMR spectra in d6-DMSO for **1.phen.$C_6H_{6(x)}$.oxyl$_{(1-x)}$**.

^{1}H-NMR, 1.phen.$C_6H_{6(x)}$.mxyl$_{(1-x)}$ (x = 0.93 average by NMR/GC).

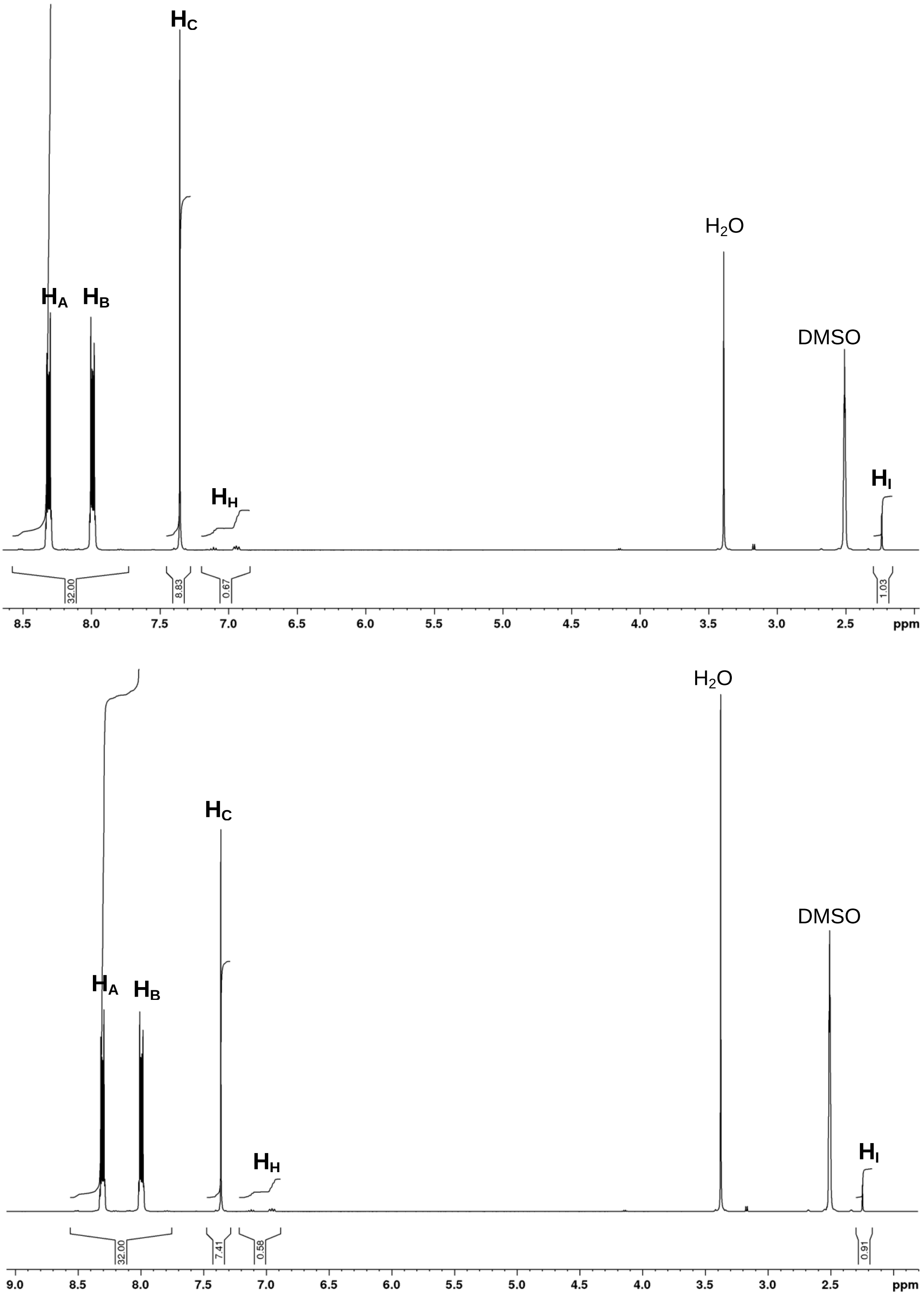

Figure S36. Annotated ^{1}H-NMR spectra in d6-DMSO for **1.phen.$C_6H_{6(x)}$.mxyl$_{(1-x)}$**.

5. Gas chromatographic analysis of mixed arene investigations

The gas chromatograms, acquired as indicated in the Experimental Section were analysed using Totalchrom v3. The integrated peak areas for the arene guests were directly compared to quantify the relative amounts of each present. Guest retention times were found to be 9.9 min (benzene), 12.7 min (toluene), 15.1 min (*p*-xylene), 15.2 min (*m*-xylene – indistinguishable from *p*-xylene) and 15.7 min (*o*-xylene). DMSO is observable in all chromatograms as a broad peak with retention time from 13 min to 14 min. In all cases a small peak at approximately 14.88 min was observed, with unknown source. This may correspond to impurities in the column.

5.1. Mixed toluene / *p*-xylene systems

Gas chromatograms, 1.phen.tol$_x$.pxyl$_{(1-x)}$ (x = 0.73 average by NMR/GC).

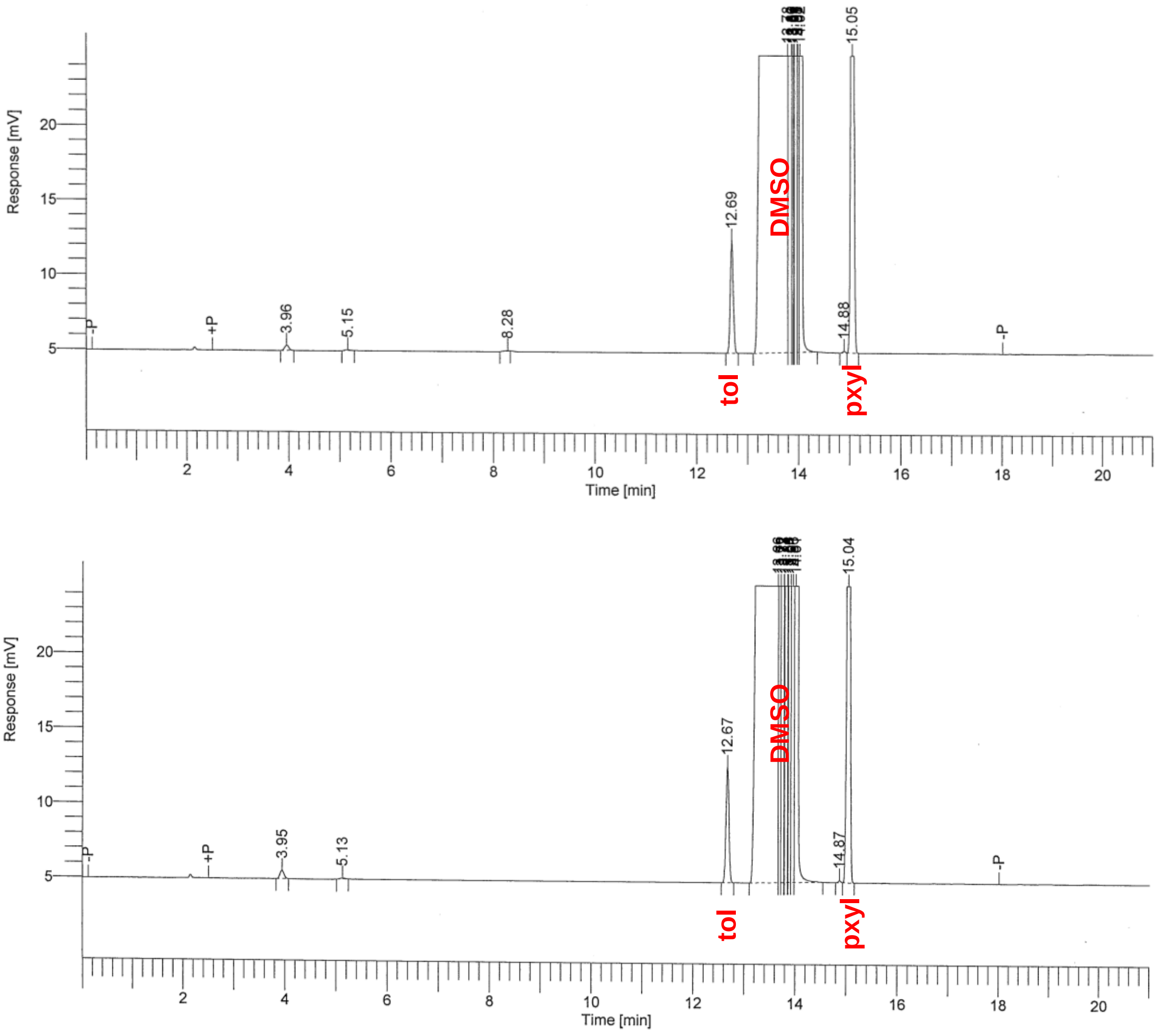

Figure S37. Annotated gas chromatograms in DMSO for **1.phen.tol$_x$.pxyl$_{(1-x)}$**.

Gas chromatograms, 1.phen.tol$_x$.pxyl$_{(1-x)}$ (x = 0.49 average by NMR/GC).

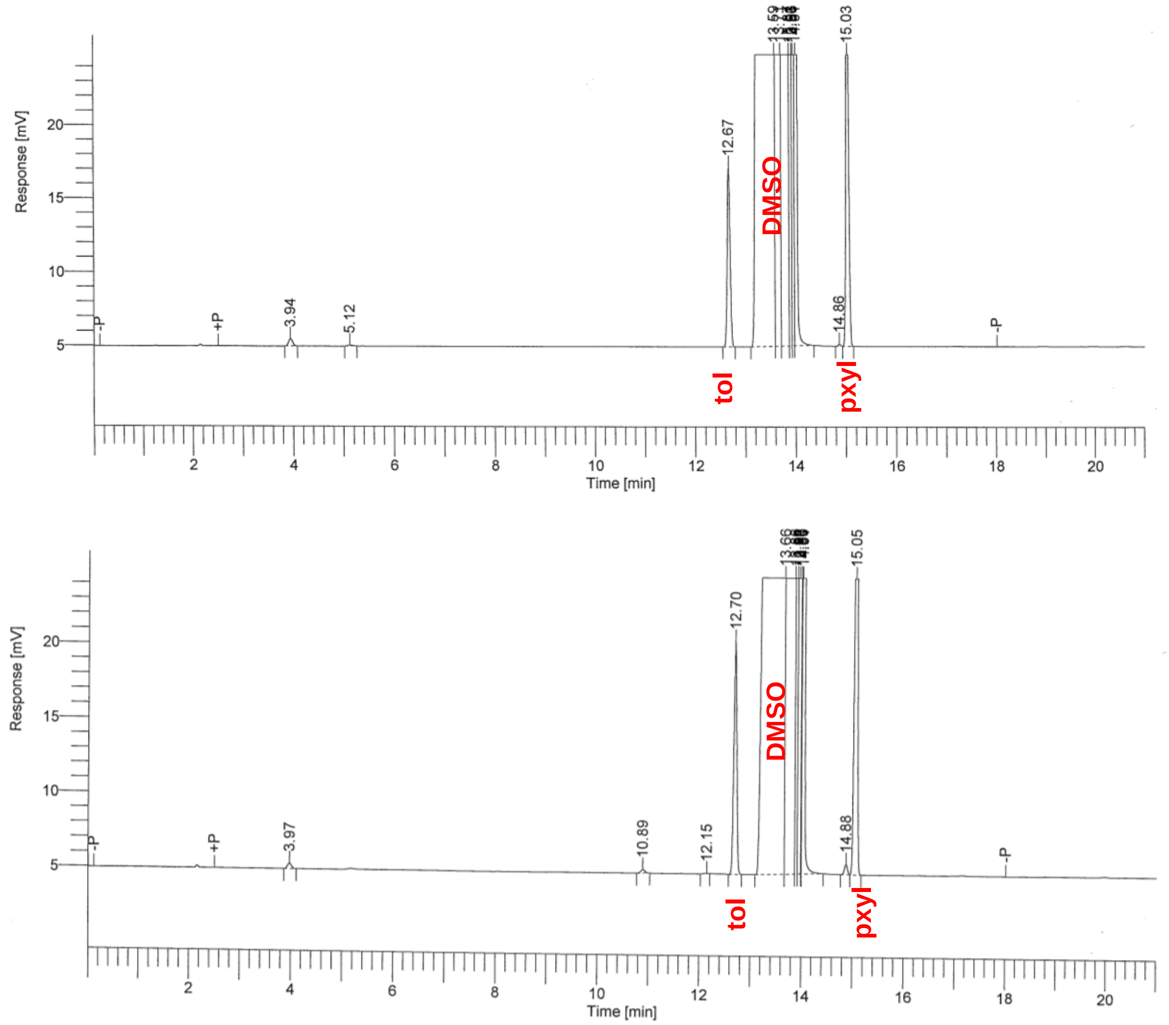

Figure S38. Annotated gas chromatograms in DMSO for **1.phen.tol$_x$.pxyl$_{(1-x)}$**.

Gas chromatograms, 1.phen.tol$_x$.pxyl$_{(1-x)}$ (x = 0.38 average by NMR/GC).

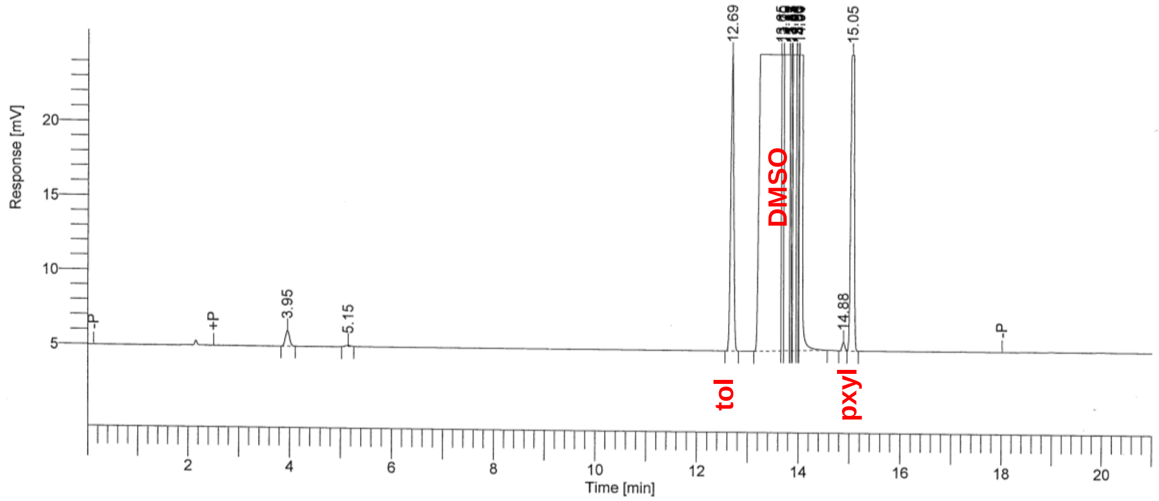

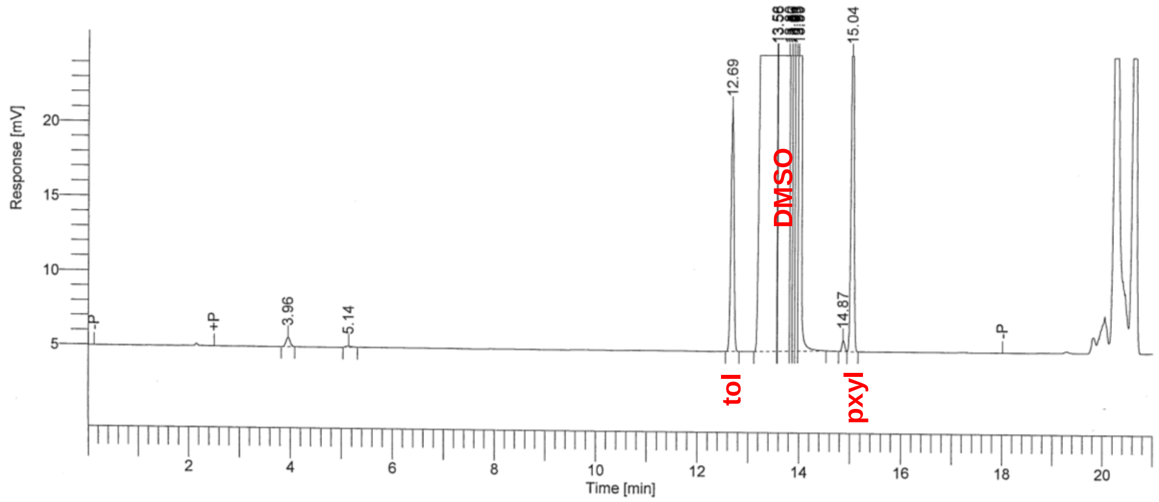

Figure S39. Annotated gas chromatograms in DMSO for **1.phen.tol$_x$.pxyl$_{(1-x)}$**.

Gas chromatograms, 1.phen.tol$_x$.pxyl$_{(1-x)}$ (x = 0.30 average by NMR/GC).

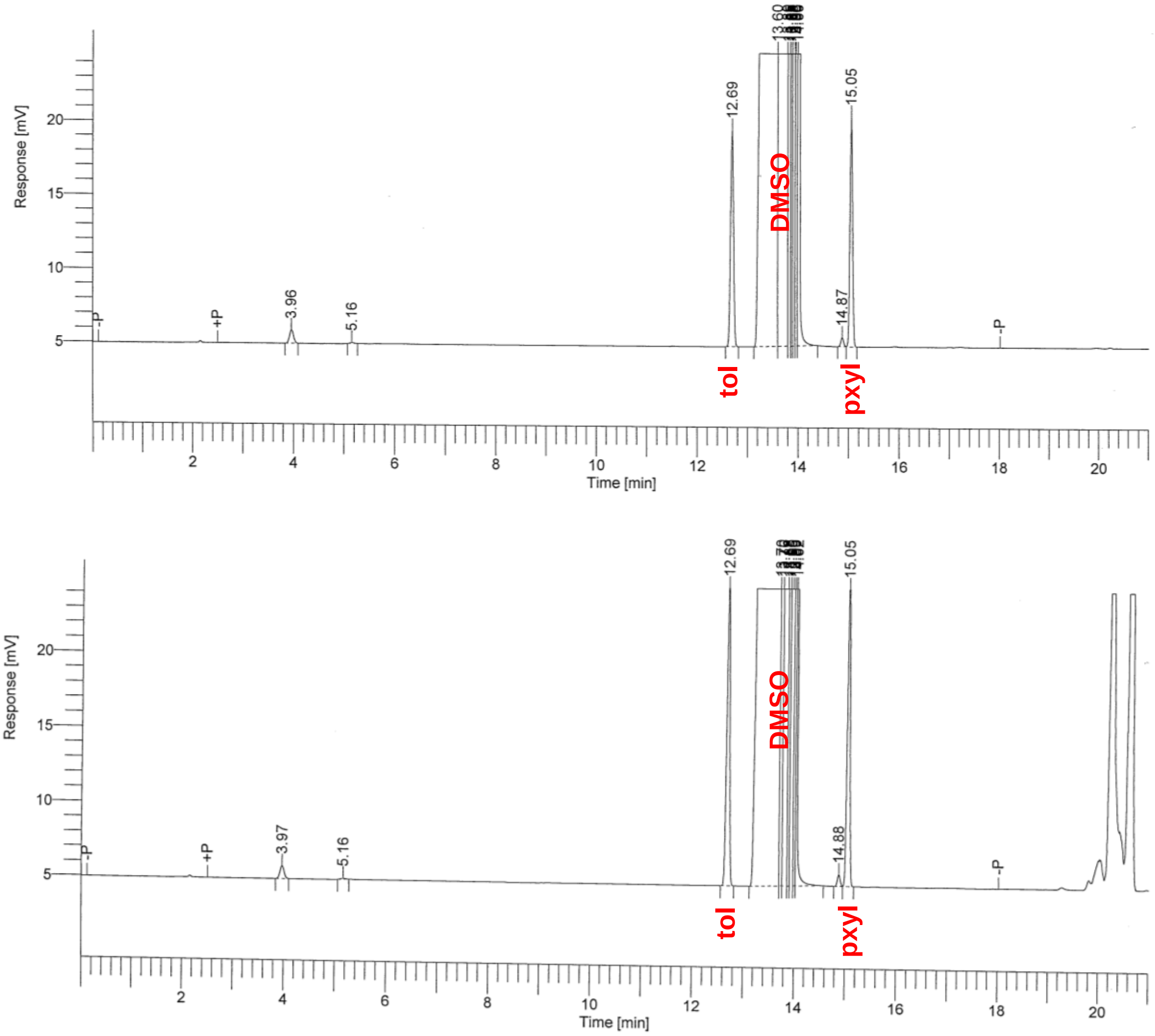

Figure S40. Annotated gas chromatograms in DMSO for **1.phen.tol$_x$.pxyl$_{(1-x)}$**.

Gas chromatograms, 1.phen.tol$_x$.pxyl$_{(1-x)}$ (x = 0.14 average by NMR/GC).

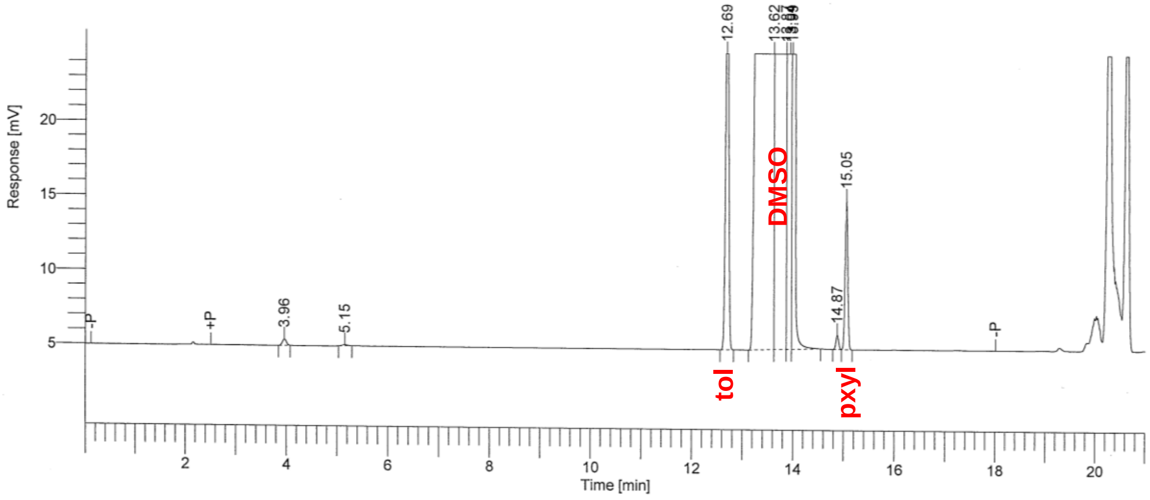

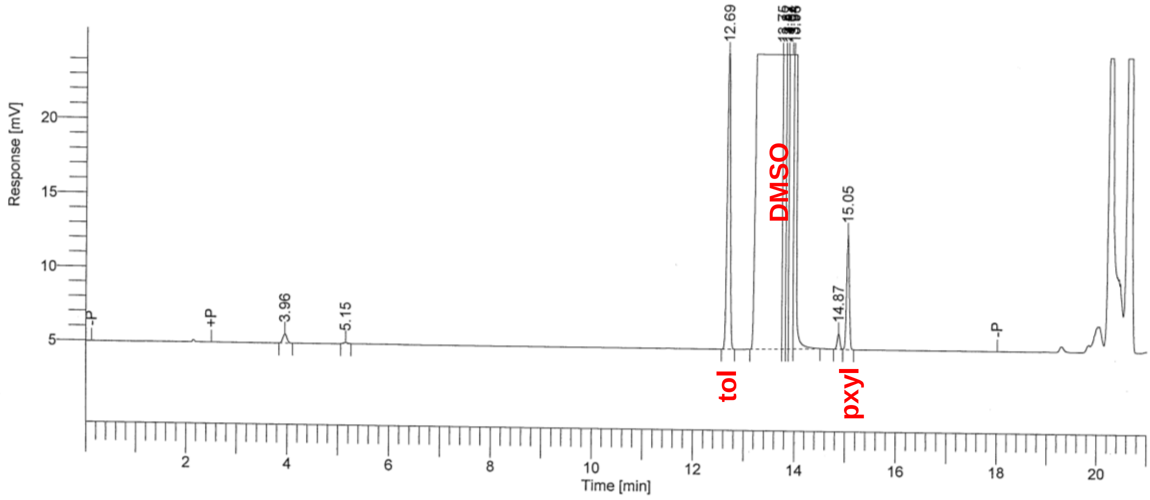

Figure S41. Annotated gas chromatograms in DMSO for **1.phen.tol$_x$.pxyl$_{(1-x)}$**.

5.2. Mixed toluene / other arene systems

Gas chromatograms, 1.phen.tol$_x$.oxyl$_{(1-x)}$ (x = 0.79 average by NMR/GC).

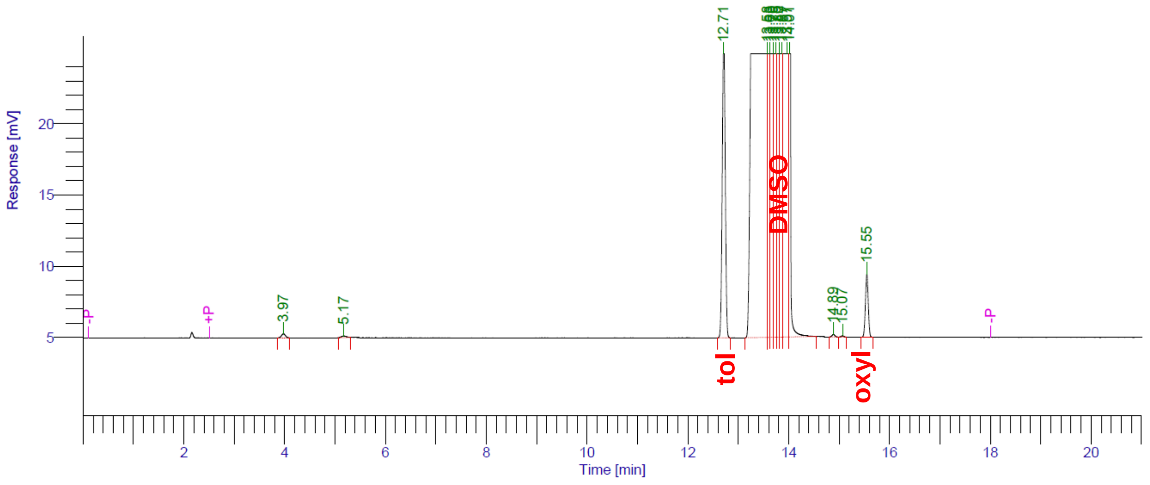

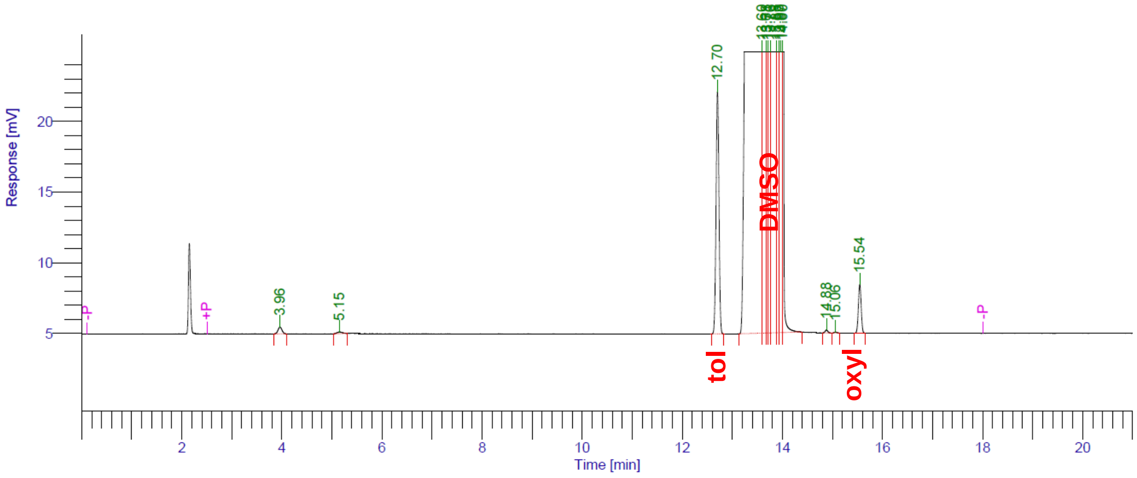

Figure S42. Annotated gas chromatograms in DMSO for **1.phen.tol$_x$.oxyl$_{(1-x)}$**.

Gas chromatograms, 1.phen.tol$_x$.mxyl$_{(1-x)}$ (x = 0.87 average by NMR/GC).

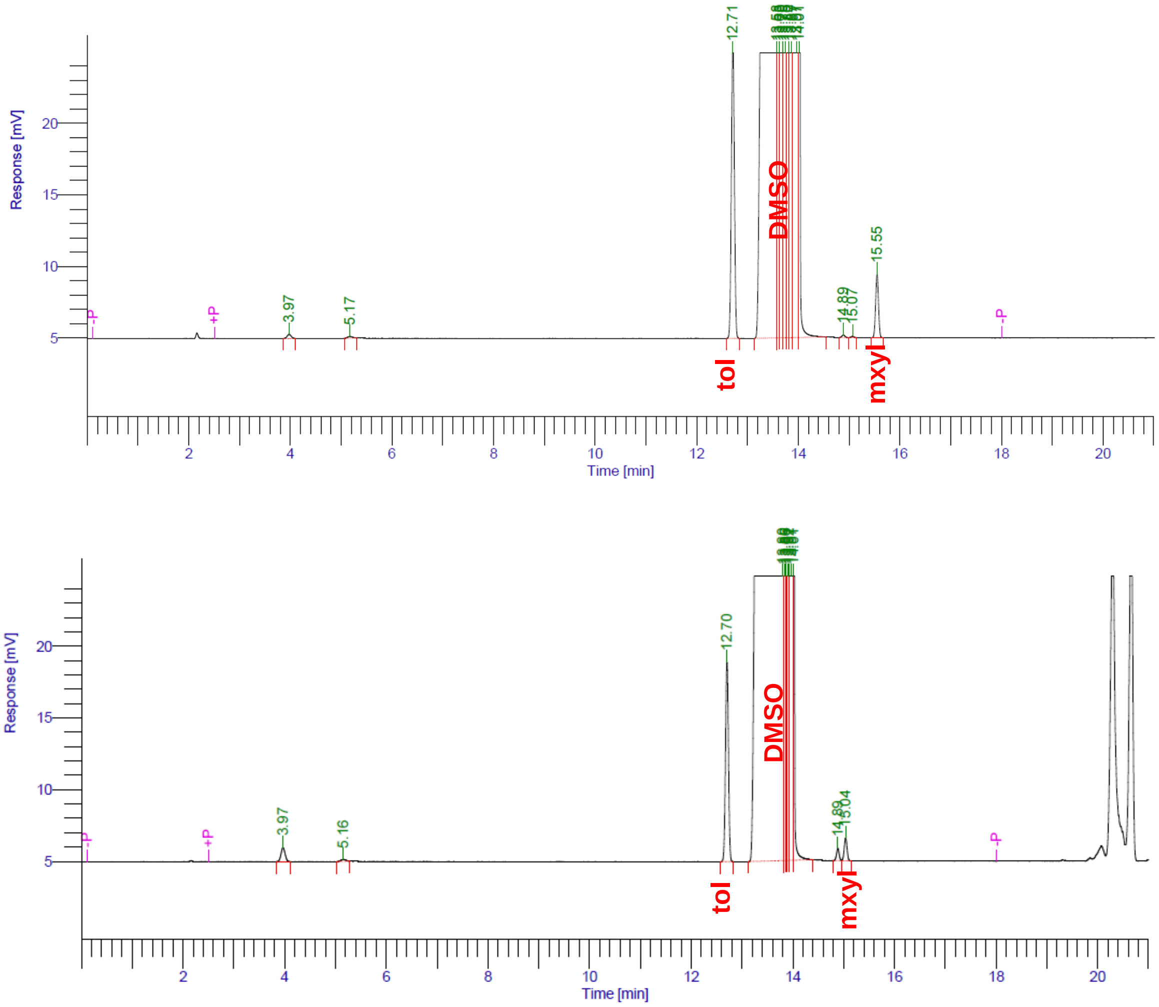

Figure S43. Annotated gas chromatograms in DMSO for **1.phen.tol$_x$.mxyl$_{(1-x)}$**.

Gas chromatograms, 1.phen.tol$_x$.C$_6$H$_{6(1\text{-}x)}$ (x = 0.46 average by NMR/GC).

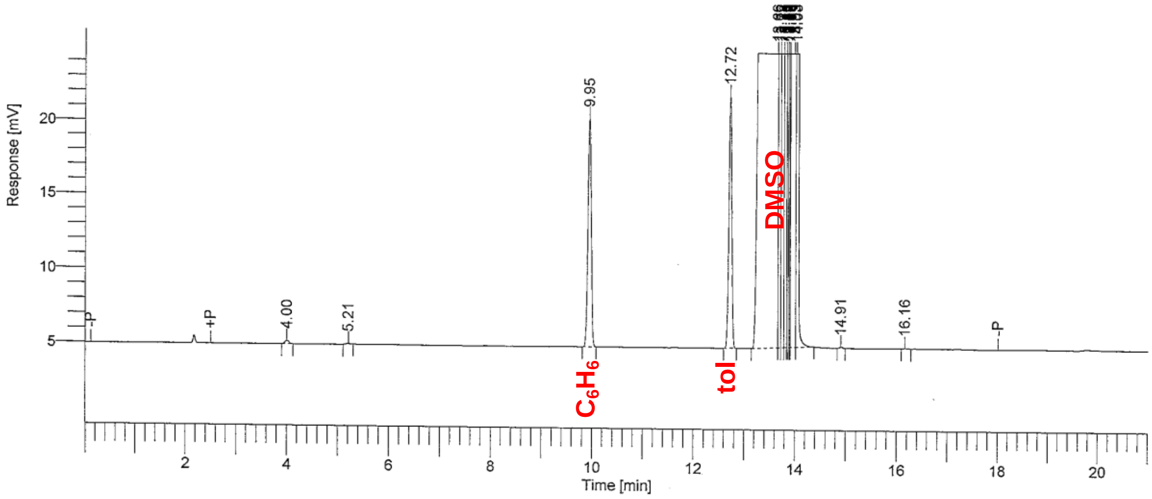

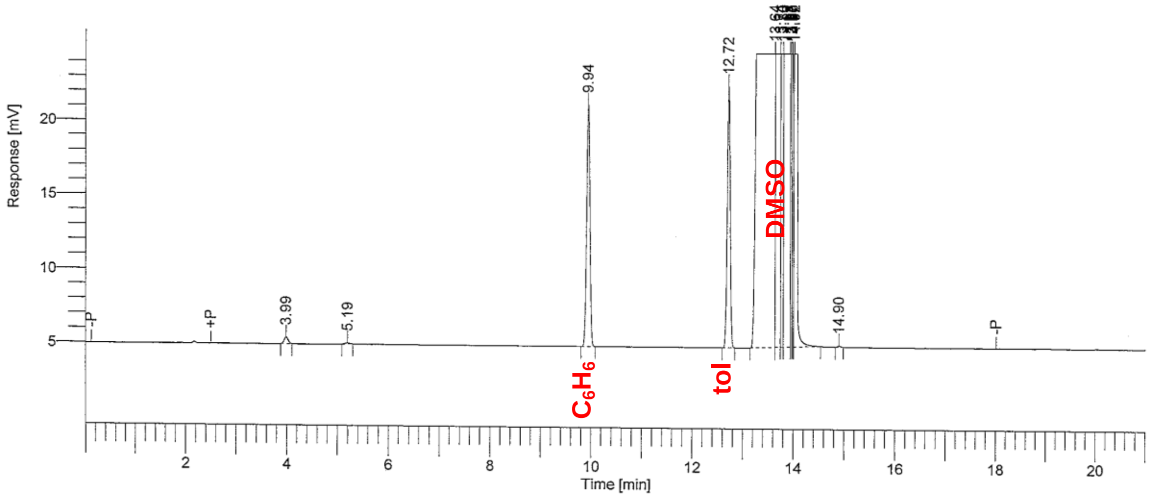

Figure S44. Annotated gas chromatograms in DMSO for **1.phen.tol$_x$.C$_6$H$_{6(1\text{-}x)}$**.

5.3. Mixed p-xylene / other arene systems

Gas chromatograms, 1.phen.pxyl$_x$.oxyl$_{(1\text{-}x)}$ (x = 0.90 average by NMR/GC).

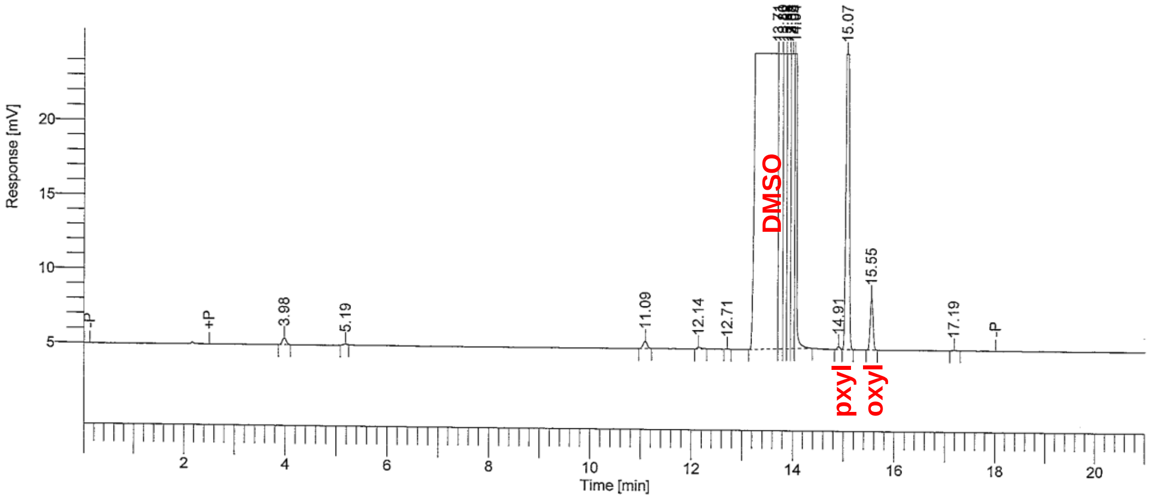

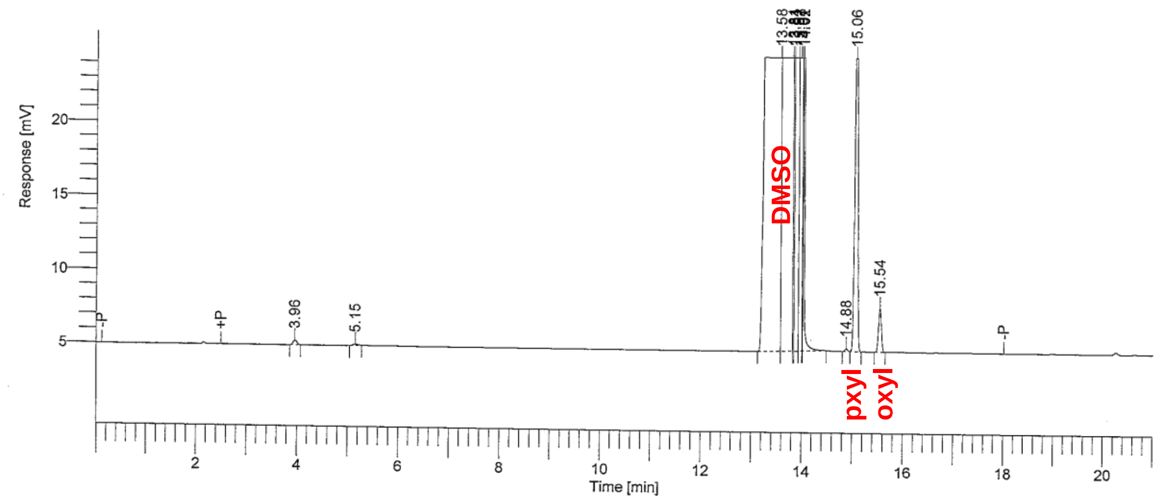

Figure S45. Annotated gas chromatograms in DMSO for **1.phen.pxyl$_x$.oxyl$_{(1-x)}$**.

Gas chromatograms, 1.phen.pxyl$_x$.mxyl$_{(1-x)}$ (x = 0.93 by NMR).

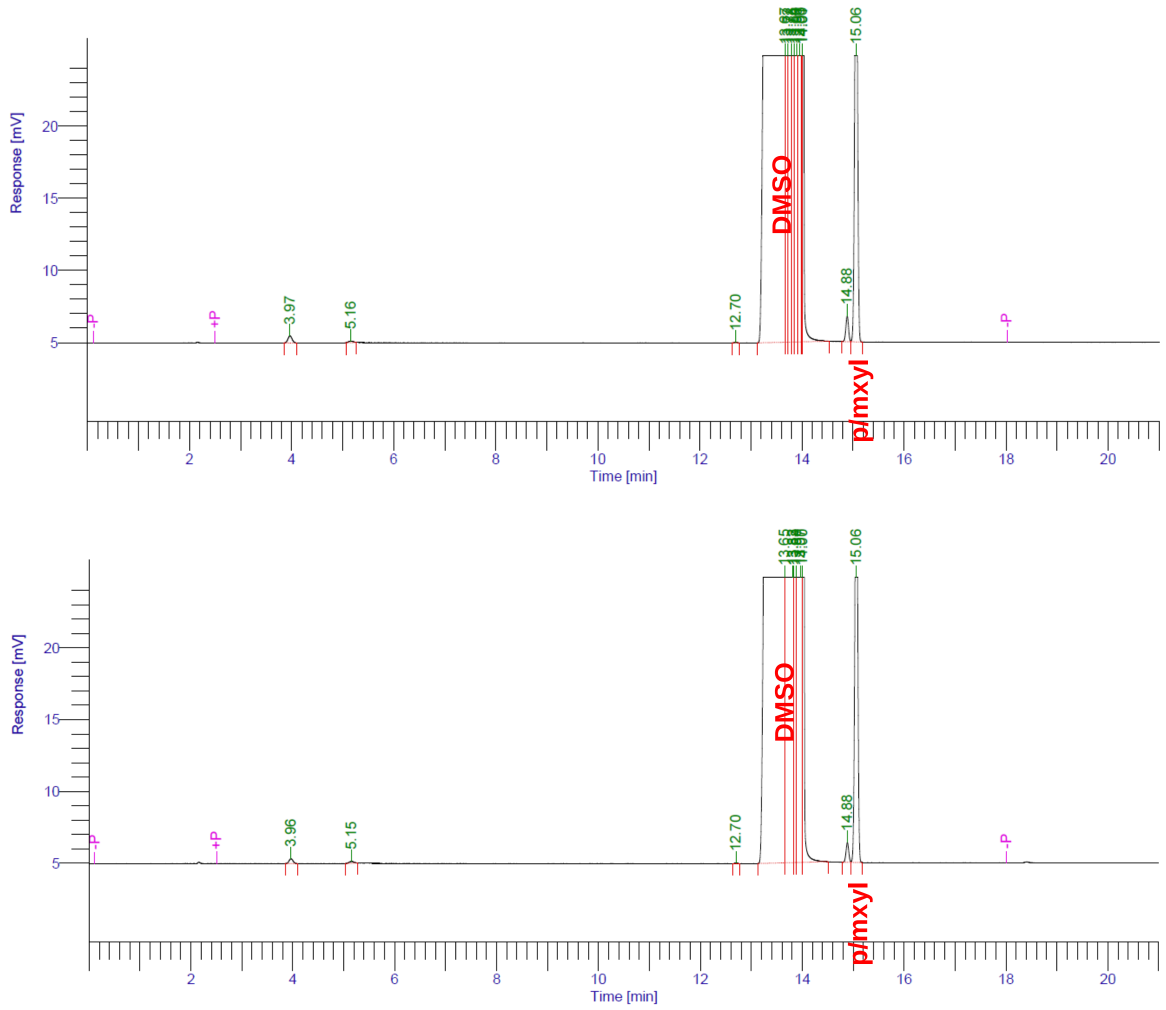

Figure S46. Annotated gas chromatograms in DMSO for **1.phen.pxyl$_x$.mxyl$_{(1-x)}$** (signals are indistinguishable).

Gas chromatograms, 1.phen.pxyl$_x$.C$_6$H$_{6(1-x)}$ (x = 0.58 average by NMR/GC).

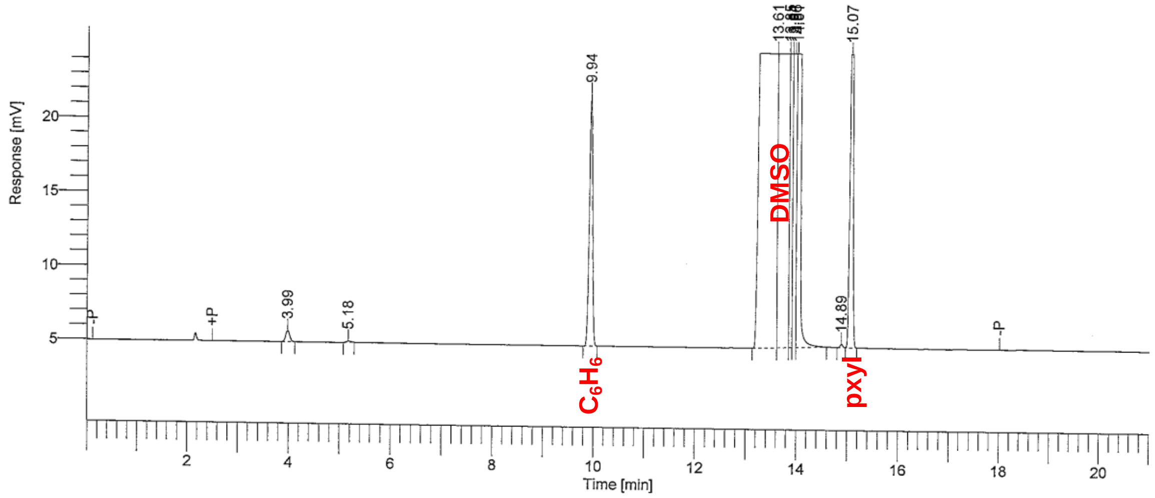

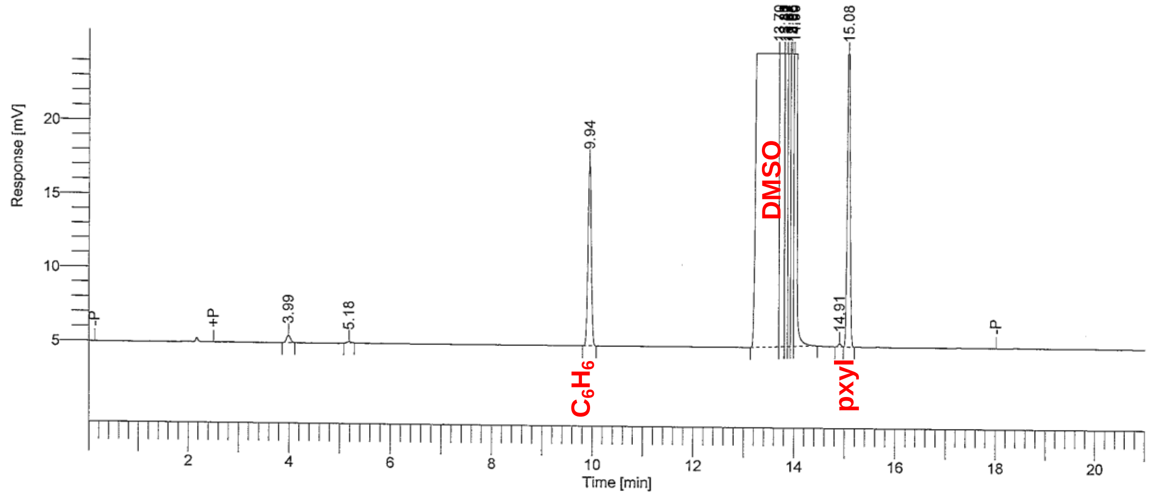

Figure S47. Annotated gas chromatograms in DMSO for **1.phen.pxyl$_x$. C$_6$H$_{6(1-x)}$.**

5.4. Mixed benzene / other arene systems

Gas chromatograms, 1.phen.C_6H_{6x}.oxyl$_{(1-x)}$ (x = 0.83 average by NMR/GC).

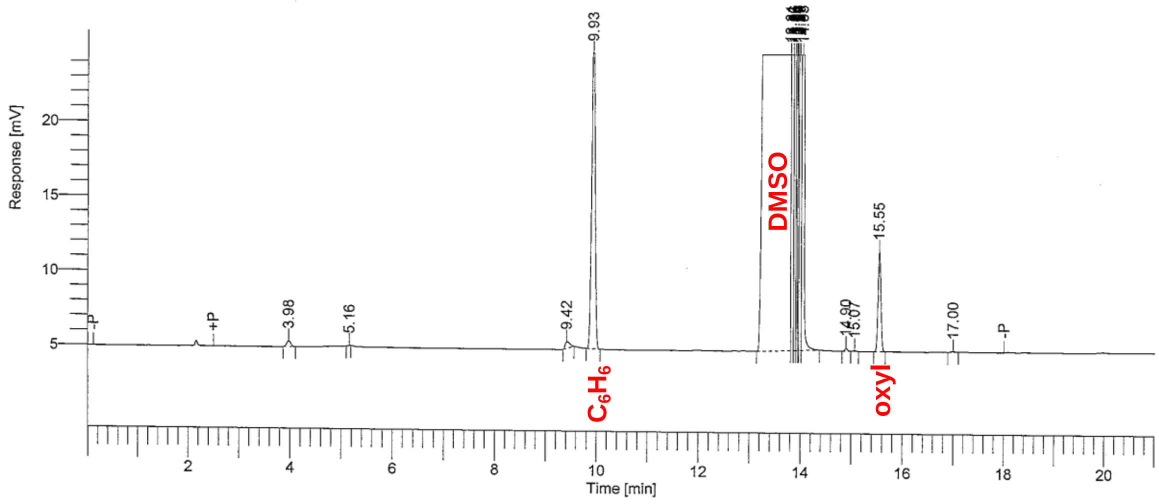

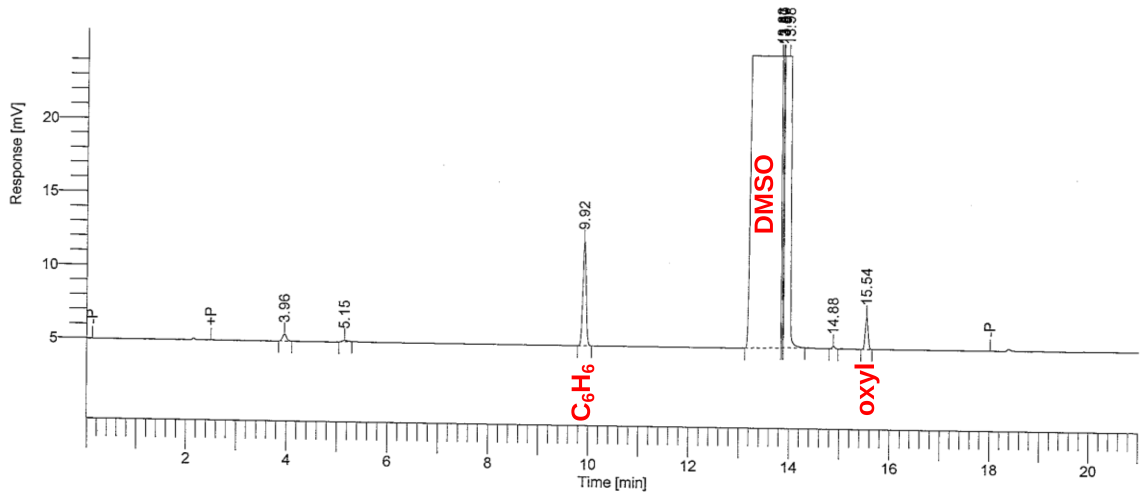

Figure S47. Annotated gas chromatograms in DMSO for **1.phen.C_6H_{6x}.oxyl$_{(1-x)}$**.

Gas chromatograms, 1.phen.C_6H_{6x}.mxyl$_{(1-x)}$ (x = 0.93 average by NMR/GC).

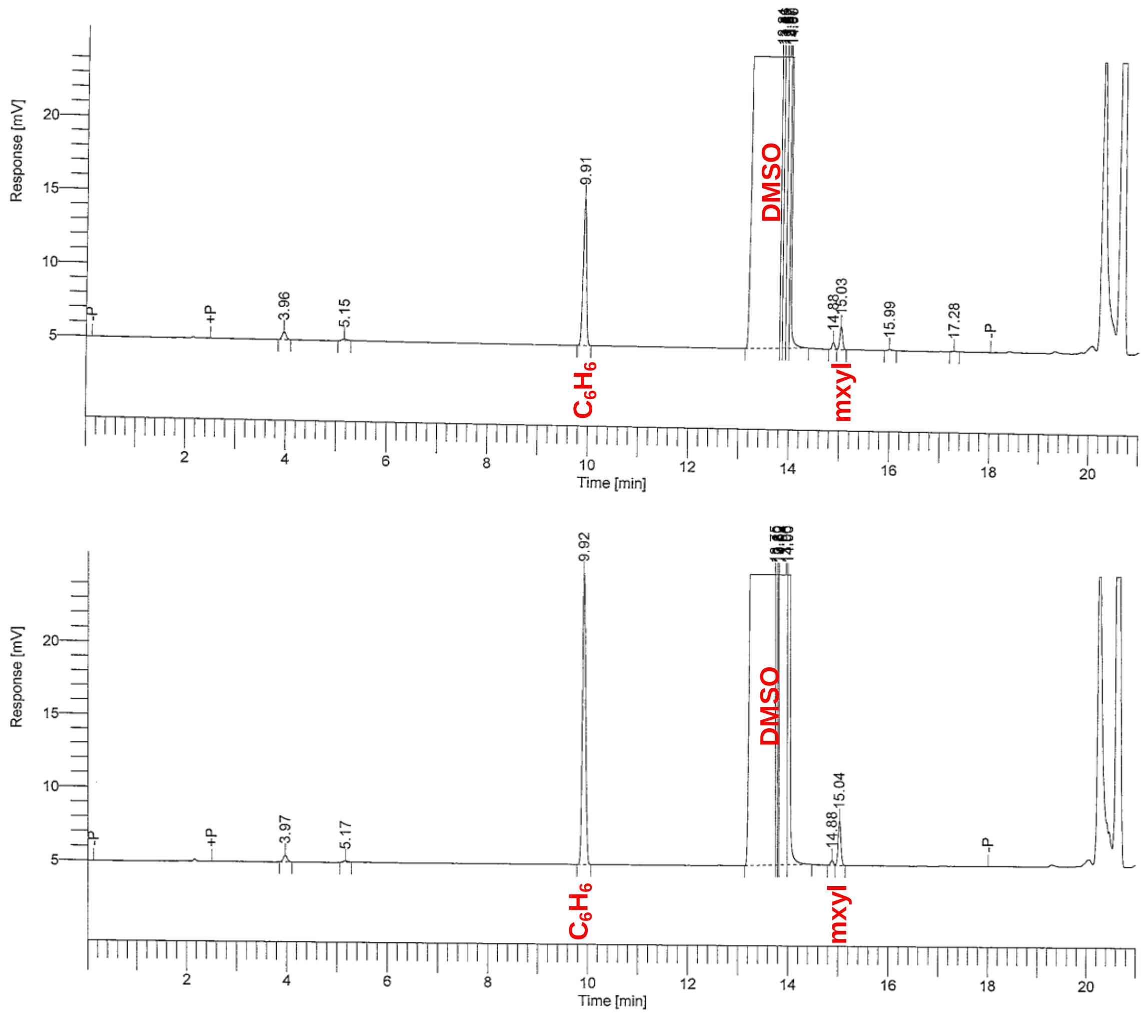

Figure S48. Annotated gas chromatograms in DMSO for **1.phen.C_6H_{6x}.mxyl$_{(1-x)}$**.

6. Selectivity constant calculations

The crystals resulting from pairwise competition experiments were filtered at the pump and allowed to air-dry for precisely five minutes. The crystals were split into two equal portions, and each portion immediately dissolved in d6-DMSO, to prevent further guest loss. Each portion was studied by both ^{1}H-NMR spectroscopy and GC, giving four measurements for each sample. Furthermore, each NMR spectrum was a source of up to two independent data points by comparing (where relevant or possible) the relative intensities of the signals for the aromatic protons and aliphatic protons separately, thus giving up to six data points for each sample. These data were used to calculate the selectivity coefficients. The reported value is the mean of the set of determinations.

7. References

S1. S. P. Thompson, J. E. Parker, J. Potter, T. P. Hill, A. Birt, T. M. Cobb, F. Yuan, and C. C. Tang, *Rev. Sci. Intrum.*, 2009, **80**, 075107.

S2. S. P. Thompson, J. E. Parker, J. Marchal, J. Potter, A. Birt, F. Yuan, R. D. Fearn, A. R. Lennie, S. R. Street, and C. C. Tang, *J. Synchrotron Rad*., 2011, **18**, 637.

S3. A. A. Coelho, *TOPAS Academic, Version 4.1*, 2007; see http://www.topas-academic.net.

S4. G. S. Pawley, *J. Appl. Crystallogr*., 1981, **14**, 357.

S5. H. M. Rietveld, *J. Appl. Crystallogr*., 1969, **2**, 65.
